# Prescribed Versus Taken Polypharmacy and Drug–Drug Interactions in Older Cardiovascular Patients during the COVID-19 Pandemic: Observational Cross-Sectional Analytical Study

**DOI:** 10.3390/jcm12155061

**Published:** 2023-08-01

**Authors:** Nina D. Anfinogenova, Oksana M. Novikova, Irina A. Trubacheva, Elena V. Efimova, Nazary P. Chesalov, Wladimir Y. Ussov, Aleksandra S. Maksimova, Tatiana A. Shelkovnikova, Nadezhda I. Ryumshina, Vadim A. Stepanov, Sergey V. Popov, Alexey N. Repin

**Affiliations:** 1 Cardiology Research Institute, Tomsk National Research Medical Center, Russian Academy of Sciences, Tomsk 634012, Russia; novom@cardio-tomsk.ru (O.M.N.); tia@cardio-tomsk.ru (I.A.T.); ev@cardio-tomsk.ru (E.V.E.); nazary.chesalov@gmail.com (N.P.C.); ussov1962@yandex.ru (W.Y.U.); asmaximova@yandex.ru (A.S.M.); fflly@mail.ru (T.A.S.); n.rumshina@list.ru (N.I.R.); psv@cardio-tomsk.ru (S.V.P.); ran@cardio-tomsk.ru (A.N.R.); 2 Meshalkin National Medical Research Center, Novosibirsk 630055, Russia; 3 Research Institute of Medical Genetics, Tomsk National Research Medical Center, Russian Academy of Sciences, Tomsk 634050, Russia; vadim-stepanov@medgenetics.ru

**Keywords:** cardiovascular disease, drug–drug interaction, polypharmacy, health information system, electronic health record, epidemiology, public health

## Abstract

The study aimed to assess clinical pharmacology patterns of prescribed and taken medications in older cardiovascular patients using electronic health records (EHRs) (*n* = 704) (2019–2022). Medscape Drug Interaction Checker was used to identify pairwise drug–drug interactions (DDIs). Prevalence rates of DDIs were 73.5% and 68.5% among taken and prescribed drugs, respectively. However, the total number of DDIs was significantly higher among the prescribed medications (*p* < 0.05). Serious DDIs comprised 16% and 7% of all DDIs among the prescribed and taken medications, respectively (*p* < 0.05). Median numbers of DDIs between the prescribed vs. taken medications were Me = 2, IQR 0–7 vs. Me = 3, IQR 0–7 per record, respectively. Prevalence of polypharmacy was significantly higher among the prescribed medications compared with that among the taken drugs (*p* < 0.05). Women were taking significantly more drugs and had higher prevalence of polypharmacy and DDIs (*p* < 0.05). No sex-related differences were observed in the list of prescribed medications. ICD code U07.1 (COVID-19, virus identified) was associated with the highest median DDI number per record. Further research is warranted to improve EHR structure, implement patient engagement in reporting adverse drug reactions, and provide genetic profiling of patients to avoid potentially serious DDIs.

## 1. Introduction

Multimorbidity is the coexistence of multiple health conditions potentially aggravating each other. A systematic review and meta-analysis of 126 studies showed that the global prevalence of multimorbidity is as high as 37.2%, and over half (51.0%) of the worldwide population aged 60 years and older has multimorbid conditions [1]. The prevalence of polypharmacy among patients of secondary-level hospital is 98%, with 5.1% having minor polypharmacy (two to three medications), 10% having moderate polypharmacy (four to five medications), and 83% having major polypharmacy (more than five medications) [2]. Up to 17% of older adults in Germany have at least one potential drug–drug interaction (DDI) [3]. Over half of nursing home residents (52.7%) are exposed to at least one DDI and 25.0% to more than one DDI [4]. Prevalence of DDIs in palliative care ranges from 31 to 75% across various health care settings [5]. In COVID-19 patients administered with ritonavir-containing therapy in the U.S., the weighted prevalence of major to contraindicated DDIs was 29.3%. Prevalence rates of DDIs among those 60 years and older with serious heart conditions, diabetes, and moderate chronic kidney disease are as high as 60.2%, 63.4%, and 80.7%, respectively [6]. 

Geriatric syndromes co-exist with acquired chronic diseases and contribute to multimorbidity. Multimorbidity predisposes a person to interactions between drugs administered for treatment of involved pathologies so that the resulting risk exceeds a simple summation of risks. Drug–drug interactions may lead to adverse drug reactions (ADRs), and medical error was reported to be the third leading cause of death in the U.S. [7]. Polypharmacy is associated with increased emergency department transfer in older long-term care residents, with the strength of association increasing with the number of medications prescribed [8]. 

One of the challenges facing healthcare today is the need for an interdisciplinary team-based approach to management of cardiovascular patients with multiple health conditions. Ideally, a cardiologist should be aware of therapies administered to patients by other medical specialists such as neurologists, endocrinologists, rheumatologists, and ophthalmologists. Administration of multiple medications is often unavoidable and, as a rule, beneficial to multimorbid patients, but the risks of potentially dangerous ADRs due to serious DDIs must be avoided. Evidence gaps exist in regard to the patterns of prescribed versus taken pharmacotherapy in older cardiovascular patients, especially during and after the COVID-19 pandemic when new drug combinations were introduced into clinical practice. 

Clinical pharmacology patterns of prescribed and taken medications in older cardiovascular patients may significantly depend on patient populations where physiologic and pathologic characteristics significantly vary. Due to interindividual variability, predictors of response to pharmacological treatment comprise not only gender and chronological age, but also past and present comorbidities, co-administration of medications, liver and kidney function, smoking, exercise, weight, eating and drinking behavior [9], genetics [10,11], and epigenetics [12]. Superposition of all these factors results in a unique pharmacokinetic and pharmacodynamic fingerprint of an individual. 

Studies investigating the patterns of prescribed and taken medications usually focus on patient compliance and adherence to treatment and are often limited to single-center experience and a narrow spectrum of disease entities. Little is known about the overall burden of prescribed versus taken polypharmacy and DDIs across the entire spectrum of medical care encounters including outpatient visits, home visits, and hospitalizations in older cardiovascular patients. 

The present study aimed to assess the patterns of DDIs and polypharmacy in older patients with cardiovascular diseases based on electronic health records (EHRs) stored in a health information system in 2019–2022. 

## 2. Materials and Methods

### 2.1. Ethics

This observational cross-sectional analytical study was performed in accordance with the standards of Good Clinical Practice and the Declaration of Helsinki. The study protocol was approved by the local Biomedical Ethics Committee (approval #230 from 28 June 2022). The present paper is the first of a planned series of articles reporting data from the study registered at ClinicalTrials.gov (Identifier NCT05336565). 

### 2.2. Inclusion Criteria

Inclusion criteria were the established diagnosis of cardiovascular disease, age of 75 years and older, and the presence of EHR in the regional health information system. 

### 2.3. Sample Characteristics 

**Figure 1:**
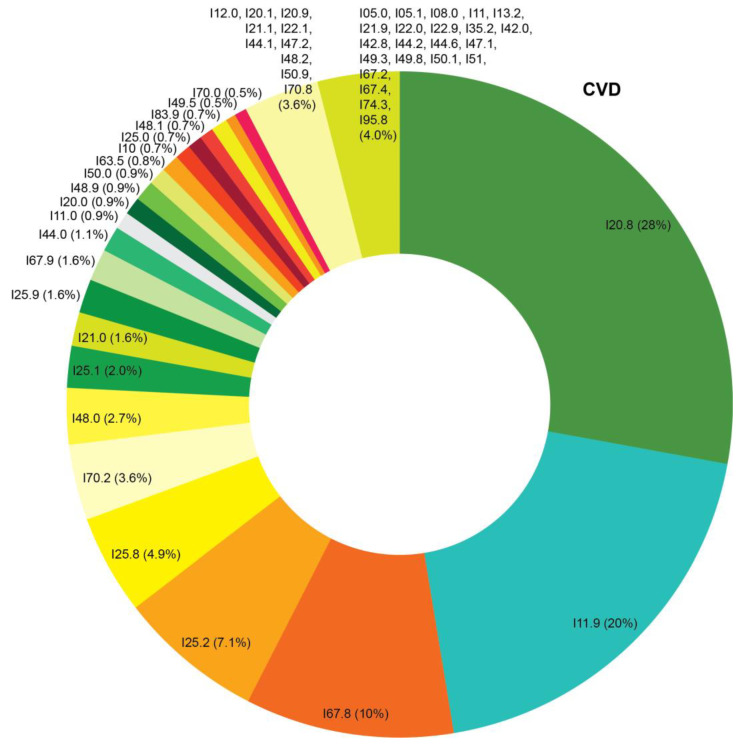
Structure of cardiovascular pathology in the study cohort according to data of electronic health records in 2019–2022. Detailed information on the clinical codes of International Statistical Classification of Diseases and Related Health Problems is available in Table S1.

**Figure 2:**
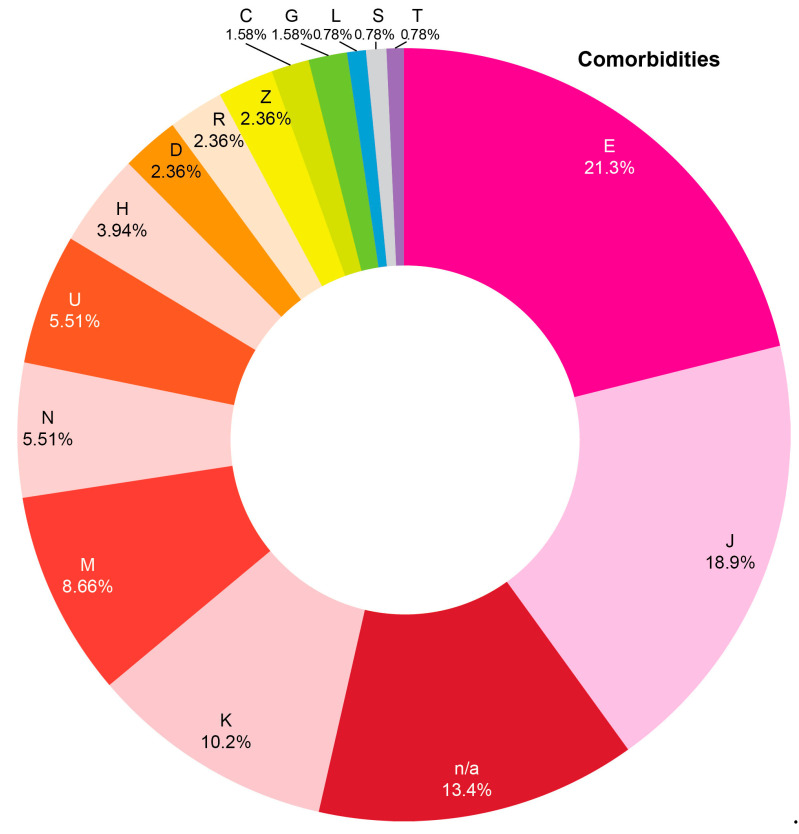
ICD structure defining morbidities except for the letter “I” (I00–I99) in the cohort of cardiovascular patients according to data of electronic health records in 2019–2022 (only the letters the ICD codes begin with are shown). E—endocrine, nutritional and metabolic diseases; J—diseases of the respiratory system; K—diseases of the digestive system; M—diseases of the musculoskeletal system and connective tissue; N—diseases of the genitourinary system; U—codes for special purposes; H—diseases of the eye and adnexa and diseases of the ear and mastoid process; D—diseases of the blood and blood-forming organs and certain disorders involving the immune mechanism; R—symptoms, signs and abnormal clinical and laboratory findings, not elsewhere classified; Z—factors influencing health status and contact with health services; C—neoplasms; G—diseases of the nervous system; L—diseases of the skin and subcutaneous tissue; S and T—injury, poisoning and certain other consequences of external causes; n/a—data not available. Detailed information on ICD codes is provided in Table S1.

The EHRs were obtained from the health information system implemented in 24 health care institutions of Tomsk and Tomsk Region. The EHRs covered the period from January 2019 to August 2022. The probability serial nested sampling method was used for patient selection. Cardiovascular diagnosis of patients was established and/or verified by a cardiologist. Figure 1 shows the cardiovascular pathology codes of the International Statistical Classification of Diseases and Related Health Problems (ICD) based on data available in the medical records. Baseline characteristics of patients and the clinical conditions recorded in the EHRs are presented in Table 1 and Table S1, respectively. Figure 2 demonstrates the ICD structure of morbidity, except for the letter “I” (I00–I99), as all patients had verified cardiovascular diagnosis established prior to the COVID-19 pandemic. Unstructured text of 704 EHRs was analyzed. Patient sex was identified based on the patient ID document presented for establishing the EHR. 

### 2.4. Medication Lists 

The analyzed EHRs contained unstructured textual information regarding medications taken by patients and medications prescribed to them during medical care encounters. 

Two large medication lists were established to characterize the entire cohort of cardiovascular patients, namely prescribed medication list (P-List) and taken medication list (T-List), with “P” and “T” standing for prescribed and taken medications. These lists comprised medications taken by or prescribed to patients over the entire cohort to assess DDIs and polypharmacy burden at the population level. 

The sub-lists of taken and prescribed medications were then established based on patient sex and the primary diagnosis ICD code. Combinations of drugs associated with individual medical care encounters were also analyzed. The individual lists of prescribed and taken medications often overlapped, but they were not identical to each other in every instant. 

### 2.5. Polypharmacy, DDIs, and DDI Index

Prevalence rates of DDIs and polypharmacy were expressed as percentages. The use of five medications or more was considered polypharmacy. Pairwise DDIs were identified and classified into contraindicated, serious, requiring close monitoring, and minor using Medscape Drug Interaction Checker [13]. 

Considering that individual records documented the use of drug combinations associated with multiple DDIs classified into four different categories, we developed a DDI index by introducing the following coefficients corresponding to drug impact categories: 1 (minor), 2 (monitor closely), 3 (serious), and 4 (contraindicated). The DDI index was calculated as the sum of relevant coefficients multiplied by the number of corresponding DDIs as follows:

$$DDI\;index=(4\times n_{contraindicated})+(3\times n_{serious})+(2\times n_{monitor-closely})+(1\times n_{minor})$$

### 2.6. Statistics 

#### 2.6.1. Sample Size Calculation

We assumed the prevalence of potentially serious DDIs ranging from 17% to 81% in patients during the pandemic [3,4,5,6]. We considered the acceptable margin of error of 5%, confidence level of 95%, and approximate population size of 20,000. Taking into account an assumed 18%-response distribution, we determined that a sample size of 225 would be sufficient to assess DDI patterns. We also performed the pilot study of polypharmacy rates in our cohort. Approximately 60% of the records contained data on prescribed polypharmacy. Polypharmacy in the list of taken medications was observed in every third EHR. Considering these rates, we increased the sample size to 704.

#### 2.6.2. Statistical Processing of Data

Statistical processing of data was performed using Microsoft Excel 2010 and STATISTICA 10 software. Figures were created using Microsoft Excel 2010, STATISTICA 10, and Adobe Illustrator. Normality of the distribution of variables was checked by the Kolmogorov–Smirnov test and the Shapiro–Wilk test. Data are presented as percentages, absolute numbers, mean ± standard deviation, and median and interquartile range where appropriate. Significance of differences between non-normally distributed variables was assessed by the Mann–Whitney U test. Significance of differences between normally distributed variables was assessed by Student’s *t*-test. Categorical variables were compared by the chi-square test using 2 × 2 contingency tables. Values were considered statistically significant when *p* was < 0.05.

## 3. Results

### 3.1. EHRs

Out of 704 EHRs analyzed, 38.1% of records belonged to men and 61.9% of records belonged to women. The records were created during ambulatory patient visits (*n* = 458), home visits by primary care physicians (*n* = 118), patient stays in emergency assessment units (*n* = 18), and hospital discharge procedures (discharge epicrisis records, *n* = 110) from January 2019 to August 2022. Information on prescribed drugs was present in 92.9% of EHRs; 51.7% of EHRs contained detailed information on drugs taken by patients. 

**Figure 3:**
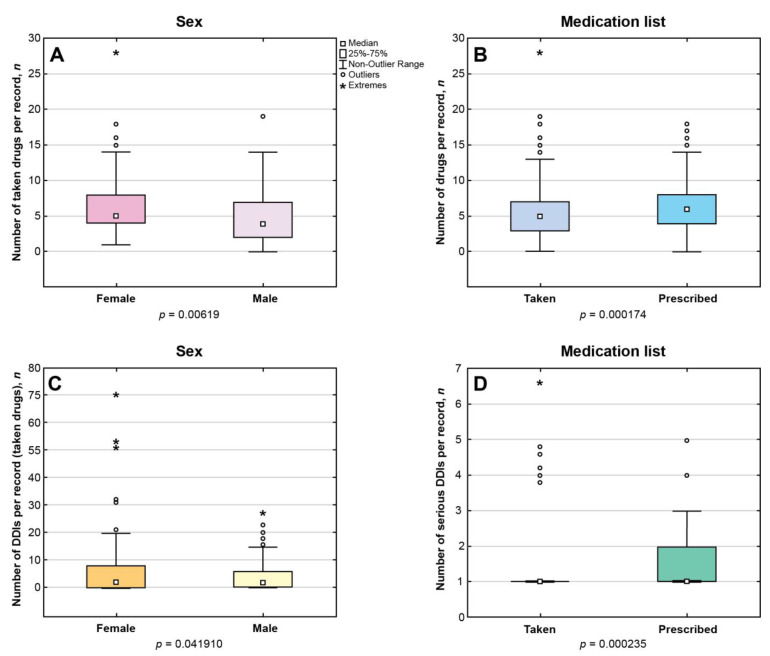
(**A**): Median number of taken medications per record in women versus men. (**B**): Median number of taken versus prescribed medications per record. (**C**): Median number of drug–drug interactions (DDIs) between taken medications in women versus men. (**D**): Median number of serious DDIs between taken versus prescribed medications per record. Only statistically significant results are presented (*p* < 0.05).

Among EHRs with documented information on pharmacotherapy, the number of medications per record ranged from 1 to 28 for the taken drugs (Me = 5, IQR 3–7; *n* = 361) and from 1 to 18 for the prescribed drugs (Me = 6, IQR 4–8; *n* = 651), *p* < 0.05. Female patients were taking significantly more drugs than men (*p* < 0.05) (Figure 3A). The number of medications per record in the P-List significantly exceeded the corresponding number in the T-List (*p* < 0.05) (Figure 3B).

### 3.2. Polypharmacy

In the case of polypharmacy, the median number of drugs prescribed to patient per record did not significantly differ from the median number of medications reported as “taken”: Me = 7, IQR 5–9 versus Me = 7, IQR 6–9 (*p* > 0.05). However, the prevalence of polypharmacy was significantly higher in the list of prescribed medications than in the list of taken medications (*p* < 0.05) (Table 2). 

Polypharmacy was observed significantly more often in women taking medications than in the corresponding group of men (*p* < 0.05). However, no sex-related differences were found in the rates of polypharmacy in the list of prescribed medications (Table 2). 

### 3.3. DDIs

**Figure 4:**
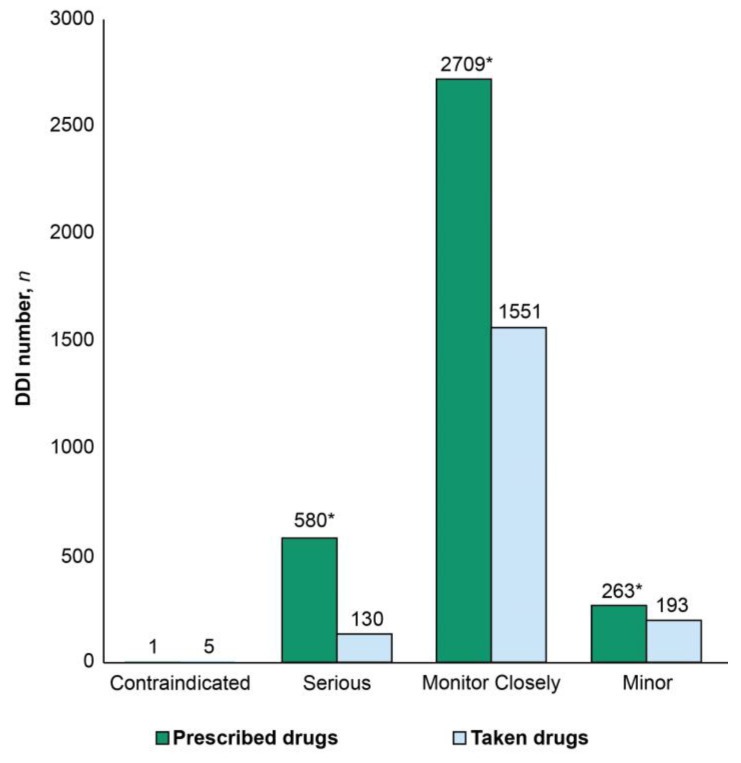
Numbers of drug–drug interactions (DDIs) classified into contraindicated, serious/dangerous, monitor closely, and minor in the lists of prescribed and taken medications. * *p* < 0.05.

The number of DDIs per record ranged from 0 to 70 and from 0 to 39 for the taken and prescribed medications, respectively. The prevalence rates of DDIs were 73.5% and 68.5% in the T- and P-Lists, respectively. Serious DDIs comprised 16% of all DDIs in the P-List and 7% of DDIs in the T-List (*p* < 0.05). Median DDI numbers per record were Me = 2, IQR 0–7 and Me = 3, IQR 0–7 in the T- and P-Lists, respectively. The total number of DDIs was significantly higher in the P-List compared with the T-List (*p* < 0.05) (Figure 4).

We identified 365 pairwise drug combinations associated with DDIs in the T-List, and the total number of DDI occurrences due to these combinations reached 1879. Among these, 249 drug combinations were associated with DDIs requiring close monitoring (*n* = 1551); 73 drug combinations were associated with minor DDIs (*n* = 193); 41 combinations were associated with serious DDIs (*n* = 130); and only two drug combinations were associated with contraindicated DDIs (*n* = 5). The top 10 serious and monitor-closely pairwise DDIs in the T-List are presented in Table 3 and Table 4, respectively. Detailed explanations of all pairwise DDIs in the T-list are given in Table S2 (serious DDIs), Table S3 (monitor-closely DDIs), and Table S4 (minor DDIs).

We identified 439 drug combinations associated with DDIs in the P-List, and these drug combinations resulted in more than seven-fold higher pairwise drug interactions (*n* = 3261). Among these, 317 drug combinations were associated with DDIs requiring close monitoring (*n* = 2709); 79 combinations were associated with minor DDIs (*n* = 261); 42 combinations were associated with serious DDIs (*n* = 290); and one combination was associated with contraindicated DDIs (*n* = 2). Female sex was associated with a significantly higher median number of DDIs between taken drugs compared with the corresponding number in males (*p* < 0.05) (Figure 3C). A significantly higher number of serious DDIs was identified in the P-List versus the T-List (*p* < 0.05) (Figure 3D). The top 10 serious and monitor-closely DDIs in the list of prescribed drugs are presented in Table 5 and Table 6, respectively. The detailed explanations of pairwise DDIs in the P-list are given in Table S5 (serious DDIs), Table S6 (monitor-closely DDIs), and Table S7 (minor DDIs). 

Contraindicated DDIs occurred between the following medications: “dexamethasone + apixaban” and “indapamide + sotalol” in the T-List and “indapamide + sotalol” in the P-List. Dexamethasone decreases the level or effect of apixaban by affecting hepatic/intestinal enzyme CYP3A4 metabolism, which reduces the anticoagulant effect by decreasing apixaban systemic exposure. Indapamide and sotalol both increase QTc interval. 

**Figure 5:**
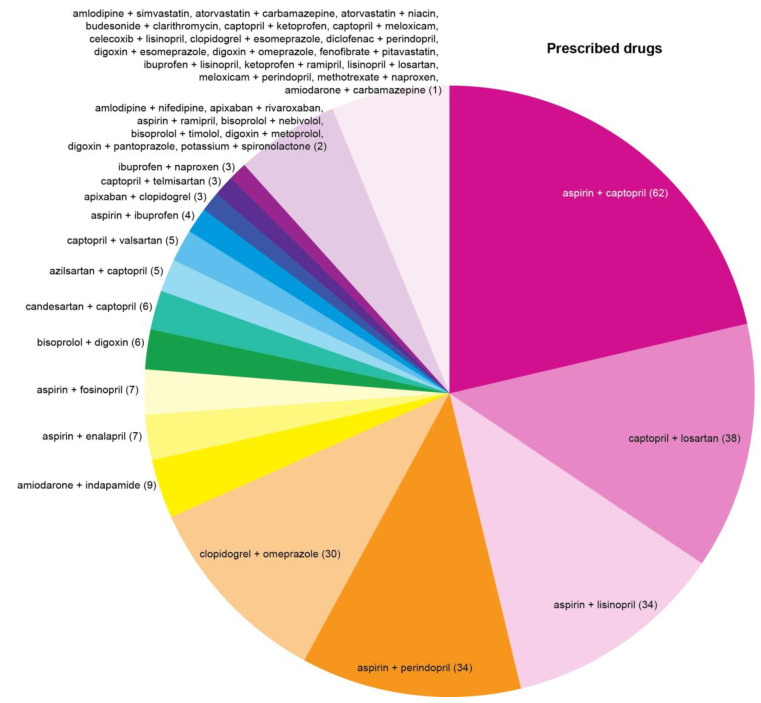
Pairwise combinations of prescribed drugs associated with serious/dangerous drug–drug interactions (DDIs) in the cohort of cardiovascular patients. Digits in parentheses indicate the absolute number of DDI occurrences for each pair of medications. Note: Impact of drug–drug interactions associated with the combinations “aspirin + captopril” and “aspirin + enalapril” may be considered insignificant due to the use of low-dose aspirin in the majority of cases. Administration of aspirin at doses less than 300 mg per day has little effect on the effectiveness of captopril and enalapril. Administration of aspirin in higher doses reduces the effectiveness of captopril and enalapril.

**Figure 6:**
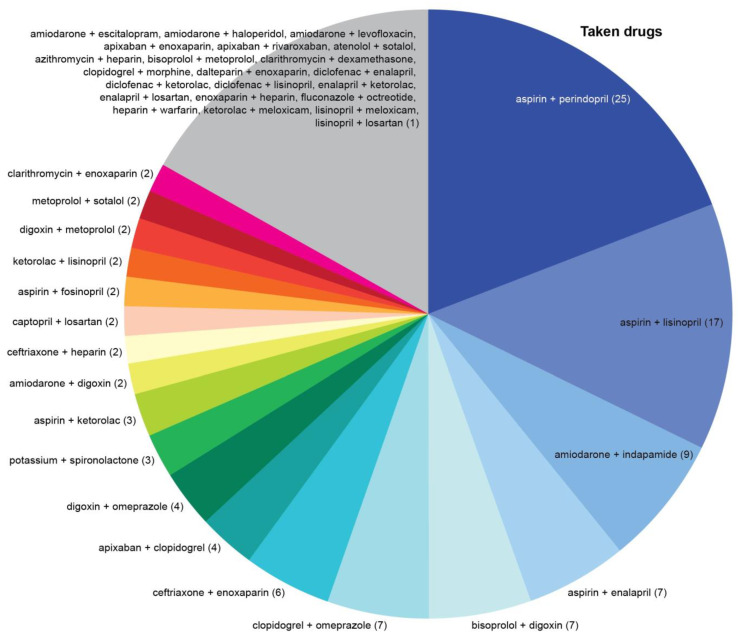
Pairwise combinations of taken drugs associated with serious/dangerous drug–drug interactions (DDIs) in the cohort of cardiovascular patients. Digits in parentheses indicate the absolute number of DDI occurrences for each pair of medications. Note: Impact of drug–drug interactions associated with the combinations “aspirin + captopril” and “aspirin + enalapril” may be considered insignificant due to the use of low-dose aspirin in the majority of cases. Administration of aspirin at doses less than 300 mg per day has little effect on the effectiveness of captopril and enalapril. Administration of aspirin in higher doses reduces the effectiveness of captopril and enalapril.

The top three most common drug combinations associated with serious/dangerous DDIs were “aspirin + captopril”, “captopril + losartan”, and “aspirin + lisinopril” in the P-List (Figure 5) and “aspirin + perindopril”, “aspirin + lisinopril”, and “amiodarone + indapamide” in the T-List (Figure 6). DDIs associated with the combinations “aspirin + captopril” and “aspirin + enalapril” may be considered clinically insignificant due to the use of low-dose aspirin in the majority of cases. Administration of aspirin at doses less than 300 mg per day has little effect on the effectiveness of captopril and enalapril. Administration of aspirin in higher doses reduces the effectiveness of captopril and enalapril.

The top three most common drug combinations associated with DDIs requiring close monitoring were “aspirin + bisoprolol”, “aspirin + losartan”, and “aspirin + metoprolol” in the P-List and “aspirin + losartan”, “aspirin + bisoprolol”, and “bisoprolol + losartan” in the T-List. Nine drugs (digoxin, amiodarone, enalapril, metoprolol, enoxaparin, ceftriaxone, ketorolac, heparin, and sotalol) were associated with significantly higher DDI numbers in the T-List compared with the P-List (*p* < 0.05). 

Only captopril and losartan were associated with significantly higher DDI numbers in the P-List relative to those in the T-List (*p* < 0.05), but the abundance of these DDIs contributed to significantly higher overall DDI burden among the prescribed drugs.

Three groups of DDI-associated drug combinations were identified: (1) only taken, but never recommended, (2) both taken and recommended, and (3) only recommended, but never taken.

### 3.4. DDI Index

**Figure 7:**
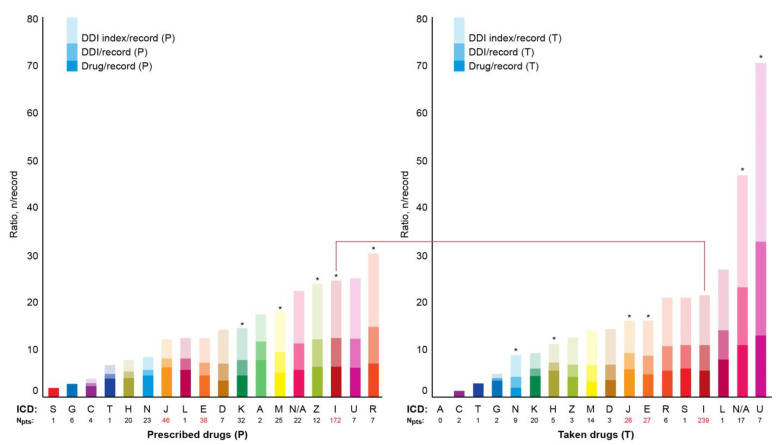
Median numbers of prescribed and taken medications and associated median numbers of drug–drug interactions (DDIs) and DDI indexes per record depending on primary ICD code (only the letters the ICD codes begin with are shown). Digits on the horizontal axis represent the corresponding numbers of patients in the electronic health records. Digits in red color on the horizontal axis highlight the top three most abundant ICD codes. The red line connects the bars corresponding to the most abundant ICD group of codes beginning with the letter “I”. Asterisk indicates significantly higher DDI index between the panels (* *p* < 0.05).

Figure 5 shows median numbers of prescribed and taken drugs and the corresponding values of DDIs and DDI indexes per record depending on the ICD code. Records without a specified ICD code were marked “N/A”. The DDI indexes ranged from 0 to 138. The top five DDI indexes in the P-List were associated with ICD codes beginning with the letters R, U, I, and Z, and N/A. The top five DDI indexes in the T-List were associated with ICD codes beginning with the letters U, N/A, L, I, and S (Figure 7). ICD code U07.1 (COVID-19, virus identified) was the only code beginning with the letter “U” in both lists (P- and T-Lists). DDI indexes associated with the N/A category ranked in the top two in the P-List and top five in the T-List (Figure 7). 

## 4. Discussion

Two primary drug lists (P-List and T-List, with “P” and “T” standing for prescribed and taken medications) were established in our study to analyze the patterns of prescribed and taken medications documented in the electronic health records in the cohort of older cardiovascular patients. A sub-list analysis enabled the assessment of the patterns of DDIs and polypragmasy at the group-based and individual levels. 

There are many medical decision support systems available to assess potential DDIs while prescribing pharmacotherapy [14,15,16,17,18]. Each of these systems has its own advantages and disadvantages, and several systems may be used for in-depth assessment of a limited number of DDIs. We selected a single medical decision support system for DDI assessment. Medscape Drug Interaction Checker [13] was chosen among other medical decision support systems because (i) it allowed stratification of the DDIs into four classes; (ii) it was user-friendly to operate; (iii) it provided information on underlying mechanisms of DDIs; and (iv) it was previously verified to be useful in assessing DDIs in cardiovascular and comorbid patients [19,20,21,22,23]. 

We analyzed DDIs on a pairwise basis because there are currently no commonly recognized resources allowing the assessment of higher-order DDIs, although such techniques are emerging and seem promising [24]. Pairwise DDI identification enabled the provision of straightforward and comprehensible illustrations, thereby contributing to better understanding of DDI patterns. In our study, median DDI number per record often exceeded the corresponding number of drug combinations because pharmacokinetics and pharmacodynamics of one pairwise drug combination involved more than one biotransformation pathway. 

We developed an easy-to-calculate DDI index to take into consideration the differential impact of DDI categories ranging from contraindicated to minor. Procedures currently accommodated by institutions for reporting ADRs are as follows: as soon as a healthcare provider becomes aware of an ADR, this information is recorded, and medication causing the ADR is discontinued or dosage adjustment is performed. Current procedures for reporting DDIs remain at the discretion of health care workers taking care of patients. No standard procedures for reporting DDIs have been implemented yet. For the first time, we propose the use of the DDI index to take into consideration the strength of drug interactions (contraindicated, serious/dangerous, requiring close monitoring, and minor). We believe that the simple summation of DDIs without introducing the DDI index could lead to underestimation of clinically significant DDI burden. Without introducing the DDI index, the contribution of minor and contraindicated DDIs (i.e., insignificant and very dangerous, respectively) to the overall DDI burden is leveled or equalized. We believe that DDIs should be stratified quantitively both for scientific purposes and clinical application. 

The proposed DDI index has not yet been validated. Upon validation, it may be implemented in clinical practice. Calculation of the proposed DDI index has been discussed with the institute running the study. The present research and introduction of the DDI index, in particular, represent the efforts aimed at the development of a strategy and measures to control clinically significant DDIs in our cardiovascular patients. We plan to further foster the concept of the DDI index in future research. Integrating quantitative systems’ pharmacology analysis with physiologically based pharmacokinetic models may lead to the development of more sophisticated scales. Multiscale modeling may predict potential pharmacodynamic DDIs, and, via clinical trial simulations, create testable hypotheses as to their potential clinical significance [25]. It is essential to develop clinical decision support systems for data-driven prediction of ADRs triggered by DDIs [26,27]. However, it seems challenging to adequately measure the overlapping impact of DDIs, which is multifactorial and depends on genetic factors, ADR manifestation, and economic burden. 

The frequency of occurrence of serious DDIs in our study was significantly higher in the case of prescribed medications compared with that among taken drugs. The highest median DDI index in our study was observed in patients with COVID-19. Notably, in the study by Spanakis and others [28], clinically significant DDIs of “serious-use alternative” or “use with caution-monitor” management were found in 40.3% of cases upon admission, 21% during hospitalization, and 40.7% upon discharge, suggesting that the efforts of a medical team can successfully reduce the risks associated with dangerous DDIs during a hospital stay. It is clear that serious DDIs can hinder treatment response and complicate hospitalization in COVID-19 patients [28]. 

Sex-related differences found in our study agree with data of the large-scale analysis showing that women have a 60% increased risk of DDI and a 90% increased risk of DDI leading to major ADR as compared to men [27]. Female sex and older age also contribute to non-adherence to, in particular, statins [29]. We agree that the potential effects of sex and gender on inappropriate prescribing and deprescribing remain poorly understood [30]. Cognitive, behavioral, pharmacokinetic, and pharmacodynamic factors of adaptation underlying significantly higher scores in drug numbers, polypharmacy rates, and DDIs in women require further research. 

Our study showed significant differences in the median numbers of serious DDIs per record (i.e., per single medical care encounter) in the lists of prescribed and taken medications. The mismatch of serious, requiring close monitoring, and minor DDIs was also found between the large cohort-based lists of prescribed and taken medications. This observation may indirectly suggest suboptimal treatment compliance and/or non-adherence of patients to prescribed therapy. Considering the significantly higher burden of serious DDIs among prescribed medications, the observed difference may be a sign of patient adaptation protecting them from exposure to serious DDIs. 

Among the most commonly prescribed drug combinations associated with serious DDIs, the pairs of “aspirin + captopril” and “aspirin + enalapril” may be considered clinically insignificant due to the use of low-dose aspirin in the majority of cases. Administration of aspirin at doses less than 300 mg per day has little effect on the effectiveness of captopril and enalapril. Administration of aspirin in higher doses reduces the effectiveness of captopril and enalapril. Furthermore, captopril was often prescribed to be taken episodically when blood pressure remained high, despite intake of other antihypertensives; this corresponds to the guidelines of the Russian Medical Society on Arterial Hypertension (RMSAH) [31], which recommend administration of relatively fast- and short-acting oral/sublingual angiotensin converting enzyme inhibitors (captopril, moxonidine, clonidine, and propranolol) for treatment of uncomplicated hypertensive crisis. It remains unclear whether the risk of taking these combinations may be completely dismissed considering the significant burden of polypharmacy and higher-order DDIs, which could potentially interfere with the pharmacokinetics of administered drugs. The combination “aspirin + lisinopril”, associated with serious DDIs, was among the most common in both lists (T- and P-Lists). Other common drug combinations associated with serious DDIs differed between the lists. 

Drug interactions observed in some patients could directly result from the official clinical recommendations regarding the treatment of certain disease entities. British researchers from seven medical centers performed a systematic study focusing on the recommendations given in twelve national clinical guidelines [32]. The analysis of drug–disease interactions and DDIs between medications recommended by national guidelines showed that following the guidelines may result in serious DDIs in multimorbid patients. The number of potentially serious (dangerous) DDIs reaches 30 in patients with most common multimorbidities, which poses a significant risk of serious ADRs including neurotoxicity, abnormal renal function, bleeding, and cardiovascular reactions [32]. Randomized clinical trials underlying guidelines produce high-quality data regarding the benefits rather than the risks of taking medication in real clinical settings where people are usually frailer and multimorbid and take multiple drugs for treatment of conditions distinct from those in clinical trial populations. Studying real groups of patients improves understanding of the heterogeneity of patient populations, and allows the development of measures that provide pure benefits without the harm posed by potentially serious DDIs [33]. Furthermore, paper versions of guidelines are challenging to integrate for people with geriatric syndromes and multimorbidity due to the overwhelming body of knowledge produced in recent years and the vast array of factors to be taken into consideration. Solving this problem would require going beyond the clinical recommendations in this category of patients.

The Working Group on Cardiovascular Pharmacotherapy of the European Society of Cardiology encourages implementation of a multidisciplinary team approach and consideration of age-related changes in the pharmacokinetics and pharmacodynamics of cardiovascular drugs to address the issues of polypharmacy [34]. The working group considers that adherence to pharmacotherapy is a key question. It is vital to thoroughly understand the most common ADRs, practices of deprescribing [34,35], problems of omissions, and potentially inappropriate medications, which may require going beyond the guidelines while implementing binary or multicore team-based approaches to care for vulnerable patients [36]. 

Genetic variations markedly increase or ameliorate the severity of potential DDIs and should be considered while prescribing pharmacotherapy to patients with polypharmacy. Most current guidelines on DDIs neither consider the potential effect of genetic polymorphisms in the strength of the interaction nor do they account for the complex interaction caused by the combination of DDIs and DGIs (drug–gene interactions) when there are multiple biotransformation pathways, which are referred to as DGGIs (drug–gene–gene interactions) [10]. The increasing availability of real-world drug outcome data linked to genetic technologies and resources is likely to enable the discovery of previously unrecognized clinically significant drug–drug–gene interactions to develop clinically useful models to reduce adverse DDIs and improve drug outcomes in the setting of increasing multimorbidity and polypharmacy [10,11]. 

Our study has some limitations. First, we studied the lists of prescribed and taken medications documented in the EHRs. These lists could differ from the drugs taken by patients in reality, especially in the case of medical records documenting patient visits to outpatient facilities, as it is possible that older individuals could misreport some of the medications they take. On the contrary, records of administrated medications during hospitalizations may be more precise. Furthermore, some community-dwelling older patients could practice self-administration of over-the-counter drugs, food supplements, and herbal medications without reporting it to their health care providers. Due to the retrospective nature of our study, there was no way to verify with certainty whether the drugs documented as those patients were taking had indeed been taken by patients. Future studies involving patient surveys may contribute to solving this issue. The second limitation is caused by the fact that the health information system covers only a portion of healthcare institutions in Tomsk and Tomsk Region, and this coverage will expand in the future. Therefore, the data in this paper represent only a portion of the patient population triaged to particular healthcare providers. The third limitation of the study is due to the significant disruption in routine healthcare during the COVID-19 pandemic, which provides a rationale for continuous monitoring of the situation with pharmacotherapy patterns in the vulnerable cohort of older cardiovascular patients. The fourth limitation is the absence of data on some medications, in particular, umifenovir and favipiravir in the Medscape Drug Interaction Checker at the time of investigation. However, these drugs constituted less than 1% of the entire pool of medications administered to our cohort, so they would not make a significant difference to the overall picture. Finally, we did not study the associations between DDIs and potential ADRs in our cohort, which require further independent research. 

We propose the following solutions to the problem of high DDI burden: (i) building a better structure of EHRs; (ii) patient engagement in medication diaries and ADR documentation using specially built portals linked to EHRs [37]; (iii) identifying patients with clinically significant polymorphisms of genes involved in drug metabolism [10,11]; (iv) developing electronic decision-making support system for control over DDIs; and (v) an interdisciplinary approach to team building [36]. Data such as those obtained in our study should urge the medical expert community to develop consensus guidelines for the pharmacotherapy of geriatric patients with multimorbidity. 

## 5. Conclusions

The high prevalence of serious DDIs and polypharmacy requires implementation of deprescribing protocols in older cardiovascular patients. Control of DDIs and polypharmacy may contribute to better medical compliance and adherence by reducing potential ADRs. Further research is warranted to improve the EHR structure, provide patient engagement in reporting ADRs via an EHR-linked platform, implement patient clustering and genotyping, develop an electronic decision support system, and practice interdisciplinary teamwork to ensure safe and effective personalized care.

## Figures and Tables

**Table 1:** Baseline characteristics of patient cohort based on data of electronic health records in 2019–2022 (*n* = 704).

Parameter	Value
Ethnic group, *n* (%):	
White/Caucasian, *n* (%)	704 (100%)
Sex	
Male, *n* (%)	268 (38.1%)
Female, *n* (%)	436 (61.9%)
Age, median (IQR), years	78 (75; 82)
Age of men, median (IQR), years	77.5 (75; 83)
Age of women, median (IQR), years	79 (75; 82)
Type of medical care encounter:	
Ambulatory visits, *n* (%)	458 (65.1)
Home visits, *n* (%)	118 (16.8)
Hospitalizations, *n* (%)	110 (15.6)
Emergency assessment unit visits, *n* (%)	18 (2.56)
Time of electronic health record registration	Jan 2019–Aug 2022

**Table 2:** Polypharmacy occurrence in the lists of taken (T-List) and prescribed medications (P-List) in male (m) and female (f) patients.

Medication List; Sex	Polypharmacy
T-List; m, *n*	69 *^§^
T-List; f, *n*	146 *^§^
P-List; m, *n*	166 ^§^
P-List; f, *n*	269 ^§^
T-List; m + f, *n*	215 ^§^
P-List; m + f, *n*	435 ^§^

Note: * *p* < 0.05 indicates significant differences between men and women; ^§^ *p* < 0.05 indicates significant differences between T- and P-Lists.

**Table 3:** Top 10 pairwise serious drug–drug interactions in the list of taken drugs in the cohort of cardiovascular patients based on data of electronic health records in 2019–2022 (*n* = 704).

Drug Combination	*n*	Top 10 Serious Drug–Drug Interactions (T-List)
aspirin + perindopril	25	Aspirin, perindopril. Pharmacodynamic antagonism. Avoid or Use Alternate Drug. Co-administration may result in a significant decrease in renal function. NSAIDs may diminish the antihypertensive effect of ACE inhibitors. The mechanism of these interactions is likely related to the ability of NSAIDs to reduce the synthesis of vasodilating renal prostaglandins
aspirin + lisinopril	17	Aspirin, lisinopril. Pharmacodynamic antagonism. Avoid or Use Alternate Drug. Co-administration may result in a significant decrease in renal function. NSAIDs may diminish the antihypertensive effect of ACE inhibitors. The mechanism of these interactions is likely related to the ability of NSAIDs to reduce the synthesis of vasodilating renal prostaglandins
amiodarone + indapamide	9	Amiodarone and indapamide both increase QTc interval. Avoid or Use Alternate Drug
aspirin + enalapril *	7	Aspirin, enalapril. Pharmacodynamic antagonism. Avoid or Use Alternate Drug. Co-administration may result in a significant decrease in renal function. NSAIDs may diminish the antihypertensive effect of ACE inhibitors. The mechanism of these interactions is likely related to the ability of NSAIDs to reduce the synthesis of vasodilating renal prostaglandins
bisoprolol + digoxin	7	Bisoprolol increases effects of digoxin by pharmacodynamic synergism. Use Caution/Monitor. Enhanced bradycardia
clopidogrel + omeprazole	7	Omeprazole decreases effects of clopidogrel by affecting hepatic enzyme CYP2C19 metabolism. Avoid or Use Alternate Drug. Clopidogrel efficacy may be reduced by drugs that inhibit CYP2C19. Inhibition of platelet aggregation by clopidogrel is entirely due to an active metabolite. Clopidogrel is metabolized to this active metabolite in part by CYP2C19
ceftriaxone + enoxaparin	6	Ceftriaxone increases effects of enoxaparin by anticoagulation. Avoid or Use Alternate Drug. cephalosporins may decrease prothrombin activity
apixaban + clopidogrel	4	Clopidogrel and apixaban both increase anticoagulation. Avoid or Use Alternate Drug
digoxin + omeprazole	4	Esomeprazole will increase the level or effect of digoxin by increasing gastric pH. Applies only to oral form of both agents. Avoid or Use Alternate Drug
aspirin + ketorolac	3	Aspirin, ketorolac. Either increases toxicity of the other by pharmacodynamic synergism. Contraindicated

* Note: Impact of drug–drug interactions associated with the combinations “aspirin + captopril” and “aspirin + enalapril” may be considered insignificant due to the use of low-dose aspirin in the majority of cases. Administration of aspirin at doses less than 300 mg per day has little effect on the effectiveness of captopril and enalapril. Administration of aspirin in higher doses reduces the effectiveness of captopril and enalapril.

**Table 4:** Top 10 pairwise monitor-closely drug–drug interactions among taken drugs in the cohort of cardiovascular patients based on data of electronic health records in 2019–2022 (*n* = 704).

Drug Combination	*n*	Top 10 Monitor-Closely Drug–Drug Interactions (T-List)
aspirin + losartan	104	Aspirin decreases effects of losartan by pharmacodynamic antagonism. Modify Therapy/Monitor Closely. NSAIDs decrease synthesis of vasodilating renal prostaglandins, and thus affect fluid homeostasis and may diminish antihypertensive effect
aspirin + bisoprolol	94	Aspirin decreases effects of bisoprolol by pharmacodynamic antagonism. Use Caution/Monitor. Long term (>1 wk) NSAID use. NSAIDs decrease prostaglandin synthesis
bisoprolol + losartan	58	Bisoprolol, losartan. Mechanism: pharmacodynamic synergism. Use Caution/Monitor. Risk of fetal compromise if given during pregnancy
aspirin + spironolactone	42	Aspirin decreases effects of spironolactone by unspecified interaction mechanism. Use Caution/Monitor. When used concomitantly, spironolactone dose may need to be titrated to higher maintenance dose and the patient should be observed closely to determine if the desired effect is obtained
amiodarone + losartan	32	Amiodarone will increase the level or effect of losartan by affecting hepatic enzyme CYP2C9/10 metabolism. Use Caution/Monitor. May inhibit the conversion of losartan to its active metabolite E-3174. Importance of interaction not established; monitor individual therapeutic response to determine losartan dosage
aspirin + metoprolol	32	Aspirin decreases effects of metoprolol by pharmacodynamic antagonism. Use Caution/Monitor. Long term (>1 wk) NSAID use. NSAIDs decrease prostaglandin synthesis
losartan + metoprolol	32	Losartan and metoprolol both increase serum potassium. Use Caution/Monitor
bisoprolol + torsemide	31	Bisoprolol increases and torsemide decreases serum potassium. Effect of interaction is not clear, use caution. Use Caution/Monitor
digoxin + spironolactone	30	Spironolactone increases levels of digoxin by Other (see comment). Use Caution/Monitor. Comment: Spironolactone may cause false elevation of digoxin assay
aspirin + perindopril	25	Aspirin, perindopril. pharmacodynamic antagonism. Avoid or Use Alternate Drug. Co-administration may result in a significant decrease in renal function. NSAIDs may diminish the antihypertensive effect of ACE inhibitors. The mechanism of these interactions is likely related to the ability of NSAIDs to reduce the synthesis of vasodilating renal prostaglandins

**Table 5:** Top 10 pairwise serious drug–drug interactions among prescribed drugs in the cohort of cardiovascular patients based on data of electronic health records in 2019–2022 (*n* = 704).

Drug Combination	*n*	Top 10 Serious Drug–Drug Interactions (P-List)
aspirin + captopril *	62	Aspirin, captopril. Pharmacodynamic antagonism. Avoid or Use Alternate Drug. Co-administration may result in a significant decrease in renal function. NSAIDs may diminish the antihypertensive effect of ACE inhibitors. The mechanism of these interactions is likely related to the ability of NSAIDs to reduce the synthesis of vasodilating renal prostaglandins
captopril + losartan	38	Losartan, captopril. Either increases toxicity of the other by pharmacodynamic synergism. Avoid or Use Alternate Drug. Dual blockade of renin-angiotensin system increases risks of hypotension, hyperkalemia, and renal impairment
aspirin + lisinopril	34	Aspirin, lisinopril. pharmacodynamic antagonism. Avoid or Use Alternate Drug. Co-administration may result in a significant decrease in renal function. NSAIDs may diminish the antihypertensive effect of ACE inhibitors. The mechanism of these interactions is likely related to the ability of NSAIDs to reduce the synthesis of vasodilating renal prostaglandins
aspirin + perindopril	34	Aspirin, perindopril. pharmacodynamic antagonism. Avoid or Use Alternate Drug. Co-administration may result in a significant decrease in renal function. NSAIDs may diminish the antihypertensive effect of ACE inhibitors. The mechanism of these interactions is likely related to the ability of NSAIDs to reduce the synthesis of vasodilating renal prostaglandins
clopidogrel + omeprazole	30	Omeprazole decreases effects of clopidogrel by affecting hepatic enzyme CYP2C19 metabolism. Avoid or Use Alternate Drug. Clopidogrel efficacy may be reduced by drugs that inhibit CYP2C19. Inhibition of platelet aggregation by clopidogrel is entirely due to an active metabolite. Clopidogrel is metabolized to this active metabolite in part by CYP2C19
amiodarone + indapamide	9	Amiodarone and indapamide both increase QTc interval. Avoid or Use Alternate Drug
aspirin + enalapril *	7	Aspirin, enalapril. pharmacodynamic antagonism. Avoid or Use Alternate Drug. Co-administration may result in a significant decrease in renal function. NSAIDs may diminish the antihypertensive effect of ACE inhibitors. The mechanism of these interactions is likely related to the ability of NSAIDs to reduce the synthesis of vasodilating renal prostaglandins
aspirin + fosinopril	7	Aspirin, fosinopril. pharmacodynamic antagonism. Avoid or Use Alternate Drug. Co-administration may result in a significant decrease in renal function. NSAIDs may diminish the antihypertensive effect of ACE inhibitors. The mechanism of these interactions is likely related to the ability of NSAIDs to reduce the synthesis of vasodilating renal prostaglandins
bisoprolol + digoxin	6	Bisoprolol increases effects of digoxin by pharmacodynamic synergism. Use Caution/Monitor. Enhanced bradycardia
candesartan + captopril	6	Candesartan, captopril. Either increases toxicity of the other by pharmacodynamic synergism. Avoid or Use Alternate Drug. Dual blockade of renin-angiotensin system increases risks of hypotension, hyperkalemia, and renal impairment

* Note: Impact of drug–drug interactions associated with the combinations “aspirin + captopril” and “aspirin + enalapril” may be considered insignificant due to the use of low-dose aspirin in the majority of cases. Administration of aspirin at doses less than 300 mg per day has little effect on the effectiveness of captopril and enalapril. Administration of aspirin in higher doses reduces the effectiveness of captopril and enalapril.

**Table 6:** Top 10 pairwise monitor-closely drug–drug interactions among prescribed drugs in the cohort of cardiovascular patients based on data of electronic health records in 2019–2022 (*n* = 704).

Drug Combination	*n*	Top 10 Monitor-Closely Drug–Drug Interactions (P-List)
aspirin + bisoprolol	180	Aspirin decreases effects of bisoprolol by pharmacodynamic antagonism. Use Caution/Monitor. Long term (>1 wk) NSAID use. NSAIDs decrease prostaglandin synthesis
aspirin + losartan	168	Aspirin decreases effects of losartan by pharmacodynamic antagonism. Modify Therapy/Monitor Closely. NSAIDs decrease synthesis of vasodilating renal prostaglandins, and thus affect fluid homeostasis and may diminish antihypertensive effect
aspirin + metoprolol	86	Aspirin decreases effects of metoprolol by pharmacodynamic antagonism. Use Caution/Monitor. Long term (>1 wk) NSAID use. NSAIDs decrease prostaglandin synthesis
bisoprolol + losartan	82	Bisoprolol, losartan. Mechanism: pharmacodynamic synergism. Use Caution/Monitor. Risk of fetal compromise if given during pregnancy
aspirin + captopril *	62	Aspirin, captopril. pharmacodynamic antagonism. Avoid or Use Alternate Drug. Co-administration may result in a significant decrease in renal function. NSAIDs may diminish the antihypertensive effect of ACE inhibitors. The mechanism of these interactions is likely related to the ability of NSAIDs to reduce the synthesis of vasodilating renal prostaglandins
aspirin + spironolactone	62	Aspirin decreases effects of spironolactone by unspecified interaction mechanism. Use Caution/Monitor. When used concomitantly, spironolactone dose may need to be titrated to higher maintenance dose and the patient should be observed closely to determine if the desired effect is obtained
aspirin + nitroglycerin	61	Aspirin increases effects of nitroglycerin sublingual by additive vasodilation. Use Caution/Monitor. Vasodilatory and hemodynamic effects of NTG may be enhanced by co-administration with aspirin (additive effect desirable for emergent treatment)
spironolactone + torsemide	56	Spironolactone increases and torsemide decreases serum potassium. Effect of interaction is not clear, use caution. Modify Therapy/Monitor Closely
bisoprolol + torsemide	55	Bisoprolol increases and torsemide decreases serum potassium. Effect of interaction is not clear, use caution. Use Caution/Monitor
aspirin + clopidogrel	46	Aspirin, clopidogrel. Either increases toxicity of the other by pharmacodynamic synergism. Use Caution/Monitor. The need for simultaneous use of low-dose aspirin and anticoagulant or antiplatelet agents are common for patients with cardiovascular disease; monitor closely

* Note: Impact of drug–drug interactions associated with the combinations “aspirin + captopril” and “aspirin + enalapril” may be considered insignificant due to the use of low-dose aspirin in the majority of cases. Administration of aspirin at doses less than 300 mg per day has little effect on the effectiveness of captopril and enalapril. Administration of aspirin in higher doses reduces the effectiveness of captopril and enalapril.

## Data Availability

Not applicable.

## Supplementary Materials

The following supporting information can be downloaded at: https://www.mdpi.com/article/10.3390/jcm12155061/s1, Table S1: Codes of International Statistical Classification of Diseases and Related Health Problems in the study cohort according to data of electronic health records in 2019–2022 (*n* = 704); Table S2: The detailed explanation of the entire list of pairwise serious drug–drug interactions among taken drugs in the cohort of cardiovascular patients based on data of electronic health records in 2019–2022 (*n* = 704); Table S3: The detailed explanation of the entire list of pairwise monitor-closely drug–drug interactions among taken drugs in the cohort of cardiovascular patients based on data of electronic health records in 2019–2022 (*n* = 704).; Table S4: The detailed explanation of the entire list of pairwise minor drug–drug interactions among taken drugs in the cohort of cardiovascular patients based on data of electronic health records in 2019–2022 (*n* = 704); Table S5: The detailed explanation of the entire list of pairwise serious drug–drug interactions among prescribed drugs in the cohort of cardiovascular patients based on data of electronic health records in 2019–2022 (*n* = 704).; Table S6: The detailed explanation of the entire list of pairwise monitor–closely drug–drug interactions among prescribed drugs in the cohort of cardiovascular patients based on data of electronic health records in 2019–2022 (*n* = 704).; Table S7: The detailed explanation of the entire list of pairwise minor drug–drug interactions among prescribed drugs in the cohort of cardiovascular patients based on data of electronic health records in 2019–2022 (*n* = 704).

## References

[1] Chowdhury S.R., Chandra Das D., Sunna T.C., Beyene J., Hossain A. (2023). Global and regional prevalence of multimorbidity in the adult population in community settings: A systematic review and meta-analysis. eClinicalMedicine.

[2] Alhumaidi R.M., Bamagous G.A., Alsanosi S.M., Alqashqari H.S., Qadhi R.S., Alhindi Y.Z., Ayoub N., Falemban A.H. (2023). Risk of Polypharmacy and Its Outcome in Terms of Drug Interaction in an Elderly Population: A Retrospective Cross-Sectional Study. J. Clin. Med..

[3] Reinhild Haerig T., Krause D., Klaassen-Mielke R., Rudolf H., Trampisch H.J., Thuermann P. (2023). Potentially inappropriate medication including drug-drug interaction and the risk of frequent falling, hospital admission, and death in older adults—Results of a large cohort study (getABI). Front. Pharmacol..

[4] Lion S., Evrard P., Foulon V., Spinewine A. (2023). Drug-drug interactions in nursing home residents: Analysis from the COME-ON trial. Age Ageing.

[5] Falconi G., Kashan S. (2023). Drug Interactions in Palliative Care. StatPearls.

[6] Igho-Osagie E., Brzozowski K., Jin H., Brown J., Williams M.G., Puenpatom A. (2023). Prevalence of Potential Drug-drug Interactions With Ritonavir-containing COVID-19 Therapy in the United States: An Analysis of the National Health and Nutrition Examination Survey. Clin. Ther..

[7] Makary M.A., Daniel M. (2016). Medical error-the third leading cause of death in the US. BMJ.

[8] Marincowitz C., Preston L., Cantrell A., Tonkins M., Sabir L., Mason S. (2022). Factors associated with increased Emergency Department transfer in older long-term care residents: A systematic review. Lancet Healthy Longev..

[9] Cerreta F., Vučić K., Laslop A. (2023). Assessing Medicines for Use in the Geriatric Population. Clin. Pharmacol. Ther..

[10] Hahn M., Roll S.C. (2021). The Influence of Pharmacogenetics on the Clinical Relevance of Pharmacokinetic Drug-Drug Interactions: Drug-Gene, Drug-Gene-Gene and Drug-Drug-Gene Interactions. Pharmaceuticals.

[11] Malki M.A., Pearson E.R. (2020). Drug-drug-gene interactions and adverse drug reactions. Pharmacogenomics J..

[12] Veiga-Matos J., Remião F., Motales A. (2020). Pharmacokinetics and Toxicokinetics Roles of Membrane Transporters at Kidney Level. J. Pharm. Pharm. Sci..

[13] Medscape Drug Interaction Checker. https://reference.medscape.com/drug-interactionchecker.

[14] Vonbach P., Dubied A., Krähenbühl S., Beer J.H. (2008). Evaluation of frequently used drug interaction screening programs. Pharm. World Sci..

[15] Marcath L.A., Xi J., Hoylman E.K., Kidwell K.M., Kraft S.L., Hertz D.L. (2018). Comparison of Nine Tools for Screening Drug-Drug Interactions of Oral Oncolytics. J. Oncol. Pract..

[16] Hecker M., Frahm N., Bachmann P., Debus J.L., Haker M.C., Mashhadiakbar P., Langhorst S.E., Baldt J., Streckenbach B., Heidler F. (2022). Screening for severe drug-drug interactions in patients with multiple sclerosis: A comparison of three drug interaction databases. Front. Pharmacol..

[17] Amkreutz J., Koch A., Buendgens L., Trautwein C., Eisert A. (2017). Clinical decision support systems differ in their ability to identify clinically relevant drug interactions of immunosuppressants in kidney transplant patients. J. Clin. Pharm. Ther..

[18] Kheshti R., Aalipour M., Namazi S. (2016). A comparison of five common drug-drug interaction software programs regarding accuracy and comprehensiveness. J. Res. Pharm. Pract..

[19] Das B., Ramasubbu S.K., Agnihotri A., Kumar B., Rawat V.S. (2021). Leading 20 drug-drug interactions, polypharmacy, and analysis of the nature of risk factors due to QT interval prolonging drug use and potentially inappropriate psychotropic use in elderly psychiatry outpatients. Ther. Adv. Cardiovasc. Dis..

[20] Roca B., Roca M. (2022). Assessment of Drug Interactions with Online Electronic Checkers in Multi-Pathological Patients. Pharmacology.

[21] Şen S., Karahan E., Büyükulaş C., Polat Y.O., Üresin A.Y. (2021). Colchicine for cardiovascular therapy: A drug interaction perspective and a safety meta-analysis. Anatol. J. Cardiol..

[22] Assefa Y.A., Kedir A., Kahaliw W. (2020). Survey on Polypharmacy and Drug-Drug Interactions among Elderly People with Cardiovascular Diseases at Yekatit 12 Hospital, Addis Ababa, Ethiopia. Integr. Pharm. Res. Pract..

[23] Jain S., Jain P., Sharma K., Saraswat P. (2017). A Prospective Analysis of Drug Interactions in Patients of Intensive Cardiac Care Unit. J. Clin. Diagn. Res..

[24] Yao X., Tsang T., Sun Q., Quinney S., Zhang P., Ning X., Li L., Shen L. (2020). Mining and visualizing high-order directional drug interaction effects using the FAERS database. BMC Med. Inform. Decis. Mak..

[25] Alrowais F., Alotaibi S.S., Hilal A.M., Marzouk R., Mohsen H., Osman A.E., Alneil A.A., Eldesouki M.I. (2023). Clinical Decision Support Systems to Predict Drug-Drug Interaction Using Multilabel Long Short-Term Memory with an Autoencoder. Int. J. Environ. Res. Public Health.

[26] Yu L., Xu Z., Cheng M., Lin W., Qiu W., Xiao X. (2023). MSEDDI: Multi-Scale Embedding for Predicting Drug-Drug Interaction Events. Int. J. Mol. Sci..

[27] Brattig Correia R., de Araújo Kohler L.P., Mattos M.M., Rocha L.M. (2019). City-wide electronic health records reveal gender and age biases in administration of known drug-drug interactions. NPJ Digit. Med..

[28] Spanakis M., Ioannou P., Tzalis S., Papakosta V., Patelarou E., Tzanakis N., Patelarou A., Kofteridis D.P. (2022). Drug-Drug Interactions among Patients Hospitalized with COVID-19 in Greece. J. Clin. Med..

[29] Ingersgaard M.V., Helms Andersen T., Norgaard O., Grabowski D., Olesen K. (2020). Reasons for Nonadherence to Statins—A Systematic Review of Reviews. Patient Prefer. Adherence.

[30] Rochon P.A., Petrovic M., Cherubini A., Onder G., O’Mahony D., Sternberg S.A., Stall N.M., Gurwitz J.H. (2021). Polypharmacy, inappropriate prescribing, and deprescribing in older people: Through a sex and gender lens. Lancet Healthy Longev..

[31] Chazova I.E., Zhernakova Y.V. (2019). Diagnosis and treatment of arterial hypertension [Guidelines]. Syst. Hypertens..

[32] Dumbreck S., Flynn A., Nairn M., Wilson M., Treweek S., Mercer S.W., Alderson P., Thompson A., Payne K., Guthrie B. (2015). Drug-disease and drug-drug interactions: Systematic examination of recommendations in 12 UK national clinical guidelines. BMJ.

[33] Tan Y.Y., Papez V., Chang W.H., Mueller S.H., Denaxas S., Lai A.G. (2022). Comparing clinical trial population representativeness to real-world populations: An external validity analysis encompassing 43,895 trials and 5,685,738 individuals across 989 unique drugs and 286 conditions in England. Lancet Healthy Longev..

[34] Tamargo J., Kjeldsen K.P., Delpón E., Semb A.G., Cerbai E., Dobrev D., Savarese G., Sulzgruber P., Rosano G., Borghi C. (2022). Facing the challenge of polypharmacy when prescribing for older people with cardiovascular disease. A review by the European Society of Cardiology Working Group on Cardiovascular Pharmacotherapy. Eur. Heart J. Cardiovasc. Pharmacother..

[35] Anfinogenova N.D., Trubacheva I.A., Popov S.V., Efimova E.V., Ussov W.Y. (2021). Trends and concerns of potentially inappropriate medication use in patients with cardiovascular diseases. Expert. Opin. Drug Saf..

[36] Anfinogenova Y., Grakova E.V., Shvedova M., Kopieva K.V., Teplyakov A.T., Popov S.V. (2018). Interdisciplinary approach to compensation of hypoglycemia in diabetic patients with chronic heart failure. Heart Fail. Rev..

[37] Lyles C.R., Nelson E.C., Frampton S., Dykes P.C., Cemballi A.G., Sarkar U. (2020). Using Electronic Health Record Portals to Improve Patient Engagement: Research Priorities and Best Practices. Ann. Intern. Med..

