# Supplementary material for: Prescribed Versus Taken Polypharmacy and Drug–Drug Interactions in Older Cardiovascular Patients during the COVID-19 Pandemic: Observational Cross-Sectional Analytical Study
Source: J Clin Med. 2023 Aug 1;12(15):5061. doi: 10.3390/jcm12155061 (PMC10420276; doi:10.3390/jcm12155061)
Supplement: Supplementary file 1 [file jcm-12-05061-s001.zip › jcm-2500084-supplementary.pdf]

Journal of
Clinical Medicine

MDPI

Table S1. Codes of International Statistical Classification of Diseases and Related Health Problems in the study cohort according to data of electronic health records in 2019-2022 (*n* = 704).

ICD	Clinical condition	n*
A46	Erysipelas	1
A84.9	Tick-borne viral encephalitis	2
C18.8	Malignant neoplasm: Overlapping lesion of colon	1
C44.3	Other malignant neoplasms: Skin of other and unspecified parts of face	1
C50.3	Malignant neoplasm: Lower-inner quadrant of breast	1
C50.9	Malignant neoplasm: Breast, unspecified	4
C61	Malignant neoplasm of prostate	3
C67.8	Malignant neoplasm: Overlapping lesion of bladder	4
D12.0	Benign neoplasm of cecum	1
D24	Benign neoplasm of breast	1
D40.0	Neoplasm of uncertain or unknown behaviour: Prostate	1
D50.9	Iron deficiency anemia, unspecified	7
D64.8	Other specified anemias	1
D64.9	Anemia, unspecified	4
D69.5	Secondary thrombocytopenia	1
E01.8	Other iodine-deficiency-related thyroid disorders and allied conditions	1
E04.1	Nontoxic single thyroid nodule	2
E04.2	Nontoxic multinodular goiter	3
E05.9	Thyrotoxicosis, unspecified	1
E06.3	Autoimmune thyroiditis	8
E06.5	Other chronic thyroiditis	2
E10.7	Insulin-dependent diabetes mellitus: With multiple complications	1
E11.3	Type 2 diabetes mellitus With ophthalmic complications	1
E11.4	Type 2 diabetes mellitus With neurological complications	1
E11.6	Type 2 diabetes mellitus With other specified	1

ICD	Clinical condition	n*
I70.2	Atherosclerosis of native arteries of the extremities	20
I70.8	Atherosclerosis of other arteries	2
I74.3	Embolism and thrombosis of arteries of the lower extremities	1
I83.9	Asymptomatic varicose veins of lower extremities	4
I95.8	Other hypotension	1
J00	Acute nasopharyngitis [common cold]	1
J02.9	Acute pharyngitis, unspecified	1
J06.8	Other acute upper respiratory infections of multiple sites	10
J06.9	Acute upper respiratory infection, unspecified	15
J12.8	Other viral pneumonia	11
J18.9	Pneumonia, unspecified organism	9
J20.9	Acute bronchitis, unspecified	1
J31.1	Chronic nasopharyngitis	1
J40	Bronchitis, not specified as acute or chronic	1
J41.0	Simple chronic bronchitis	2
J42	Unspecified chronic bronchitis	2
J44.8	Other specified chronic obstructive pulmonary disease	4
J44.9	Chronic obstructive pulmonary disease	4
J45.8	Mixed asthma	7
K20	Esophagitis	1
K21.0	Gastro-oesophageal reflux disease with oesophagitis	3
K21.9	Gastro-oesophageal reflux disease without oesophagitis	1
K26.7	Chronic duodenal ulcer without hemorrhage or perforation	2
K29.3	Chronic superficial gastritis	3
K29.4	Chronic atrophic gastritis	1

	complications	
E11.7	Type 2 diabetes mellitus With multiple complications	32
E11.8	Type 2 diabetes mellitus with unspecified complications	6
E11.9	Type 2 diabetes mellitus without complications	7
E16.8	Other specified disorders of pancreatic internal secretion	6
E66.0	Obesity due to excess calories	2
E66.8	Other obesity	3
E78.0	Pure hypercholesterolemia	1
E78.8	Other disorders of lipoprotein metabolism	2
E79.0	Hyperuricemia without signs of inflammatory arthritis and tophaceous disease	2
F06.4	Anxiety disorder due to known physiological condition	1
F43.2	Adjustment disorders	1
G20	Parkinson's disease	1
G44.2	Cluster headache syndrome	1
G50.0	Trigeminal neuralgia	1
G52.8	Paralytic syndrome, unspecified	1
G56.0	Carpal tunnel syndrome	1
G62.8	Other specified polyneuropathies	1
G63.2	Diabetic polyneuropathy	2
G93.8	Other specified disorders of brain	2
H20.8	Other iridocyclitis	1
H25.0	Age-related incipient cataract	1
H25.1	Age-related nuclear cataract	1
H25.8	Other age-related cataract	7
H26.2	Complicated Cataract	2
H35.0	Background retinopathy and retinal vascular changes	1
H35.3	Degeneration of macula and posterior pole	1
H40.1	Primary open-angle glaucoma	1
H40.5	Glaucoma secondary to other eye disorders	1
H40.8	Other glaucoma	1
H43.1	Vitreous hemorrhage	1

K29.5	Chronic gastritis, unspecified	1
K29.7	Gastritis, unspecified	2
K29.9	Gastroduodenitis, unspecified	9
K31.7	Polyp of stomach and duodenum	2
K31.9	Disease of stomach and duodenum, unspecified	5
K56.5	Intestinal adhesions [bands] with obstruction (postinfection)	1
K58.9	Irritable bowel syndrome without diarrhea	1
K59.0	Constipation	2
K62.0	Anal polyp	1
K64.1	Second degree hemorrhoids	1
K64.8	Other hemorrhoids	2
K76.0	Fatty (change of) liver, not elsewhere classified	3
K80.1	Calculus of gallbladder with other cholecystitis	4
K80.2	Calculus of gallbladder without cholecystitis	1
K80.3	Calculus of bile duct with cholangitis	1
K80.8	Other cholelithiasis	1
K81.1	Chronic cholecystitis	2
K81.9	Cholecystitis, unspecified	1
K83.1	Obstruction of bile duct	1
K85.0	Idiopathic acute pancreatitis	1
K85.1	Biliary acute pancreatitis	1
K85.9	Acute pancreatitis, unspecified	1
K86.1	Other chronic pancreatitis	5
K91.5	Postcholecystectomy syndrome	2
L30.9	Dermatitis, unspecified	1
L71.9	Rosacea, unspecified	2
M10.0	Idiopathic gout	1
M15.0	Primary generalized (osteo)arthritis	4
M15.8	Other polyosteoarthritis	2
M15.9	Polyosteoarthritis, unspecified	1

H43.3	Vitreous prolapse, bilateral	2	M17.0	Primary gonarthrosis, bilateral	4
H47.2	Optic atrophy	2	M17.2	Bilateral post-traumatic osteoarthritis of knee	1
H52.1	Myopia	2	M17.3	Unilateral post-traumatic osteoarthritis of knee	1
H60.5	Acute otitis externa, noninfective	1	M17.9	Osteoarthritis of knee, unspecified	2
H60.9	Unspecified otitis externa	1	M19.8	Other specified arthrosis	3
H90.3	Sensorineural hearing loss, bilateral	3	M42.1	Adult osteochondrosis of spine	10
I05.0	Rheumatic mitral stenosis	1	M42.9	Spinal osteochondrosis, unspecified	1
I05.1	Rheumatic mitral insufficiency	1	M50.3	Other cervical disc degeneration	1
I08.0	Rheumatic disorders of both mitral and aortic valves	1	M53.0	Cervicocranial syndrome	2
I10	Essential (primary) hypertension	4	M53.1	Cervicobrachial syndrome	2
I11	Hypertensive heart disease with heart failure	1	M54.2	Cervicalgia	3
I11.0	Hypertensive heart disease with (congestive) heart failure	5	M54.4	Lumbago with sciatica	4
I11.9	Hypertensive heart disease without (congestive) heart failure	108	M54.5	Low Back Pain	1
I12.0	Hypertensive chronic kidney disease with stage 5 chronic kidney disease or end stage renal disease	2	M54.6	Pain in thoracic spine	1
I13.2	Hypertensive heart and renal disease with both (congestive) heart failure and renal failure	1	M65.9	Synovitis and tenosynovitis, unspecified	1
I20.0	Unstable angina pectoris	5	M79.2	Neuralgia and neuritis, unspecified	1
I20.1	Angina pectoris with documented spasm	2	M81.8	Other osteoporosis without current pathological fracture	1
I20.8	Other forms of angina pectoris	153	N10	Acute pyelonephritis	1
I20.9	Angina pectoris, unspecified	2	N11.0	Nonobstructive reflux-associated chronic pyelonephritis	3
I21.0	Acute transmural myocardial infarction of anterior wall	9	N11.8	Other chronic tubulo-interstitial nephritis	4
I21.1	Acute transmural myocardial infarction of inferior wall	2	N11.9	Chronic tubulo-interstitial nephritis, unspecified	1
I21.9	ST elevation (STEMI) myocardial infarction involving other coronary artery of anterior wall	1	N18.2	Chronic kidney disease, stage 2 (mild)	8
I22.0	Subsequent ST elevation (STEMI) myocardial infarction of anterior wall	1	N18.3	Chronic kidney disease, stage 3 (moderate)	5
I22.1	Subsequent myocardial infarction of inferior wall	2	N18.4	Chronic kidney disease, stage 4 (severe)	1
I22.9	ST elevation (STEMI) myocardial infarction of unspecified site	1	N18.5	Chronic kidney disease, stage 5	1

I25.0	Chronic ischaemic heart disease, unspecified	4	N18.9	Chronic kidney disease, unspecified	1
I25.1	Atherosclerotic heart disease of native coronary artery	11	N20.9	Urinary calculus, unspecified	1
I25.2	Old myocardial infarction	39	N28.1	Cyst of kidney, acquired	1
I25.8	Other forms of chronic ischemic heart disease	27	N30.0	Acute cystitis	1
I25.9	Chronic ischaemic heart disease, unspecified	9	N30.1	Interstitial cystitis (chronic)	1
I35.2	Aortic (valve) stenosis with insufficiency	1	N30.2	Other chronic cystitis	1
I42.0	Dilated cardiomyopathy	1	N30.8	Other cystitis	1
I42.8	Other cardiomyopathies	1	N30.9	Cystitis, unspecified	2
I44.0	Atrioventricular block, first degree	6	N39.4	Other specified urinary incontinence	1
I44.1	Atrioventricular block, second degree	2	N40	Benign prostatic hyperplasia	12
I44.2	Atrioventricular block	1	N41.3	Prostatocystitis	1
I44.6	Other and unspecified fascicular block	1	N60.8	Other benign mammary dysplasias	1
I47.1	Supraventricular (paroxysmal) tachycardia	1	R16.0	Hepatomegaly, not elsewhere classified	1
I47.2	Ventricular tachycardia	2	R31	Unspecified haematuria	2
I48.0	Paroxysmal atrial fibrillation	15	R51	Headache	1
I48.1	Persistent atrial fibrillation	4	R52.9	Pain, unspecified	1
I48.2	Chronic atrial fibrillation	2	R63.5	Abnormal weight gain	1
I48.9	Atrial fibrillation and atrial flutter, unspecified	5	R73.9	Hyperglycemia, unspecified	2
I49.3	Ventricular premature depolarization	1	S00.0	Superficial injury of scalp	1
I49.5	Sick sinus syndrome	3	U07.1	COVID-19, virus identified	11
I49.8	Cardiac arrhythmia, unspecified	1	U07.2	COVID-19, virus not identified	1
I50.0	Congestive heart failure	5	U11.9	Need for immunization against COVID-19, unspecified	1
I50.1	Left ventricular failure, unspecified	1	Z00.0	Encounter for general adult medical examination	2
I50.9	Heart failure, unspecified	2	Z00.8	Encounter for other general examination	2
I51	Complications and ill-defined descriptions of heart disease	1	Z01.8	Encounter for other specified special examinations	2
I63.5	Cerebral infarction due to unspecified occlusion or stenosis of cerebral arteries	5	Z03.1	Observation for suspected malignant neoplasm	1
I67.2	Cerebral atherosclerosis	1	Z03.8	Encounter for observation for other suspected diseases and conditions ruled out	4
I67.4	Hypertensive encephalopathy	1	Z25.1	Need for immunization against influenza	1
I67.8	Other specified cerebrovascular diseases	55	Z76.0	Encounter for issue of repeat prescription	3
I67.9	Cerebrovascular disease, unspecified	9	Z96.1	Presence of intraocular lens	4
I70.0	Atherosclerosis of aorta	3			

*n — number of patients with given ICD code in electronic health records in 2019-2022.

Table S2. The detailed explanation of the entire list of pairwise serious drug-drug interactions among taken drugs in the cohort of cardiovascular patients based on data of electronic health records in 2019-2022 (*n* = 704).

Drug combination	n	Serious drug-drug interactions (T-List)
aspirin + perindopril	25	Aspirin, perindopril. Pharmacodynamic antagonism. Avoid or Use Alternate Drug. Co-administration may result in a significant decrease in renal function. NSAIDs may diminish the antihypertensive effect of ACE inhibitors. The mechanism of these interactions is likely related to the ability of NSAIDs to reduce the synthesis of vasodilating renal prostaglandins
aspirin + lisinopril	17	Aspirin, lisinopril. Pharmacodynamic antagonism. Avoid or Use Alternate Drug. Co-administration may result in a significant decrease in renal function. NSAIDs may diminish the antihypertensive effect of ACE inhibitors. The mechanism of these interactions is likely related to the ability of NSAIDs to reduce the synthesis of vasodilating renal prostaglandins
amiodarone + indapamide	9	Amiodarone and indapamide both increase QTc interval. Avoid or Use Alternate Drug
aspirin + enalapril*	7	Aspirin, enalapril. pharmacodynamic antagonism. Avoid or Use Alternate Drug. Co-administration may result in a significant decrease in renal function. NSAIDs may diminish the antihypertensive effect of ACE inhibitors. The mechanism of these interactions is likely related to the ability of NSAIDs to reduce the synthesis of vasodilating renal prostaglandins
bisoprolol + digoxin	7	Bisoprolol increases effects of digoxin by pharmacodynamic synergism. Use Caution/Monitor. Enhanced bradycardia
clopidogrel + omeprazole	7	Omeprazole decreases effects of clopidogrel by affecting hepatic enzyme CYP2C19 metabolism. Avoid or Use Alternate Drug. Clopidogrel efficacy may be reduced by drugs that inhibit CYP2C19. Inhibition of platelet aggregation by clopidogrel is entirely due to an active metabolite. Clopidogrel is metabolized to this active metabolite in part by CYP2C19
ceftriaxone + enoxaparin	6	Ceftriaxone increases effects of enoxaparin by anticoagulation. Avoid or Use Alternate Drug. cephalosporins may decrease prothrombin activity
apixaban + clopidogrel	4	Clopidogrel and apixaban both increase anticoagulation. Avoid or Use Alternate Drug
digoxin + omeprazole	4	Esomeprazole will increase the level or effect of digoxin by increasing gastric pH. Applies only to oral form of both agents. Avoid or Use Alternate Drug
aspirin + ketorolac	3	Aspirin, ketorolac. Either increases toxicity of the other by pharmacodynamic synergism. Contraindicated
potassium + spironolactone	3	Spironolactone and potassium chloride both increase serum potassium. Avoid or Use Alternate Drug
amiodarone + digoxin	2	Amiodarone will increase the level or effect of digoxin by P-glycoprotein (MDR1) efflux transporter. Avoid or Use Alternate Drug. Amiodarone increases PO digoxin serum concentrations by ~70% and IV digoxin by ~17%; measure digoxin levels before initiating amiodarone and reduce PO digoxin dose by 30-50%; decrease IV digoxin dose by 15-30%. amiodarone will increase the level or effect of digoxin by basic (cationic) drug competition for renal

		tubular clearance. Avoid or Use Alternate Drug. Amiodarone increases PO digoxin serum concentrations by ~70% and IV digoxin by ~17%; measure digoxin levels before initiating amiodarone and reduce PO digoxin dose by 30-50%; decrease IV digoxin dose by 15-30%
aspirin + fosinopril	2	Aspirin, fosinopril. Pharmacodynamic antagonism. Avoid or Use Alternate Drug. Co-administration may result in a significant decrease in renal function. NSAIDs may diminish the antihypertensive effect of ACE inhibitors. The mechanism of these interactions is likely related to the ability of NSAIDs to reduce the synthesis of vasodilating renal prostaglandins
captopril + losartan	2	Losartan, captopril. Either increases toxicity of the other by pharmacodynamic synergism. Avoid or Use Alternate Drug. Dual blockade of renin-angiotensin system increases risks of hypotension, hyperkalemia, and renal impairment
ceftriaxone + heparin	2	Ceftriaxone will increase the level or effect of heparin by anticoagulation. Avoid or Use Alternate Drug. cephalosporins may decrease prothrombin activity
clarithromycin + enoxaparin	2	Clarithromycin increases effects of enoxaparin by decreasing metabolism. Avoid or Use Alternate Drug
digoxin + metoprolol	2	Digoxin increases toxicity of metoprolol by unspecified interaction mechanism. Avoid or Use Alternate Drug. Can increase risk of bradycardia
ketorolac + lisinopril	2	Ketorolac, lisinopril. pharmacodynamic antagonism. Avoid or Use Alternate Drug. Co-administration may result in a significant decrease in renal function. NSAIDs may diminish the antihypertensive effect of ACE inhibitors. The mechanism of these interactions is likely related to the ability of NSAIDs to reduce the synthesis of vasodilating renal prostaglandins
metoprolol + sotalol	2	Metoprolol and sotalol both increase anti-hypertensive channel blocking. Avoid or Use Alternate Drug
amiodarone + escitalopram	1	Escitalopram increases toxicity of amiodarone by QTc interval. Avoid or Use Alternate Drug
amiodarone + haloperidol	1	Amiodarone and haloperidol both increase QTc interval. Avoid or Use Alternate Drug
amiodarone + levofloxacin	1	Amiodarone and levofloxacin both increase QTc interval. Avoid or Use Alternate Drug
apixaban + enoxaparin	1	Enoxaparin and apixaban both increase anticoagulation. Avoid or Use Alternate Drug
apixaban + rivaroxaban	1	Rivaroxaban and apixaban both increase anticoagulation. Avoid or Use Alternate Drug
atenolol + sotalol	1	Atenolol and sotalol both increase anti-hypertensive channel blocking. Avoid or Use Alternate Drug
azithromycin + heparin	1	Azithromycin increases effects of heparin by decreasing metabolism. Avoid or Use Alternate Drug
bisoprolol + metoprolol	1	Bisoprolol and metoprolol both increase anti-hypertensive channel blocking. Avoid or Use Alternate Drug

clarithromycin + dexamethasone	1	Clarithromycin will increase the level or effect of dexamethasone by affecting hepatic/intestinal enzyme CYP3A4 metabolism. Avoid or Use Alternate Drug
clopidogrel + morphine	1	Morphine will decrease the level or effect of clopidogrel by Other (see comment). Avoid or Use Alternate Drug. Co-administration of opioid agonists delay and reduce absorption of clopidogrel, presumably because of slowed gastric emptying, resulting in reduced exposure to its metabolites; consider use of parenteral antiplatelet agents in acute coronary syndrome patients requiring co-administration of morphine or other opioid agonists
dalteparin + enoxaparin	1	Dalteparin and enoxaparin both increase anticoagulation. Avoid or Use Alternate Drug
diclofenac + enalapril	1	Diclofenac, enalapril. pharmacodynamic antagonism. Avoid or Use Alternate Drug. Co-administration may result in a significant decrease in renal function. NSAIDs may diminish the antihypertensive effect of ACE inhibitors. The mechanism of these interactions is likely related to the ability of NSAIDs to reduce the synthesis of vasodilating renal prostaglandins
diclofenac + ketorolac	1	Diclofenac, ketorolac. Either increases toxicity of the other by pharmacodynamic synergism. Contraindicated
diclofenac + lisinopril	1	Diclofenac, lisinopril. pharmacodynamic antagonism. Avoid or Use Alternate Drug. Co-administration may result in a significant decrease in renal function. NSAIDs may diminish the antihypertensive effect of ACE inhibitors. The mechanism of these interactions is likely related to the ability of NSAIDs to reduce the synthesis of vasodilating renal prostaglandins
enalapril + ketorolac	1	Ketorolac, enalapril. pharmacodynamic antagonism. Avoid or Use Alternate Drug. Co-administration may result in a significant decrease in renal function. NSAIDs may diminish the antihypertensive effect of ACE inhibitors. The mechanism of these interactions is likely related to the ability of NSAIDs to reduce the synthesis of vasodilating renal prostaglandins
enalapril + losartan	1	Losartan, enalapril. Either increases toxicity of the other by pharmacodynamic synergism. Avoid or Use Alternate Drug. Dual blockade of renin-angiotensin system increases risks of hypotension, hyperkalemia, and renal impairment
enoxaparin + heparin	1	Enoxaparin and heparin both increase anticoagulation. Avoid or Use Alternate Drug
fluconazole + octreotide	1	Fluconazole and octreotide both increase QTc interval. Avoid or Use Alternate Drug
heparin + warfarin	1	Heparin increases effects of warfarin by anticoagulation. Avoid or Use Alternate Drug. Avoid combined use once INR is established in the desired therapeutic range
ketorolac + meloxicam	1	Meloxicam, ketorolac. Either increases toxicity of the other by pharmacodynamic synergism. Contraindicated
lisinopril + losartan	1	Losartan, lisinopril. Either increases toxicity of the other by pharmacodynamic synergism. Avoid or Use Alternate Drug. Dual blockade of renin-angiotensin system increases risks of hypotension, hyperkalemia, and renal impairment
lisinopril + meloxicam	1	Meloxicam, lisinopril. pharmacodynamic antagonism. Avoid or Use Alternate

	Drug. Co-administration may result in a significant decrease in renal function. NSAIDs may diminish the antihypertensive effect of ACE inhibitors. The mechanism of these interactions is likely related to the ability of NSAIDs to reduce the synthesis of vasodilating renal prostaglandins

***Note:** Impact of drug-drug interactions associated with the combinations 'aspirin + captopril' and 'aspirin + enalapril' may be considered insignificant due to the use of low-dose aspirin in the majority of cases. Administration of aspirin at doses less than 300 mg per day has little effect on the effectiveness of captopril and enalapril. Administration of aspirin in higher doses reduces the effectiveness of captopril and enalapril.

Table S3. The detailed explanation of the entire list of pairwise monitor-closely drug-drug interactions among taken drugs in the cohort of cardiovascular patients based on data of electronic health records in 2019-2022 (*n* = 704).

Drug combination	n	Monitor-closely drug-drug interactions (T-List)
aspirin + losartan	104	Aspirin decreases effects of losartan by pharmacodynamic antagonism. Modify Therapy/Monitor Closely. NSAIDs decrease synthesis of vasodilating renal prostaglandins, and thus affect fluid homeostasis and may diminish antihypertensive effect
aspirin + bisoprolol	94	Aspirin decreases effects of bisoprolol by pharmacodynamic antagonism. Use Caution/Monitor. Long term (>1 wk) NSAID use. NSAIDs decrease prostaglandin synthesis
bisoprolol + losartan	58	Bisoprolol, losartan. Mechanism: pharmacodynamic synergism. Use Caution/Monitor. Risk of fetal compromise if given during pregnancy
aspirin + spironolactone	42	Aspirin decreases effects of spironolactone by unspecified interaction mechanism. Use Caution/Monitor. When used concomitantly, spironolactone dose may need to be titrated to higher maintenance dose and the patient should be observed closely to determine if the desired effect is obtained
amiodarone + losartan	32	Amiodarone will increase the level or effect of losartan by affecting hepatic enzyme CYP2C9/10 metabolism. Use Caution/Monitor. May inhibit the conversion of losartan to its active metabolite E-3174. Importance of interaction not established; monitor individual therapeutic response to determine losartan dosage
aspirin + metoprolol	32	Aspirin decreases effects of metoprolol by pharmacodynamic antagonism. Use Caution/Monitor. Long term (>1 wk) NSAID use. NSAIDs decrease prostaglandin synthesis
losartan + metoprolol	32	Losartan and metoprolol both increase serum potassium. Use Caution/Monitor
bisoprolol + torsemide	31	Bisoprolol increases and torsemide decreases serum potassium. Effect of interaction is not clear, use caution. Use Caution/Monitor
digoxin + spironolactone	30	Spironolactone increases levels of digoxin by Other (see comment). Use Caution/Monitor. Comment: Spironolactone may cause false elevation of digoxin assay
aspirin + perindopril	25	Aspirin, perindopril. pharmacodynamic antagonism. Avoid or Use Alternate Drug. Co-administration may result in a significant decrease in renal function. NSAIDs may diminish the antihypertensive effect of ACE inhibitors. The mechanism of these interactions is likely related to the ability of NSAIDs to reduce the synthesis of vasodilating renal prostaglandins
aspirin + torsemide	24	Aspirin increases and torsemide decreases serum potassium. Effect of interaction is not clear, use caution. Use Caution/Monitor
spironolactone + torsemide	24	Spironolactone increases and torsemide decreases serum potassium. Effect of interaction is not clear, use caution. Modify Therapy/Monitor Closely
aspirin + candesartan	21	Aspirin decreases effects of candesartan by pharmacodynamic antagonism. Modify Therapy/Monitor Closely. NSAIDs decrease synthesis of vasodilating

		renal prostaglandins, and thus affect fluid homeostasis and may diminish antihypertensive effect
amlodipine + bisoprolol	20	Bisoprolol, amlodipine. Either increases effects of the other by pharmacodynamic synergism. Use Caution/Monitor. Both drugs lower blood pressure
aspirin + clopidogrel	20	Aspirin, clopidogrel. Either increases toxicity of the other by pharmacodynamic synergism. Use Caution/Monitor. The need for simultaneous use of low-dose aspirin and anticoagulant or antiplatelet agents are common for patients with cardiovascular disease; monitor closely
digoxin + torsemide	20	Digoxin increases and torsemide decreases serum potassium. Effect of interaction is not clear, use caution. Use Caution/Monitor
losartan + torsemide	20	Losartan increases and torsemide decreases serum potassium. Effect of interaction is not clear, use caution. Use Caution/Monitor
bisoprolol + spironolactone	19	Bisoprolol and spironolactone both increase serum potassium. Modify Therapy/Monitor Closely
insulin aspart + lisinopril	19	Lisinopril increases effects of insulin aspart by pharmacodynamic synergism. Use Caution/Monitor
aspirin + insulin	18	Aspirin increases effects of insulin aspart by pharmacodynamic synergism. Modify Therapy/Monitor Closely. Co-administration of insulin with high doses of salicylates (3 g/day or more) may increase risk for hypoglycemia. Insulin dose adjustment and increased frequency of glucose monitoring may be required
aspirin + lisinopril	18	Aspirin, lisinopril. pharmacodynamic antagonism. Avoid or Use Alternate Drug. Co-administration may result in a significant decrease in renal function. NSAIDs may diminish the antihypertensive effect of ACE inhibitors. The mechanism of these interactions is likely related to the ability of NSAIDs to reduce the synthesis of vasodilating renal prostaglandins
bisoprolol + digoxin	18	Bisoprolol increases effects of digoxin by pharmacodynamic synergism. Use Caution/Monitor. Enhanced bradycardia
furosemide + spironolactone	18	Spironolactone increases and furosemide decreases serum potassium. Effect of interaction is not clear, use caution. Modify Therapy/Monitor Closely
indapamide + losartan	18	Losartan increases and indapamide decreases serum potassium. Effect of interaction is not clear, use caution. Use Caution/Monitor
losartan + spironolactone	18	Losartan and spironolactone both increase serum potassium. Modify Therapy/Monitor Closely
lisinopril + spironolactone	16	Lisinopril, spironolactone. Mechanism: pharmacodynamic synergism. Use Caution/Monitor. Risk of hyperkalemia
amiodarone + atorvastatin	14	Amiodarone will increase the level or effect of atorvastatin by P-glycoprotein (MDR1) efflux transporter. Use Caution/Monitor
aspirin + carvedilol	14	Aspirin decreases effects of carvedilol by pharmacodynamic antagonism. Use Caution/Monitor. Long term (>1 wk) NSAID use. NSAIDs decrease prostaglandin synthesis
aspirin + enoxaparin	14	Aspirin, enoxaparin. Either increases toxicity of the other by pharmacody-

		namic synergism. Use Caution/Monitor. The need for simultaneous use of low-dose aspirin and anticoagulant or antiplatelet agents are common for patients with cardiovascular disease; monitor closely
atorvastatin + dexamethasone	14	Atorvastatin will increase the level or effect of dexamethasone by P-glycoprotein (MDR1) efflux transporter. Use Caution/Monitor
candesartan + metoprolol	14	Candesartan and metoprolol both increase serum potassium. Use Caution/Monitor
sotalol + spironolactone	13	Sotalol and spironolactone both increase serum potassium. Modify Therapy/Monitor Closely
aspirin + furosemide	12	Aspirin increases and furosemide decreases serum potassium. Effect of interaction is not clear, use caution. Use Caution/Monitor
aspirin + heparin	12	Aspirin, heparin. Either increases toxicity of the other by anticoagulation. Use Caution/Monitor. The need for simultaneous use of low-dose aspirin and anticoagulant or antiplatelet agents are common for patients with cardiovascular disease; monitor closely
aspirin + valsartan	12	Aspirin decreases effects of valsartan by pharmacodynamic antagonism. Modify Therapy/Monitor Closely. NSAIDs decrease synthesis of vasodilating renal prostaglandins, and thus affect fluid homeostasis and may diminish antihypertensive effect
atorvastatin + ranolazine	12	Ranolazine will increase the level or effect of atorvastatin by P-glycoprotein (MDR1) efflux transporter. Use Caution/Monitor
metformin + torsemide	12	Torsemide decreases effects of metformin by pharmacodynamic antagonism. Use Caution/Monitor
amiodarone + rivaroxaban	11	Amiodarone increases levels of rivaroxaban by affecting hepatic/intestinal enzyme CYP3A4 metabolism. Use Caution/Monitor. Patients with renal impairment receiving rivaroxaban with drugs that are combined P-gp and weak or moderate CYP3A4 inhibitors may have significant increases in exposure compared with patients with normal renal function and no inhibitor use, since both pathways of rivaroxaban elimination are affected. Since these increases may increase bleeding risk, use rivaroxaban in this situation only if the potential benefit justifies the potential risk
amiodarone + metoprolol	10	Amiodarone will increase the level or effect of metoprolol by affecting hepatic enzyme CYP2D6 metabolism. Use Caution/Monitor. Monitor cardiac function carefully and observe for signs of bradycardia or heart block when amiodarone and a beta adrenergic blocker are co-administered. Amiodarone should be used with caution in patients receiving a beta adrenergic blocker, particularly if there is suspicion of underlying dysfunction of the sinus node, such as bradycardia or sick sinus syndrome, or if there is partial AV block
aspirin + indapamide	10	Aspirin increases and indapamide decreases serum potassium. Effect of interaction is not clear, use caution. Use Caution/Monitor
atorvastatin + valsartan	10	Atorvastatin will increase the level or effect of valsartan by Other (see comment). Use Caution/Monitor. The results from an in vitro study with human liver tissue indicate that valsartan is a substrate of the hepatic uptake transporter OATP1B1; co-administration with OATP1B1 inhibitors may increase

		valsartan systemic exposure
digoxin + losartan	10	Losartan and digoxin both increase serum potassium. Use Caution/Monitor
hydrochlorothiazide + metoprolol	10	Hydrochlorothiazide, metoprolol. Either increases toxicity of the other by Other (see comment). Modify Therapy/Monitor Closely. Comment: May cause idiosyncratic reaction, resulting in acute transient myopia and acute angle-closure glaucoma, which can lead to permanent vision loss
aspirin + hydrochlorothiazide	9	Aspirin increases and hydrochlorothiazide decreases serum potassium. Effect of interaction is not clear, use caution. Use Caution/Monitor
furosemide + lisinopril	9	Lisinopril, furosemide. Mechanism: pharmacodynamic synergism. Use Caution/Monitor. Risk of acute hypotension, renal insufficiency
lisinopril + torsemide	9	Lisinopril, torsemide. Mechanism: pharmacodynamic synergism. Use Caution/Monitor. Risk of acute hypotension, renal insufficiency
losartan + omeprazole	9	Omeprazole will increase the level or effect of losartan by affecting hepatic enzyme CYP2C9/10 metabolism. Use Caution/Monitor. May inhibit the conversion of losartan to its active metabolite E-3174. Importance of interaction not established; monitor individual therapeutic response to determine losartan dosage
metoprolol + spironolactone	9	Metoprolol and spironolactone both increase serum potassium. Modify Therapy/Monitor Closely
amlodipine + carvedilol	8	Carvedilol and amlodipine both increase anti-hypertensive channel blocking. Modify Therapy/Monitor Closely
aspirin + rivaroxaban	8	Aspirin, rivaroxaban. Either increases toxicity of the other by anticoagulation. Use Caution/Monitor. Both drugs have the potential to cause bleeding. The need for simultaneous use of low-dose aspirin (<100 mg/day) with anticoagulants are common for patients with cardiovascular disease, but may result in increased bleeding; monitor closely. Promptly evaluate any signs or symptoms of blood loss if treated concomitantly with low-dose aspirin. Avoid co-administration with chronic use of higher dose aspirin
atorvastatin + nifedipine	8	Nifedipine will increase the level or effect of atorvastatin by affecting hepatic/intestinal enzyme CYP3A4 metabolism. Use Caution/Monitor. nifedipine will decrease the level or effect of atorvastatin by P-glycoprotein (MDR1) efflux transporter. Use Caution/Monitor
bisoprolol + furosemide	8	Bisoprolol increases and furosemide decreases serum potassium. Effect of interaction is not clear, use caution. Use Caution/Monitor
clopidogrel + rivaroxaban	8	Rivaroxaban, clopidogrel. Other (see comment). Use Caution/Monitor. Comment: Avoid concurrent administration of clopidogrel with rivaroxaban unless the benefit outweighs the risk of increased bleeding
furosemide + losartan	8	Losartan increases and furosemide decreases serum potassium. Effect of interaction is not clear, use caution. Use Caution/Monitor
lisinopril + metformin	8	Lisinopril increases toxicity of metformin by unspecified interaction mechanism. Use Caution/Monitor. Increases risk for hypoglycemia and lactic acidosis

amlodipine + metformin	7	Amlodipine decreases effects of metformin by pharmacodynamic antagonism. Use Caution/Monitor. Patient should be closely observed for loss of blood glucose control; when drugs are withdrawn from a patient receiving metformin, patient should be observed closely for hypoglycemia
aspirin + enalapril*	7	Aspirin, enalapril. pharmacodynamic antagonism. Avoid or Use Alternate Drug. Co-administration may result in a significant decrease in renal function. NSAIDs may diminish the antihypertensive effect of ACE inhibitors. The mechanism of these interactions is likely related to the ability of NSAIDs to reduce the synthesis of vasodilating renal prostaglandins
clopidogrel + verapamil	7	Verapamil will decrease the level or effect of clopidogrel by affecting hepatic/intestinal enzyme CYP3A4 metabolism. Use Caution/Monitor. Inhibition of CYP3A4 will reduce clopidogrel bioactivation
dexamethasone + enoxaparin	7	Dexamethasone, enoxaparin. Other (see comment). Use Caution/Monitor. Comment: Corticosteroids may decrease anticoagulant effects by increasing blood coagulability; conversely, they may impair vascular integrity, thus increasing bleeding risk. Monitor INR closely
enoxaparin + losartan	7	Enoxaparin increases toxicity of losartan by Other (see comment). Use Caution/Monitor. Comment: Low molecular weight heparins may suppress adrenal aldosterone secretion, which can potentially cause hyperkalemia
furosemide + sotalol	7	Sotalol increases and furosemide decreases serum potassium. Effect of interaction is not clear, use caution. Use Caution/Monitor
indapamide + metoprolol	7	Metoprolol increases and indapamide decreases serum potassium. Effect of interaction is not clear, use caution. Use Caution/Monitor
sotalol + torsemide	7	Sotalol increases and torsemide decreases serum potassium. Effect of interaction is not clear, use caution. Use Caution/Monitor
amlodipine + sotalol	6	Sotalol and amlodipine both increase anti-hypertensive channel blocking. Modify Therapy/Monitor Closely
apixaban + aspirin	6	Aspirin and apixaban both increase anticoagulation. Modify Therapy/Monitor Closely. Both drugs have the potential to cause bleeding. The need for simultaneous use of low-dose aspirin (<100 mg/day) with anticoagulants are common for patients with cardiovascular disease, but may result in increased bleeding; monitor closely. Promptly evaluate any signs or symptoms of blood loss if treated concomitantly with low-dose aspiriin. Avoid co-administration with chronic use of higher dose aspirin. In 1 trial (APPRAISE-2), therapy was terminated because of significantly increased bleeding when apixaban was administered with dual antiplatelet therapy (eg, aspirin plus clopidogrel) compared with single antiplatelet treatment
aspirin + azilsartan	6	Aspirin, azilsartan. Either increases toxicity of the other by Other (see comment). Use Caution/Monitor. Comment: May result in renal function deterioration, particularly in elderly or volume depleted individuals. aspirin decreases effects of azilsartan by pharmacodynamic antagonism. Modify Therapy/Monitor Closely. NSAIDs decrease synthesis of vasodilating renal prostaglandins, and thus affect fluid homeostasis and may diminish antihypertensive effect

aspirin + ketorolac	6	Aspirin, ketorolac. Either increases toxicity of the other by pharmacodynamic synergism. Contraindicated
aspirin + telmisartan	6	Aspirin decreases effects of telmisartan by pharmacodynamic antagonism. Modify Therapy/Monitor Closely. NSAIDs decrease synthesis of vasodilating renal prostaglandins, and thus affect fluid homeostasis and may diminish antihypertensive effect
bisoprolol + ketorolac	6	Bisoprolol and ketorolac both increase serum potassium. Use Caution/Monitor
candesartan + sotalol	6	Candesartan and sotalol both increase serum potassium. Use Caution/Monitor
digoxin + metformin	6	Digoxin, metformin. Either increases levels of the other by basic (cationic) drug competition for renal tubular clearance. Use Caution/Monitor. Measure serum digoxin concentrations before initiating metformin. Monitor patients who take both metformin and digoxin for possible digoxin toxicity and lactic acidosis. Reduce the digoxin and/or metformin dose as necessary
digoxin + omeprazole	6	Omeprazole will increase the level or effect of digoxin by increasing gastric pH. Applies only to oral form of both agents. Avoid or Use Alternate Drug
metformin + nifedipine	6	Nifedipine decreases effects of metformin by pharmacodynamic antagonism. Use Caution/Monitor. Patient should be closely observed for loss of blood glucose control; when drugs are withdrawn from a patient receiving metformin, patient should be observed closely for hypoglycemia. nifedipine increases levels of metformin by enhancing GI absorption. Applies only to oral form of both agents. Minor/Significance Unknown
amiodarone + bisoprolol	5	Amiodarone, bisoprolol. Mechanism: pharmacodynamic synergism. Use Caution/Monitor. Risk of cardiotoxicity with bradycardia
amiodarone + metformin	5	Amiodarone will increase the level or effect of metformin by basic (cationic) drug competition for renal tubular clearance. Use Caution/Monitor
aspirin + digoxin	5	Aspirin and digoxin both increase serum potassium. Use Caution/Monitor
atorvastatin + digoxin	5	Atorvastatin will increase the level or effect of digoxin by P-glycoprotein (MDR1) efflux transporter. Use Caution/Monitor
bisoprolol + valsartan	5	Bisoprolol, valsartan. Mechanism: pharmacodynamic synergism. Use Caution/Monitor. Risk of fetal compromise if given during pregnancy
candesartan + hydrochlorothiazide	5	Candesartan increases and hydrochlorothiazide decreases serum potassium. Effect of interaction is not clear, use caution. Use Caution/Monitor
carvedilol + nifedipine	5	Carvedilol and nifedipine both increase anti-hypertensive channel blocking. Modify Therapy/Monitor Closely
losartan + warfarin	5	Losartan will increase the level or effect of warfarin by affecting hepatic enzyme CYP2C9/10 metabolism. Use Caution/Monitor
perindopril + torsemide	5	Perindopril, torsemide. Mechanism: pharmacodynamic synergism. Use Caution/Monitor. Risk of acute hypotension, renal insufficiency
amiodarone + captopril	4	Amiodarone, captopril. Either increases effects of the other by pharmacodynamic synergism. Use Caution/Monitor. Both drugs lower blood pressure. Monitor blood pressure

amiodarone + warfarin	4	Amiodarone will increase the level or effect of warfarin by affecting hepatic enzyme CYP2C9/10 metabolism. Modify Therapy/Monitor Closely. Co-administration increases INR by 100% after 3-4 days. Reduce warfarin dose by one-third to one-half and monitor INR
aspirin + dexamethasone	4	Aspirin, dexamethasone. Either increases toxicity of the other by pharmacodynamic synergism. Use Caution/Monitor. Increased risk of GI ulceration
atorvastatin + budesonide	4	Atorvastatin will increase the level or effect of budesonide by P-glycoprotein (MDR1) efflux transporter. Use Caution/Monitor
bisoprolol + felodipine	4	Bisoprolol and felodipine both increase anti-hypertensive channel blocking. Modify Therapy/Monitor Closely
bisoprolol + hydrochlorothiazide	4	Bisoprolol increases and hydrochlorothiazide decreases serum potassium. Effect of interaction is not clear, use caution. Use Caution/Monitor
bisoprolol + indapamide	4	Bisoprolol increases and indapamide decreases serum potassium. Effect of interaction is not clear, use caution. Use Caution/Monitor
bisoprolol + potassium chloride	4	Bisoprolol and potassium chloride both increase serum potassium. Modify Therapy/Monitor Closely
bisoprolol + telmisartan	4	Bisoprolol, telmisartan. Mechanism: pharmacodynamic synergism. Use Caution/Monitor. Risk of fetal compromise if given during pregnancy
budesonide + omeprazole	4	Omeprazole decreases effects of budesonide by increasing gastric pH. Applies only to oral form of both agents. Modify Therapy/Monitor Closely. Enteric-coated budesonide dissolves at pH >5.5. Also, dissolution of extended-release budesonide tablets is pH dependent. Co-administration with drugs that increase gastric pH may cause these budesonide products to prematurely dissolve, and possibly affect release properties and absorption of the drug in the duodenum
clopidogrel + heparin	4	Heparin, clopidogrel. Either increases effects of the other by pharmacodynamic synergism. Modify Therapy/Monitor Closely. Enhanced risk of hemorrhage; additive effects are intended when both drugs are prescribed as indicated for ACS
digoxin + furosemide	4	Digoxin increases and furosemide decreases serum potassium. Effect of interaction is not clear, use caution. Use Caution/Monitor
digoxin + metoprolol	4	Digoxin increases toxicity of metoprolol by unspecified interaction mechanism. Avoid or Use Alternate Drug. Can increase risk of bradycardia
furosemide + torsemide	4	Furosemide and torsemide both decrease serum potassium. Use Caution/Monitor
metoprolol + torsemide	4	Metoprolol increases and torsemide decreases serum potassium. Effect of interaction is not clear, use caution. Use Caution/Monitor
omeprazole + warfarin	4	Omeprazole will increase the level or effect of warfarin by Other (see comment). Use Caution/Monitor. Warfarin's less potent R-enantiomer is metabolized in part by CYP3A4 (and also CYP1A2 and CYP2C19). Monitor INR more frequently if co-administered with inhibitors of these isoenzymes and adjust warfarin dose if needed

aspirin + potassium chloride	3	Aspirin and potassium chloride both increase serum potassium. Modify Therapy/Monitor Closely
bisoprolol + meloxicam	3	Bisoprolol and meloxicam both increase serum potassium. Use Caution/Monitor
budesonide + dexamethasone	3	Budesonide will decrease the level or effect of dexamethasone by affecting hepatic/intestinal enzyme CYP3A4 metabolism. Use Caution/Monitor
budesonide + enoxaparin	3	Budesonide, enoxaparin. Other (see comment). Use Caution/Monitor. Comment: Corticosteroids may decrease anticoagulant effects by increasing blood coagulability; conversely, they may impair vascular integrity, thus increasing bleeding risk. Monitor INR closely
dexamethasone + heparin	3	Dexamethasone, heparin. Other (see comment). Use Caution/Monitor. Comment: Corticosteroids may decrease anticoagulant effects by increasing blood coagulability; conversely, they may impair vascular integrity, thus increasing bleeding risk. Monitor INR closely
enalapril + insulin lispro	3	Enalapril increases effects of insulin lispro by pharmacodynamic synergism. Use Caution/Monitor
furosemide + potassium chloride	3	Potassium chloride increases and furosemide decreases serum potassium. Effect of interaction is not clear, use caution. Modify Therapy/Monitor Closely
heparin + losartan	3	Heparin increases toxicity of losartan by Other (see comment). Use Caution/Monitor. Comment: Low molecular weight heparins may suppress adrenal aldosterone secretion, which can potentially cause hyperkalemia
heparin + prednisolone	3	Prednisolone, heparin. Other (see comment). Use Caution/Monitor. Comment: Corticosteroids may decrease anticoagulant effects by increasing blood coagulability; conversely, they may impair vascular integrity, thus increasing bleeding risk. Monitor INR closely
hydrochlorothiazide + valsartan	3	Valsartan increases and hydrochlorothiazide decreases serum potassium. Effect of interaction is not clear, use caution. Use Caution/Monitor
indapamide + sotalol	3	Indapamide and sotalol both increase QTc interval. Contraindicated
indapamide + torsemide	3	Torsemide and indapamide both decrease serum potassium. Use Caution/Monitor
ketorolac + losartan	3	Ketorolac decreases effects of losartan by pharmacodynamic antagonism. Modify Therapy/Monitor Closely. NSAIDs decrease synthesis of vasodilating renal prostaglandins, and thus affect fluid homeostasis and may diminish antihypertensive effect
amiodarone + budesonide	2	Amiodarone will increase the level or effect of budesonide by P-glycoprotein (MDR1) efflux transporter. Use Caution/Monitor
amiodarone + nebivolol	2	Amiodarone will increase the level or effect of nebivolol by affecting hepatic enzyme CYP2D6 metabolism. Use Caution/Monitor. Monitor cardiac function carefully and observe for signs of bradycardia or heart block when amiodarone and a beta adrenergic blocker are co-administered. Amiodarone should be used with caution in patients receiving a beta adrenergic blocker, particularly if there is suspicion of underlying dysfunction of the sinus node, such as bradycardia or sick sinus syndrome, or if there is partial AV block. amioda-

		rone, nebivolol. Mechanism: pharmacodynamic synergism. Use Caution/Monitor. Risk of cardiotoxicity with bradycardia
amitriptyline + atorvastatin	2	Atorvastatin will increase the level or effect of amitriptyline by P-glycoprotein (MDR1) efflux transporter. Use Caution/Monitor
amoxicillin + aspirin	2	Amoxicillin, aspirin. Either increases levels of the other by plasma protein binding competition. Use Caution/Monitor. amoxicillin, aspirin. Either increases levels of the other by decreasing renal clearance. Use Caution/Monitor
ampicillin + aspirin	2	Ampicillin, aspirin. Either increases levels of the other by plasma protein binding competition. Use Caution/Monitor
aspirin + chlorthalidone	2	Aspirin increases and chlorthalidone decreases serum potassium. Effect of interaction is not clear, use caution. Use Caution/Monitor
aspirin + diclofenac	2	Aspirin and diclofenac both increase anticoagulation. Use Caution/Monitor
aspirin + doxazosin	2	Aspirin decreases effects of doxazosin by pharmacodynamic antagonism. Use Caution/Monitor. NSAIDs decrease prostaglandin synthesis
aspirin + fosinopril	2	Aspirin, fosinopril. Pharmacodynamic antagonism. Avoid or Use Alternate Drug. Co-administration may result in a significant decrease in renal function. NSAIDs may diminish the antihypertensive effect of ACE inhibitors. The mechanism of these interactions is likely related to the ability of NSAIDs to reduce the synthesis of vasodilating renal prostaglandins
aspirin + meloxicam	2	Aspirin and meloxicam both increase anticoagulation. Use Caution/Monitor
aspirin + nitroglycerin	2	Aspirin increases effects of nitroglycerin sublingual by additive vasodilation. Use Caution/Monitor. Vasodilatory and hemodynamic effects of NTG may be enhanced by co-administration with aspirin (additive effect desirable for emergent treatment)
aspirin + sotalol	2	Aspirin decreases effects of sotalol by pharmacodynamic antagonism. Use Caution/Monitor. Long term (>1 wk) NSAID use. NSAIDs decrease prostaglandin synthesis
atorvastatin + azithromycin	2	Azithromycin will increase the level or effect of atorvastatin by affecting hepatic/intestinal enzyme CYP3A4 metabolism. Use Caution/Monitor. If this combination is used, closely monitor for evidence of atorvastatin toxicity (eg, muscle aches or pains, renal dysfunction)
atorvastatin + prednisolone	2	Atorvastatin will increase the level or effect of prednisolone by P-glycoprotein (MDR1) efflux transporter. Use Caution/Monitor
azilsartan + ibuprofen	2	Ibuprofen, azilsartan. Either increases toxicity of the other by Other (see comment). Use Caution/Monitor. Comment: May result in renal function deterioration, particularly in elderly or volume depleted individuals. ibuprofen decreases effects of azilsartan by pharmacodynamic antagonism. Modify Therapy/Monitor Closely. NSAIDs decrease synthesis of vasodilating renal prostaglandins, and thus affect fluid homeostasis and may diminish antihypertensive effect
bisoprolol + candesartan	2	Bisoprolol, candesartan. Mechanism: pharmacodynamic synergism. Use Caution/Monitor. Risk of fetal compromise if given during pregnancy

bisoprolol + diclofenac	2	Bisoprolol and diclofenac both increase serum potassium. Use Caution/Monitor
bisoprolol + nifedipine	2	Bisoprolol, nifedipine. Either decreases effects of the other by pharmacodynamic synergism. Use Caution/Monitor. Both drugs lower blood pressure
calcium carbonate + metoprolol	2	Calcium carbonate decreases effects of metoprolol by unspecified interaction mechanism. Use Caution/Monitor
candesartan + enoxaparin	2	Enoxaparin increases toxicity of candesartan by Other (see comment). Use Caution/Monitor. Comment: Low molecular weight heparins may suppress adrenal aldosterone secretion, which can potentially cause hyperkalemia
candesartan + insulin aspart	2	Candesartan increases effects of insulin aspart by unspecified interaction mechanism. Use Caution/Monitor. Concomitant use of insulin and ARBs may require insulin dosage adjustment and increased glucose monitoring
candesartan + spironolactone	2	Candesartan and spironolactone both increase serum potassium. Modify Therapy/Monitor Closely
captopril + potassium chloride	2	Captopril increases levels of potassium chloride by decreasing elimination. Use Caution/Monitor. Risk of hyperkalemia
carvedilol + losartan	2	Carvedilol, losartan. Mechanism: pharmacodynamic synergism. Use Caution/Monitor. Risk of fetal compromise if given during pregnancy
cefotaxime + warfarin	2	Cefotaxime increases effects of warfarin by unspecified interaction mechanism. Use Caution/Monitor
ciprofloxacin + dexamethasone	2	Dexamethasone and ciprofloxacin both increase Other (see comment). Use Caution/Monitor. Co-administration of quinolone antibiotics and corticosteroids may increase risk of tendon rupture
clopidogrel + pantoprazole	2	Pantoprazole decreases effects of clopidogrel by affecting hepatic enzyme CYP2C19 metabolism. Use Caution/Monitor. Clopidogrel efficacy may be reduced by drugs that inhibit CYP2C19. Inhibition of platelet aggregation by clopidogrel is entirely due to the active clopidogrel metabolite. Clopidogrel is metabolized in part by CYP2C19. Pantoprazole prescribing information state that co-administration with clopidogrel had no clinically important effect on exposure to clopidogrel active metabolite; no dose adjustment of clopidogrel is required
dexamethasone + diclofenac	2	Diclofenac, dexamethasone. Either increases toxicity of the other by pharmacodynamic synergism. Use Caution/Monitor. Increased risk of GI ulceration
diclofenac + ketorolac	2	Diclofenac, ketorolac. Either increases toxicity of the other by pharmacodynamic synergism. Contraindicated
digoxin + indapamide	2	Digoxin increases and indapamide decreases serum potassium. Effect of interaction is not clear, use caution. Use Caution/Monitor
digoxin + potassium	2	Potassium chloride and digoxin both increase serum potassium. Modify Therapy/Monitor Closely
doxazosin + nifedipine	2	Doxazosin and nifedipine both increase anti-hypertensive channel blocking. Use Caution/Monitor
enalapril + enoxaparin	2	Enoxaparin increases toxicity of enalapril by Other (see comment). Use Cau-

		tion/Monitor. Comment: Low molecular weight heparins may suppress adrenal aldosterone secretion, which can potentially cause hyperkalemia
enalapril + furosemide	2	Enalapril, furosemide. Mechanism: pharmacodynamic synergism. Use Caution/Monitor. Risk of acute hypotension, renal insufficiency
enalapril + metformin	2	Enalapril increases toxicity of metformin by unspecified interaction mechanism. Use Caution/Monitor. Increases risk for hypoglycemia and lactic acidosis
enalapril + potassium	2	Enalapril increases levels of potassium chloride by decreasing elimination. Use Caution/Monitor. Risk of hyperkalemia
enalapril + spironolactone	2	Enalapril, spironolactone. Mechanism: pharmacodynamic synergism. Use Caution/Monitor. Risk of hyperkalemia
fosinopril + torsemide	2	Fosinopril, torsemide. Mechanism: pharmacodynamic synergism. Use Caution/Monitor. Risk of acute hypotension, renal insufficiency
furosemide + metoprolol	2	Metoprolol increases and furosemide decreases serum potassium. Effect of interaction is not clear, use caution. Use Caution/Monitor
heparin + lisinopril	2	Heparin increases toxicity of lisinopril by Other (see comment). Use Caution/Monitor. Comment: Low molecular weight heparins may suppress adrenal aldosterone secretion, which can potentially cause hyperkalemia
hydrochlorothiazide + sotalol	2	Sotalol increases and hydrochlorothiazide decreases serum potassium. Effect of interaction is not clear, use caution. Use Caution/Monitor
indapamide + ketorolac	2	Indapamide will increase the level or effect of ketorolac by acidic (anionic) drug competition for renal tubular clearance. Minor/Significance Unknown
ketorolac + lisinopril	2	Ketorolac, lisinopril. Pharmacodynamic antagonism. Avoid or Use Alternate Drug. Co-administration may result in a significant decrease in renal function. NSAIDs may diminish the antihypertensive effect of ACE inhibitors. The mechanism of these interactions is likely related to the ability of NSAIDs to reduce the synthesis of vasodilating renal prostaglandins
ketorolac + meloxicam	2	Ketorolac and meloxicam both increase anticoagulation. Use Caution/Monitor
ketorolac + rivaroxaban	2	Rivaroxaban, ketorolac. Other (see comment). Use Caution/Monitor. Comment: NSAIDs are known to increase bleeding. Bleeding risk may be increased when NSAIDs are used concomitantly with rivaroxaban. Monitor for signs/symptoms of blood loss
levofloxacin + prednisolone	2	Prednisolone and levofloxacin both increase Other (see comment). Use Caution/Monitor. Co-administration of quinolone antibiotics and corticosteroids may increase risk of tendon rupture
losartan + bisoprolol	2	Losartan and bisoprolol both increase serum potassium. Use Caution/Monitor
losartan + nebivolol	2	Losartan and nebivolol both increase serum potassium. Use Caution/Monitor
losartan + potassium	2	Losartan and potassium chloride both increase serum potassium. Use Caution/Monitor
losartan + sotalol	2	Losartan and sotalol both increase serum potassium. Use Caution/Monitor
metformin + perindopril	2	Perindopril increases toxicity of metformin by unspecified interaction mecha-

		nism. Use Caution/Monitor. Increases risk for hypoglycemia and lactic acidosis
metoprolol + nifedipine	2	Metoprolol and nifedipine both increase anti-hypertensive channel blocking. Modify Therapy/Monitor Closely
metoprolol + sotalol	2	Metoprolol and sotalol both increase anti-hypertensive channel blocking. Avoid or Use Alternate Drug
potassium + torsemide	2	Potassium chloride increases and torsemide decreases serum potassium. Effect of interaction is not clear, use caution. Modify Therapy/Monitor Closely
acetaminophen + heparin	1	Acetaminophen increases effects of heparin by unknown mechanism. Minor/Significance Unknown
amiodarone + atenolol	1	Amiodarone, atenolol. Mechanism: pharmacodynamic synergism. Use Caution/Monitor. Risk of cardiotoxicity with bradycardia
amiodarone + lidocaine	1	Amiodarone increases levels of lidocaine by affecting hepatic/intestinal enzyme CYP3A4 metabolism. Modify Therapy/Monitor Closely. Higher doses of amiodarone (ie, 600 mg BID) were shown to significantly increase lidocaine levels
amiodarone + prednisolone	1	Amiodarone will increase the level or effect of prednisolone by P-glycoprotein (MDR1) efflux transporter. Use Caution/Monitor
amlodipine + nebivolol	1	Nebivolol, amlodipine. Either increases effects of the other by pharmacodynamic synergism. Use Caution/Monitor. Both drugs lower blood pressure
ampicillin + omeprazole	1	Omeprazole will decrease the level or effect of ampicillin by increasing gastric pH. Applies only to oral form of both agents. Use Caution/Monitor
aspirin + escitalopram	1	Escitalopram, aspirin. Either increases toxicity of the other by pharmacodynamic synergism. Use Caution/Monitor. Increased risk of upper GI bleeding. SSRIs inhib. serotonin uptake by platelets
aspirin + glimepiride	1	Aspirin increases effects of glimepiride by unknown mechanism. Use Caution/Monitor. Risk of hypoglycemia
aspirin + indapamide	1	Aspirin increases and indapamide decreases serum potassium. Effect of interaction is not clear, use caution. Use Caution/Monitor
aspirin + ticagrelor	1	Aspirin, ticagrelor. Other (see comment). Use Caution/Monitor. Comment: Maintenance doses of aspirin above 100 mg decreases effectiveness of ticagrelor. Therefore, after the initial loading dose of aspirin (usually 325 mg), use ticagrelor with a maintenance dose of aspirin of 75-100 mg
atenolol + chlorthalidone	1	Atenolol increases and chlorthalidone decreases serum potassium. Effect of interaction is not clear, use caution. Use Caution/Monitor
atenolol + indapamide	1	Atenolol increases and indapamide decreases serum potassium. Effect of interaction is not clear, use caution. Use Caution/Monitor
atenolol + potassium	1	Atenolol and potassium chloride both increase serum potassium. Modify Therapy/Monitor Closely
atenolol + sotalol	1	Atenolol and sotalol both increase serum potassium. Use Caution/Monitor
atorvastatin + dabigatran	1	Atorvastatin will increase the level or effect of dabigatran by P-glycoprotein

		(MDR1) efflux transporter. Use Caution/Monitor. Atrial fibrillation: Avoid co-administering dabigatran with P-gp inhibitors if CrCl <30 mL/min. DVT/PE treatment: Avoid co-administering dabigatran with P-gp inhibitors if CrCl <50 mL/min
atorvastatin + telmisartan	1	Telmisartan increases toxicity of atorvastatin by Other (see comment). Use Caution/Monitor. Comment: OATP1B1 inhibitors may increase risk of myopathy
azithromycin + warfarin	1	Azithromycin increases toxicity of warfarin by anticoagulation. Use Caution/Monitor. Postmarketing reports have suggested that concomitant administration of azithromycin may potentiate effects of oral warfarin but the interaction does not appear to alter prothrombin time
bisoprolol + doxazosin	1	Doxazosin and bisoprolol both increase anti-hypertensive channel blocking. Modify Therapy/Monitor Closely
bisoprolol + insulin degludec	1	Bisoprolol, insulin degludec. Other (see comment). Modify Therapy/Monitor Closely. Comment: Beta-blockers may either increase or decrease the blood glucose lowering effect of insulin; beta-blockers can prolong hypoglycemia (interference with glycogenolysis) or cause hyperglycemia (insulin secretion inhibited)
bisoprolol + ivabradine	1	Ivabradine, bisoprolol. Either increases effects of the other by pharmacodynamic synergism. Modify Therapy/Monitor Closely. Most patients receiving ivabradine will also be treated with a beta-blocker. The risk of bradycardia increases with co-administration of drugs that slow heart rate (eg, digoxin, amiodarone, beta-blockers). Monitor heart rate in patients taking ivabradine with other negative chronotropes
bisoprolol + theophylline	1	Bisoprolol, theophylline. Other (see comment). Use Caution/Monitor. Comment: Beta blockers (esp. non selective) antagonize theophylline effects, while at the same time increasing theophylline levels and toxicity (mechanism: decreased theophylline metabolism). Smoking increases risk of interaction
budesonide + heparin	1	Budesonide, heparin. Other (see comment). Use Caution/Monitor. Comment: Corticosteroids may decrease anticoagulant effects by increasing blood coagulability; conversely, they may impair vascular integrity, thus increasing bleeding risk. Monitor INR closely
calcium carbonate + nifedipine	1	Calcium carbonate decreases effects of nifedipine by pharmacodynamic antagonism. Use Caution/Monitor
candesartan + dalteparin	1	Dalteparin increases toxicity of candesartan by Other (see comment). Use Caution/Monitor. Comment: Low molecular weight heparins may suppress adrenal aldosterone secretion, which can potentially cause hyperkalemia
candesartan + torsemide	1	Candesartan increases and torsemide decreases serum potassium. Effect of interaction is not clear, use caution. Use Caution/Monitor
captopril + furosemide	1	Captopril, furosemide. Mechanism: pharmacodynamic synergism. Use Caution/Monitor. Risk of acute hypotension, renal insufficiency
captopril + losartan	1	Losartan, captopril. Either increases toxicity of the other by pharmacodynamic synergism. Avoid or Use Alternate Drug. Dual blockade of renin-angiotensin system increases risks of hypotension, hyperkalemia, and renal impairment

carvedilol + indapamide	1	Carvedilol increases and indapamide decreases serum potassium. Effect of interaction is not clear, use caution. Use Caution/Monitor
chlorthalidone + ibuprofen	1	Chlorthalidone will increase the level or effect of ibuprofen by acidic (anionic) drug competition for renal tubular clearance. Minor/Significance Unknown
chlorthalidone + metoprolol	1	Metoprolol increases and chlorthalidone decreases serum potassium. Effect of interaction is not clear, use caution. Use Caution/Monitor
ciprofloxacin + diclofenac	1	Diclofenac, ciprofloxacin. Other (see comment). Modify Therapy/Monitor Closely. Comment: Mechanism: unknown. Increased risk of CNS stimulation and seizures with high doses of fluoroquinolones
clarithromycin + dexamethasone	1	Clarithromycin will increase the level or effect of dexamethasone by affecting hepatic/intestinal enzyme CYP3A4 metabolism. Avoid or Use Alternate Drug
clopidogrel + diclofenac	1	Clopidogrel, diclofenac. Either increases effects of the other by pharmacodynamic synergism. Modify Therapy/Monitor Closely. Clopidogrel and NSAIDs both inhibit platelet aggregation
clopidogrel + enoxaparin	1	Enoxaparin, clopidogrel. Either increases effects of the other by pharmacodynamic synergism. Modify Therapy/Monitor Closely. Enhanced risk of hemorrhage; additive effects are intended when both drugs are prescribed as indicated for ACS
clopidogrel + ibuprofen	1	Clopidogrel, ibuprofen. Either increases effects of the other by pharmacodynamic synergism. Modify Therapy/Monitor Closely. Clopidogrel and NSAIDs both inhibit platelet aggregation
clopidogrel + piracetam	1	Piracetam increases effects of clopidogrel by pharmacodynamic synergism. Use Caution/Monitor
clopidogrel + torsemide	1	Clopidogrel increases levels of torsemide by decreasing metabolism. Minor/Significance Unknown
cyanocobalamin + omeprazole	1	Omeprazole decreases levels of cyanocobalamin by inhibition of GI absorption. Applies only to oral form of both agents. Minor/Significance Unknown
dalteparin + dexamethasone	1	Dexamethasone, dalteparin. Other (see comment). Use Caution/Monitor. Comment: Corticosteroids may decrease anticoagulant effects by increasing blood coagulability; conversely, they may impair vascular integrity, thus increasing bleeding risk. Monitor INR closely
dexamethasone + ketorolac	1	Ketorolac, dexamethasone. Either increases toxicity of the other by pharmacodynamic synergism. Use Caution/Monitor. Increased risk of GI ulceration
dexamethasone + levofloxacin	1	Dexamethasone and levofloxacin both increase Other (see comment). Use Caution/Monitor. Co-administration of quinolone antibiotics and corticosteroids may increase risk of tendon rupture
dexamethasone + theophylline	1	Dexamethasone will decrease the level or effect of theophylline by affecting hepatic/intestinal enzyme CYP3A4 metabolism. Use Caution/Monitor
diclofenac + enalapril	1	Diclofenac, enalapril. pharmacodynamic antagonism. Avoid or Use Alternate Drug. Co-administration may result in a significant decrease in renal function. NSAIDs may diminish the antihypertensive effect of ACE inhibitors. The mechanism of these interactions is likely related to the ability of NSAIDs to

		reduce the synthesis of vasodilating renal prostaglandins
diclofenac + heparin	1	Heparin and diclofenac both increase anticoagulation. Modify Therapy/Monitor Closely
diclofenac + lisinopril	1	Diclofenac, lisinopril. pharmacodynamic antagonism. Avoid or Use Alternate Drug. Co-administration may result in a significant decrease in renal function. NSAIDs may diminish the antihypertensive effect of ACE inhibitors. The mechanism of these interactions is likely related to the ability of NSAIDs to reduce the synthesis of vasodilating renal prostaglandins
diclofenac + potassium	1	Diclofenac and potassium chloride both increase serum potassium. Modify Therapy/Monitor Closely
digoxin + levofloxacin	1	Levofloxacin will increase the level or effect of digoxin by altering intestinal flora. Applies only to oral form of both agents. Use Caution/Monitor
digoxin + lisinopril	1	Lisinopril increases levels of digoxin by unspecified interaction mechanism. Use Caution/Monitor
doxazosin + ketorolac	1	Ketorolac decreases effects of doxazosin by pharmacodynamic antagonism. Use Caution/Monitor. NSAIDs decrease prostaglandin synthesis
doxazosin + metoprolol	1	Doxazosin and metoprolol both increase anti-hypertensive channel blocking. Modify Therapy/Monitor Closely
enalapril + heparin	1	Heparin increases toxicity of enalapril by Other (see comment). Use Caution/Monitor. Comment: Low molecular weight heparins may suppress adrenal aldosterone secretion, which can potentially cause hyperkalemia
enalapril + ketorolac	1	Enalapril, ketorolac. Either increases toxicity of the other by Other (see comment). Use Caution/Monitor. Comment: May result in renal function deterioration, particularly in elderly or volume depleted individuals
enoxaparin + lisinopril	1	Enoxaparin increases toxicity of lisinopril by Other (see comment). Use Caution/Monitor. Comment: Low molecular weight heparins may suppress adrenal aldosterone secretion, which can potentially cause hyperkalemia
escitalopram + omeprazole	1	Omeprazole will increase the level or effect of escitalopram by affecting hepatic enzyme CYP2C19 metabolism. Use Caution/Monitor
fluconazole + levofloxacin	1	Fluconazole and levofloxacin both increase QTc interval. Modify Therapy/Monitor Closely
fluconazole + omeprazole	1	Fluconazole will increase the level or effect of omeprazole by affecting hepatic enzyme CYP2C19 metabolism. Use Caution/Monitor
folic acid + torsemide	1	Torsemide decreases levels of folic acid by increasing renal clearance. Minor/Significance Unknown
furosemide + indapamide	1	Furosemide and indapamide both decrease serum potassium. Use Caution/Monitor
furosemide + insulin degludec	1	Furosemide decreases effects of insulin degludec by Other (see comment). Use Caution/Monitor. Comment: Diuretics may cause hyperglycemia and glycosuria in patients with diabetes mellitus, possibly by diuretic-induced hpokalemia

furosemide + ketorolac	1	Ketorolac increases and furosemide decreases serum potassium. Effect of interaction is not clear, use caution. Use Caution/Monitor
heparin + ketorolac	1	Heparin and ketorolac both increase anticoagulation. Modify Therapy/Monitor Closely
hydrochlorothiazide + telmisartan	1	Telmisartan increases and hydrochlorothiazide decreases serum potassium. Effect of interaction is not clear, use caution. Use Caution/Monitor
indapamide + potassium	1	Potassium chloride increases and indapamide decreases serum potassium. Effect of interaction is not clear, use caution. Modify Therapy/Monitor Closely
indapamide + spironolactone	1	Spironolactone increases and indapamide decreases serum potassium. Effect of interaction is not clear, use caution. Modify Therapy/Monitor Closely
insulin regular human + metformin	1	Metformin, insulin regular human. Either increases effects of the other by pharmacodynamic synergism. Use Caution/Monitor. Antidiabetic agents are often used in combination; dosage adjustments may be required when initiating or discontinuing antidiabetic agents
ivabradine + metoprolol	1	Ivabradine, metoprolol. Either increases effects of the other by pharmacodynamic synergism. Modify Therapy/Monitor Closely. Most patients receiving ivabradine will also be treated with a beta-blocker. The risk of bradycardia increases with co-administration of drugs that slow heart rate (eg, digoxin, amiodarone, beta-blockers). Monitor heart rate in patients taking ivabradine with other negative chronotropes
ketorolac + potassium	1	Ketorolac and potassium chloride both increase serum potassium. Modify Therapy/Monitor Closely
ketorolac + spironolactone	1	Spironolactone and ketorolac both increase serum potassium. Modify Therapy/Monitor Closely
levofloxacin + magnesium sulfate	1	Magnesium sulfate decreases levels of levofloxacin by inhibition of GI absorption. Applies only to oral form of both agents. Use Caution/Monitor. Separate by 2 hours
levofloxacin + octreotide	1	Levofloxacin and octreotide both increase QTc interval. Modify Therapy/Monitor Closely
levofloxacin + warfarin	1	Levofloxacin increases effects of warfarin by unspecified interaction mechanism. Use Caution/Monitor
levothyroxine + metformin	1	Levothyroxine decreases effects of metformin by pharmacodynamic antagonism. Use Caution/Monitor. Patient should be closely observed for loss of blood glucose control; when drugs are withdrawn from a patient receiving metformin, patient should be observed closely for hypoglycemia
levothyroxine + warfarin	1	Levothyroxine increases effects of warfarin by unspecified interaction mechanism. Use Caution/Monitor
lisinopril + meloxicam	1	Lisinopril, meloxicam. Either increases toxicity of the other by Other (see comment). Use Caution/Monitor. Comment: May result in renal function deterioration, particularly in elderly or volume depleted individuals
losartan + aspirin	1	Losartan, aspirin. Either increases toxicity of the other by Other (see comment). Use Caution/Monitor. Comment: May result in renal function deterio-

		ration, particularly in elderly or volume depleted individuals
metformin + verapamil	1	Metformin will increase the level or effect of verapamil by basic (cationic) drug competition for renal tubular clearance. Minor/Significance Unknown
metoprolol + potassium	1	Metoprolol and potassium chloride both increase serum potassium. Modify Therapy/Monitor Closely
nifedipine + sotalol	1	Sotalol and nifedipine both increase anti-hypertensive channel blocking. Modify Therapy/Monitor Closely
perindopril + spironolactone	1	Perindopril, spironolactone. Mechanism: pharmacodynamic synergism. Use Caution/Monitor. Risk of hyperkalemia
potassium chloride + sotalol	1	Sotalol and potassium chloride both increase serum potassium. Modify Therapy/Monitor Closely
spironolactone + valsartan	1	Valsartan and spironolactone both increase serum potassium. Modify Therapy/Monitor Closely
telmisartan + torsemide	1	Telmisartan increases and torsemide decreases serum potassium. Effect of interaction is not clear, use caution. Use Caution/Monitor
torsemide + metformin	1	Torsemide decreases effects of metformin by pharmacodynamic antagonism. Use Caution/Monitor
valsartan + bisoprolol	1	Valsartan and bisoprolol both increase serum potassium. Use Caution/Monitor

***Note:** Impact of drug-drug interactions associated with the combinations 'aspirin + captopril' and 'aspirin + enalapril' may be considered insignificant due to the use of low-dose aspirin in the majority of cases. Administration of aspirin at doses less than 300 mg per day has little effect on the effectiveness of captopril and enalapril. Administration of aspirin in higher doses reduces the effectiveness of captopril and enalapril.

Table S4. The detailed explanation of the entire list of pairwise minor drug-drug interactions among taken drugs in the cohort of cardiovascular patients based on data of electronic health records in 2019-2022 (*n* = 704).

Drug combination	n	Minor drug-drug interactions (T-List)
clopidogrel + torsemide	13	Clopidogrel increases levels of torsemide by decreasing metabolism. Minor/Significance Unknown
aspirin + furosemide	12	Aspirin decreases effects of furosemide by pharmacodynamic antagonism. Minor/Significance Unknown. NSAIDs decrease prostaglandin synthesis
furosemide + metformin	12	Furosemide increases levels of metformin by unspecified interaction mechanism. Minor/Significance Unknown. Patient should be closely observed for loss of blood glucose control; when drugs are withdrawn from a patient receiving metformin, patient should be observed closely for hypoglycemia
aspirin + indapamide	11	Indapamide will increase the level or effect of aspirin by acidic (anionic) drug competition for renal tubular clearance. Minor/Significance Unknown
aspirin + hydrochlorothiazide	9	Hydrochlorothiazide will increase the level or effect of aspirin by acidic (anionic) drug competition for renal tubular clearance. Minor/Significance Unknown
metformin + nifedipine	7	Nifedipine increases levels of metformin by enhancing GI absorption. Applies only to oral form of both agents. Minor/Significance Unknown
ascorbic acid + aspirin	6	Ascorbic acid will increase the level or effect of aspirin by acidic (anionic) drug competition for renal tubular clearance. Minor/Significance Unknown. Ascorbic acid increases levels of aspirin by decreasing renal clearance. Minor/Significance Unknown
carvedilol + omeprazole	6	Omeprazole will increase the level or effect of carvedilol by affecting hepatic enzyme CYP2C9/10 metabolism. Minor/Significance Unknown
hydrochlorothiazide + metformin	6	Hydrochlorothiazide decreases effects of metformin by pharmacodynamic antagonism. Minor/Significance Unknown. Thiazide dosage >50 mg/day may increase blood glucose. hydrochlorothiazide will increase the level or effect of metformin by basic (cationic) drug competition for renal tubular clearance. Minor/Significance Unknown
acetaminophen + enoxaparin	5	Acetaminophen increases effects of enoxaparin by unknown mechanism. Minor/Significance Unknown
dexamethasone + omeprazole	5	Dexamethasone will decrease the level or effect of omeprazole by affecting hepatic/intestinal enzyme CYP3A4 metabolism. Minor/Significance Unknown
indapamide + metformin	5	Indapamide decreases effects of metformin by pharmacodynamic antagonism. Minor/Significance Unknown. Thiazide dosage >50 mg/day may increase blood glucose
magnesium hydroxide + torsemide	5	Torsemide decreases levels of magnesium hydroxide by increasing renal clearance. Minor/Significance Unknown
ceftriaxone + furosemide	4	Ceftriaxone increases toxicity of furosemide by pharmacodynamic synergism. Minor/Significance Unknown. Increased risk of nephrotoxicity
dexamethasone + insulin	4	Dexamethasone decreases effects of insulin lispro by pharmacodynamic an-

		tagonism. Minor/Significance Unknown
folic acid + torsemide	4	Torsemide decreases levels of folic acid by increasing renal clearance. Minor/Significance Unknown
amiodarone + pyridoxine	3	Aspirin will increase the level or effect of ketorolac by acidic (anionic) drug competition for renal tubular clearance. Minor/Significance Unknown
aspirin + ketorolac	3	Aspirin will increase the level or effect of ketorolac by acidic (anionic) drug competition for renal tubular clearance. Minor/Significance Unknown
B12 + metformin	3	Metformin decreases levels of cyanocobalamin by unspecified interaction mechanism. Minor/Significance Unknown. It may take several years of metformin therapy to develop vitamin B12 deficiency
B12 + omeprazole	3	Omeprazole decreases levels of cyanocobalamin by inhibition of GI absorption. Applies only to oral form of both agents. Minor/Significance Unknown
folic acid + metformin	3	Metformin decreases levels of folic acid by unspecified interaction mechanism. Minor/Significance Unknown
aspirin + B12	2	Aspirin decreases levels of cyanocobalamin by inhibition of GI absorption. Applies only to oral form of both agents. Minor/Significance Unknown
aspirin + dexamethasone	2	Dexamethasone decreases levels of aspirin by increasing renal clearance. Minor/Significance Unknown
aspirin + folic acid	2	Aspirin decreases levels of folic acid by inhibition of GI absorption. Applies only to oral form of both agents. Minor/Significance Unknown
budesonide + torsemide	2	Budesonide, torsemide. Mechanism: pharmacodynamic synergism. Minor/Significance Unknown. Risk of hypokalemia, especially with strong glucocorticoid activity
chlorthalidone + magnesium hydroxide	2	Chlorthalidone decreases levels of magnesium hydroxide by increasing renal clearance. Minor/Significance Unknown
indapamide + ketorolac	2	Indapamide will increase the level or effect of ketorolac by acidic (anionic) drug competition for renal tubular clearance. Minor/Significance Unknown
indapamide + magnesium	3	Indapamide decreases levels of magnesium chloride by increasing renal clearance. Minor/Significance Unknown
indapamide + magnesium hydroxide	2	Indapamide decreases levels of magnesium hydroxide by increasing renal clearance. Minor/Significance Unknown
indapamide + vildagliptin	2	Indapamide decreases effects of vildagliptin by pharmacodynamic antagonism. Minor/Significance Unknown. Thiazide dosage >50 mg/day may increase blood glucose
magnesium hydroxide + spironolactone	2	Spironolactone increases levels of magnesium hydroxide by decreasing renal clearance. Minor/Significance Unknown
omeprazole + theophylline	2	Omeprazole will decrease the level or effect of theophylline by affecting hepatic enzyme CYP1A2 metabolism. Minor/Significance Unknown. omeprazole increases toxicity of theophylline by Other (see comment). Minor/Significance Unknown. Comment: Prolonged use of proton pump inhibitors can cause hypochlorhydria, which in turn causes peristalsis in small intestine to increase

		and peristalsis in the proximal colon to decrease; monitor for toxicity
thiamine + torsemide	2	Torsemide decreases levels of thiamine by increasing renal clearance. Minor/Significance Unknown
acetaminophen + heparin	1	Acetaminophen increases effects of heparin by unknown mechanism. Minor/Significance Unknown
acetazolamide + dexamethasone	1	Acetazolamide will increase the level or effect of dexamethasone by affecting hepatic/intestinal enzyme CYP3A4 metabolism. Minor/Significance Unknown
acetylcysteine + nitroglycerin IV	1	Acetylcysteine increases effects of nitroglycerin IV by Other (see comment). Minor/Significance Unknown. Comment: Acetylcysteine may enhance vasodilatory effects of nitroglycerin
amikacin + fluconazole	1	Fluconazole decreases levels of amikacin by unknown mechanism. Minor/Significance Unknown
amitriptyline + dexamethasone	1	Dexamethasone will decrease the level or effect of amitriptyline by affecting hepatic/intestinal enzyme CYP3A4 metabolism. Minor/Significance Unknown
amitriptyline + metformin	1	Amitriptyline increases effects of metformin by pharmacodynamic synergism. Minor/Significance Unknown
aspirin + calcium carbonate	1	Calcium carbonate, aspirin. Mechanism: passive renal tubular reabsorption due to increased pH. Minor/Significance Unknown. Salicylate levels increased at moderate doses; salicylate levels decreased at large doses (d/t increased renal excretion of unchanged salicylic acid)
aspirin + chlorthalidone	2	Aspirin increases and chlorthalidone decreases serum potassium. Effect of interaction is not clear, use caution. Use Caution/Monitor
aspirin + diclofenac	1	Aspirin will increase the level or effect of diclofenac by acidic (anionic) drug competition for renal tubular clearance. Minor/Significance Unknown
aspirin + eplerenone	1	Aspirin decreases effects of eplerenone by pharmacodynamic antagonism. Minor/Significance Unknown. NSAIDs decrease prostaglandin synthesis
aspirin + glimepiride	1	Aspirin increases effects of glimepiride by plasma protein binding competition. Minor/Significance Unknown. Large dose of salicylate
aspirin + meloxicam	1	Aspirin will increase the level or effect of meloxicam by acidic (anionic) drug competition for renal tubular clearance. Minor/Significance Unknown
azithromycin + levofloxacin	1	Azithromycin and levofloxacin both increase QTc interval. Minor/Significance Unknown
budesonide + furosemide	1	Budesonide, furosemide. Mechanism: pharmacodynamic synergism. Minor/Significance Unknown. Risk of hypokalemia, especially with strong glucocorticoid activity
budesonide + insulin	1	Budesonide decreases effects of insulin regular human by pharmacodynamic antagonism. Minor/Significance Unknown
budesonide + metformin	1	Budesonide decreases effects of metformin by pharmacodynamic antagonism. Minor/Significance Unknown
chlorthalidone + ibuprofen	1	Chlorthalidone will increase the level or effect of ibuprofen by acidic (anionic) drug competition for renal tubular clearance. Minor/Significance Unknown

chlorthalidone + metformin	1	Chlorthalidone decreases effects of metformin by pharmacodynamic antagonism. Minor/Significance Unknown. Thiazide dosage >50 mg/day may increase blood glucose
clarithromycin + dexamethasone	1	Dexamethasone will decrease the level or effect of clarithromycin by affecting hepatic/intestinal enzyme CYP3A4 metabolism. Minor/Significance Unknown
dexamethasone + torsemide	1	Dexamethasone, torsemide. Mechanism: pharmacodynamic synergism. Minor/Significance Unknown. Risk of hypokalemia, especially with strong glucocorticoid activity
dextrose + magnesium sulfate	1	Dextrose decreases levels of magnesium sulfate by increasing renal clearance. Minor/Significance Unknown
diclofenac + ketorolac	1	Diclofenac will increase the level or effect of ketorolac by acidic (anionic) drug competition for renal tubular clearance. Minor/Significance Unknown
folic acid + furosemide	1	Furosemide decreases levels of folic acid by increasing renal clearance. Minor/Significance Unknown
furosemide + ketorolac	1	Ketorolac increases and furosemide decreases serum potassium. Effect of interaction is not clear, use caution. Use Caution/Monitor
furosemide + magnesium hydroxide	1	Furosemide decreases levels of magnesium hydroxide by increasing renal clearance. Minor/Significance Unknown
furosemide + magnesium sulfate	1	Furosemide decreases levels of magnesium sulfate by increasing renal clearance. Minor/Significance Unknown
heparin + nitroglycerin IV	1	Nitroglycerin IV decreases effects of heparin by unspecified interaction mechanism. Minor/Significance Unknown
hydrochlorothiazide + magnesium hydroxide	1	Hydrochlorothiazide decreases levels of magnesium hydroxide by increasing renal clearance. Minor/Significance Unknown
insulin + potassium	2	Potassium chloride increases effects of insulin regular human by pharmacodynamic synergism. Minor/Significance Unknown. Interaction especially seen in the treatment of hypokalemia
ketorolac + meloxicam	1	Ketorolac will increase the level or effect of meloxicam by acidic (anionic) drug competition for renal tubular clearance. Minor/Significance Unknown
lidocaine + omeprazole	1	Omeprazole will decrease the level or effect of lidocaine by affecting hepatic enzyme CYP1A2 metabolism. Minor/Significance Unknown
magnesium + torsemide	1	Torsemide decreases levels of magnesium hydroxide by increasing renal clearance. Minor/Significance Unknown
metformin + potassium	1	Potassium chloride increases effects of metformin by pharmacodynamic synergism. Minor/Significance Unknown. Interaction especially seen in the treatment of hypokalemia
metformin + verapamil	1	Metformin will increase the level or effect of verapamil by basic (cationic) drug competition for renal tubular clearance. Minor/Significance Unknown
metoprolol + oxazepam	1	Metoprolol increases effects of oxazepam by decreasing metabolism. Minor/Significance Unknown
pentoxifylline + theophylline	1	Pentoxifylline increases levels of theophylline by decreasing metabolism.

		Minor/Significance Unknown
prednisolone + torsemide	1	Prednisolone, torsemide. Mechanism: pharmacodynamic synergism. Minor/Significance Unknown. Risk of hypokalemia, especially with strong glucocorticoid activity

Table S5. The detailed explanation of the entire list of pairwise serious drug-drug interactions among prescribed drugs in the cohort of cardiovascular patients based on data of electronic health records in 2019-2022 (n = 704).

Drug combination	n	Serious drug-drug interactions (P-List)
aspirin + captopril*	62	Aspirin, captopril. Pharmacodynamic antagonism. Avoid or Use Alternate Drug. Co-administration may result in a significant decrease in renal function. NSAIDs may diminish the antihypertensive effect of ACE inhibitors. The mechanism of these interactions is likely related to the ability of NSAIDs to reduce the synthesis of vasodilating renal prostaglandins
captopril + losartan	38	Losartan, captopril. Either increases toxicity of the other by pharmacodynamic synergism. Avoid or Use Alternate Drug. Dual blockade of renin-angiotensin system increases risks of hypotension, hyperkalemia, and renal impairment
aspirin + lisinopril	34	Aspirin, lisinopril. pharmacodynamic antagonism. Avoid or Use Alternate Drug. Co-administration may result in a significant decrease in renal function. NSAIDs may diminish the antihypertensive effect of ACE inhibitors. The mechanism of these interactions is likely related to the ability of NSAIDs to reduce the synthesis of vasodilating renal prostaglandins
aspirin + perindopril	34	Aspirin, perindopril. pharmacodynamic antagonism. Avoid or Use Alternate Drug. Co-administration may result in a significant decrease in renal function. NSAIDs may diminish the antihypertensive effect of ACE inhibitors. The mechanism of these interactions is likely related to the ability of NSAIDs to reduce the synthesis of vasodilating renal prostaglandins
clopidogrel + omeprazole	30	Omeprazole decreases effects of clopidogrel by affecting hepatic enzyme CYP2C19 metabolism. Avoid or Use Alternate Drug. Clopidogrel efficacy may be reduced by drugs that inhibit CYP2C19. Inhibition of platelet aggregation by clopidogrel is entirely due to an active metabolite. Clopidogrel is metabolized to this active metabolite in part by CYP2C19
amiodarone + indapamide	9	Amiodarone and indapamide both increase QTc interval. Avoid or Use Alternate Drug
aspirin + enalapril*	7	Aspirin, enalapril. Pharmacodynamic antagonism. Avoid or Use Alternate Drug. Co-administration may result in a significant decrease in renal function. NSAIDs may diminish the antihypertensive effect of ACE inhibitors. The mechanism of these interactions is likely related to the ability of NSAIDs to reduce the synthesis of vasodilating renal prostaglandins
aspirin + fosinopril	7	Aspirin, fosinopril. pharmacodynamic antagonism. Avoid or Use Alternate Drug. Co-administration may result in a significant decrease in renal function. NSAIDs may diminish the antihypertensive effect of ACE inhibitors. The mechanism of these interactions is likely related to the ability of NSAIDs to reduce the synthesis of vasodilating renal prostaglandins
bisoprolol + digoxin	6	Bisoprolol increases effects of digoxin by pharmacodynamic synergism. Use Caution/Monitor. Enhanced bradycardia
candesartan + captopril	6	Candesartan, captopril. Either increases toxicity of the other by pharmacodynamic synergism. Avoid or Use Alternate Drug. Dual blockade of renin-angiotensin system increases risks of hypotension, hyperkalemia, and renal impairment

azilsartan + captopril	5	Azilsartan, captopril. Either increases toxicity of the other by pharmacodynamic synergism. Avoid or Use Alternate Drug. Dual blockade of renin-angiotensin system increases risks of hypotension, hyperkalemia, and renal impairment
captopril + valsartan	5	Valsartan, captopril. Either increases toxicity of the other by pharmacodynamic synergism. Avoid or Use Alternate Drug. Dual blockade of renin-angiotensin system increases risks of hypotension, hyperkalemia, and renal impairment
amlodipine + nifedipine	4	Nifedipine will increase the level or effect of amlodipine by affecting hepatic/intestinal enzyme CYP3A4 metabolism. Avoid or Use Alternate Drug
apixaban + clopidogrel	3	Clopidogrel and apixaban both increase anticoagulation. Avoid or Use Alternate Drug
captopril + telmisartan	3	Telmisartan, captopril. Either increases toxicity of the other by pharmacodynamic synergism. Avoid or Use Alternate Drug. Dual blockade of renin-angiotensin system increases risks of hypotension, hyperkalemia, and renal impairment
ibuprofen + naproxen	3	Ibuprofen will increase the level or effect of naproxen by acidic (anionic) drug competition for renal tubular clearance. Avoid or Use Alternate Drug. Therapeutic duplication. ibuprofen and naproxen both increase anticoagulation. Avoid or Use Alternate Drug. Therapeutic duplication. ibuprofen and naproxen both increase anticoagulation. Avoid or Use Alternate Drug. Therapeutic duplication
apixaban + rivaroxaban	2	Rivaroxaban and apixaban both increase anticoagulation. Avoid or Use Alternate Drug
aspirin + ramipril	2	Aspirin, ramipril. pharmacodynamic antagonism. Avoid or Use Alternate Drug. Co-administration may result in a significant decrease in renal function. NSAIDs may diminish the antihypertensive effect of ACE inhibitors. The mechanism of these interactions is likely related to the ability of NSAIDs to reduce the synthesis of vasodilating renal prostaglandins
bisoprolol + nebivolol	2	Bisoprolol and nebivolol both increase anti-hypertensive channel blocking. Avoid or Use Alternate Drug
bisoprolol + timolol	2	Bisoprolol and timolol both increase anti-hypertensive channel blocking. Avoid or Use Alternate Drug
digoxin + metoprolol	2	Digoxin increases toxicity of metoprolol by unspecified interaction mechanism. Avoid or Use Alternate Drug. Can increase risk of bradycardia
digoxin + pantoprazole	2	Pantoprazole will increase the level or effect of digoxin by increasing gastric pH. Applies only to oral form of both agents. Avoid or Use Alternate Drug
potassium + spironolactone	2	Spironolactone and potassium chloride both increase serum potassium. Avoid or Use Alternate Drug
aspirin + ibuprofen	2	Ibuprofen increases toxicity of aspirin by anticoagulation. Avoid or Use Alternate Drug. increases risk of bleeding. ibuprofen decreases effects of aspirin by Other (see comment). Avoid or Use Alternate Drug. Comment: Ibuprofen decreases the antiplatelet effects of low-dose aspirin by blocking the active site

		of platelet cyclooxygenase. Administer ibuprofen 8 h before aspirin or at least 2-4 h after aspirin. The effect of other NSAIDs on aspirin is not established
amiodarone + carbamazepine	1	Carbamazepine will decrease the level or effect of amiodarone by affecting hepatic/intestinal enzyme CYP3A4 metabolism. Avoid or Use Alternate Drug
amlodipine + simvastatin	1	Amlodipine increases levels of simvastatin by Other (see comment). Avoid or Use Alternate Drug. Comment: Benefits of combination therapy should be carefully weighed against the potential risks of combination. Potential for increased risk of myopathy/rhabdomyolysis. Limit simvastatin dose to no more than 20 mg/day when used concurrently
atorvastatin + carbamazepine	1	Carbamazepine will decrease the level or effect of atorvastatin by affecting hepatic/intestinal enzyme CYP3A4 metabolism. Avoid or Use Alternate Drug
atorvastatin + niacin	1	Niacin, atorvastatin. Either increases toxicity of the other by pharmacodynamic synergism. Avoid or Use Alternate Drug. Increased risk of rhabdomyolysis (>1 g/day niacin)
budesonide + clarithromycin	1	Clarithromycin will increase the level or effect of budesonide by affecting hepatic/intestinal enzyme CYP3A4 metabolism. Avoid or Use Alternate Drug
captopril + ketoprofen	1	Ketoprofen, captopril. pharmacodynamic antagonism. Avoid or Use Alternate Drug. Co-administration may result in a significant decrease in renal function. NSAIDs may diminish the antihypertensive effect of ACE inhibitors. The mechanism of these interactions is likely related to the ability of NSAIDs to reduce the synthesis of vasodilating renal prostaglandins
captopril + meloxicam	1	Meloxicam, captopril. pharmacodynamic antagonism. Avoid or Use Alternate Drug. Co-administration may result in a significant decrease in renal function. NSAIDs may diminish the antihypertensive effect of ACE inhibitors. The mechanism of these interactions is likely related to the ability of NSAIDs to reduce the synthesis of vasodilating renal prostaglandins
celecoxib + lisinopril	1	Celecoxib, lisinopril. pharmacodynamic antagonism. Avoid or Use Alternate Drug. Co-administration may result in a significant decrease in renal function. NSAIDs may diminish the antihypertensive effect of ACE inhibitors. The mechanism of these interactions is likely related to the ability of NSAIDs to reduce the synthesis of vasodilating renal prostaglandins
clopidogrel + esomeprazole	1	Esomeprazole decreases effects of clopidogrel by affecting hepatic enzyme CYP2C19 metabolism. Avoid or Use Alternate Drug. Clopidogrel efficacy may be reduced by drugs that inhibit CYP2C19. Inhibition of platelet aggregation by clopidogrel is entirely due to an active metabolite. Clopidogrel is metabolized to this active metabolite in part by CYP2C19
diclofenac + perindopril	1	Diclofenac, perindopril. pharmacodynamic antagonism. Avoid or Use Alternate Drug. Co-administration may result in a significant decrease in renal function. NSAIDs may diminish the antihypertensive effect of ACE inhibitors. The mechanism of these interactions is likely related to the ability of NSAIDs to reduce the synthesis of vasodilating renal prostaglandins
digoxin + esomeprazole	1	Esomeprazole will increase the level or effect of digoxin by increasing gastric pH. Applies only to oral form of both agents. Avoid or Use Alternate Drug
digoxin + omeprazole	1	Esomeprazole will increase the level or effect of digoxin by increasing gastric

		pH. Applies only to oral form of both agents. Avoid or Use Alternate Drug
fenofibrate + pitavastatin	1	Fenofibrate, pitavastatin. Either increases effects of the other by pharmacodynamic synergism. Avoid or Use Alternate Drug. Fenofibrate may further increase risk for rhabdomyolysis when added to optimal statin regimen to further decrease TG and increase HDLs
ibuprofen + lisinopril	1	Ibuprofen, lisinopril. pharmacodynamic antagonism. Avoid or Use Alternate Drug. Co-administration may result in a significant decrease in renal function. NSAIDs may diminish the antihypertensive effect of ACE inhibitors. The mechanism of these interactions is likely related to the ability of NSAIDs to reduce the synthesis of vasodilating renal prostaglandins
ketoprofen + ramipril	1	Ketoprofen, ramipril. pharmacodynamic antagonism. Avoid or Use Alternate Drug. Co-administration may result in a significant decrease in renal function. NSAIDs may diminish the antihypertensive effect of ACE inhibitors. The mechanism of these interactions is likely related to the ability of NSAIDs to reduce the synthesis of vasodilating renal prostaglandins
lisinopril + losartan	1	Losartan, lisinopril. Either increases toxicity of the other by pharmacodynamic synergism. Avoid or Use Alternate Drug. Dual blockade of renin-angiotensin system increases risks of hypotension, hyperkalemia, and renal impairment
meloxicam + perindopril	1	Meloxicam, perindopril. pharmacodynamic antagonism. Avoid or Use Alternate Drug. Co-administration may result in a significant decrease in renal function. NSAIDs may diminish the antihypertensive effect of ACE inhibitors. The mechanism of these interactions is likely related to the ability of NSAIDs to reduce the synthesis of vasodilating renal prostaglandins
methotrexate + naproxen	1	Naproxen increases levels of methotrexate by decreasing renal clearance. Avoid or Use Alternate Drug. Concomitant administration of NSAIDs with high dose methotrexate has been reported to elevate and prolong serum methotrexate levels, resulting in deaths from severe hematologic and GI toxicity. NSAIDs may reduce tubular secretion of methotrexate and enhance toxicity

***Note:** Impact of drug-drug interactions associated with the combinations 'aspirin + captopril' and 'aspirin + enalapril' may be considered insignificant due to the use of low-dose aspirin in the majority of cases. Administration of aspirin at doses less than 300 mg per day has little effect on the effectiveness of captopril and enalapril. Administration of aspirin in higher doses reduces the effectiveness of captopril and enalapril.

Table S6. The detailed explanation of the entire list of pairwise monitor-closely drug-drug interactions among prescribed drugs in the cohort of cardiovascular patients based on data of electronic health records in 2019-2022 (*n* = 704).

Drug combination	n	Monitor-closely drug-drug interactions (P-List)
aspirin + bisoprolol	180	Aspirin decreases effects of bisoprolol by pharmacodynamic antagonism. Use Caution/Monitor. Long term (>1 wk) NSAID use. NSAIDs decrease prostaglandin synthesis
aspirin + losartan	168	Aspirin decreases effects of losartan by pharmacodynamic antagonism. Modify Therapy/Monitor Closely. NSAIDs decrease synthesis of vasodilating renal prostaglandins, and thus affect fluid homeostasis and may diminish antihypertensive effect
aspirin + metoprolol	86	Aspirin decreases effects of metoprolol by pharmacodynamic antagonism. Use Caution/Monitor. Long term (>1 wk) NSAID use. NSAIDs decrease prostaglandin synthesis
bisoprolol + losartan	82	Bisoprolol, losartan. Mechanism: pharmacodynamic synergism. Use Caution/Monitor. Risk of fetal compromise if given during pregnancy
aspirin + captopril*	62	Aspirin, captopril. pharmacodynamic antagonism. Avoid or Use Alternate Drug. Co-administration may result in a significant decrease in renal function. NSAIDs may diminish the antihypertensive effect of ACE inhibitors. The mechanism of these interactions is likely related to the ability of NSAIDs to reduce the synthesis of vasodilating renal prostaglandins
aspirin + spironolactone	62	Aspirin decreases effects of spironolactone by unspecified interaction mechanism. Use Caution/Monitor. When used concomitantly, spironolactone dose may need to be titrated to higher maintenance dose and the patient should be observed closely to determine if the desired effect is obtained
aspirin + nitroglycerin	61	Aspirin increases effects of nitroglycerin sublingual by additive vasodilation. Use Caution/Monitor. Vasodilatory and hemodynamic effects of NTG may be enhanced by co-administration with aspirin (additive effect desirable for emergent treatment)
spironolactone + torsemide	56	Spironolactone increases and torsemide decreases serum potassium. Effect of interaction is not clear, use caution. Modify Therapy/Monitor Closely
bisoprolol + torsemide	55	Bisoprolol increases and torsemide decreases serum potassium. Effect of interaction is not clear, use caution. Use Caution/Monitor
aspirin + clopidogrel	46	Aspirin, clopidogrel. Either increases toxicity of the other by pharmacodynamic synergism. Use Caution/Monitor. The need for simultaneous use of low-dose aspirin and anticoagulant or antiplatelet agents are common for patients with cardiovascular disease; monitor closely
aspirin + torsemide	43	Aspirin increases and torsemide decreases serum potassium. Effect of interaction is not clear, use caution. Use Caution/Monitor
amlodipine + bisoprolol	41	Bisoprolol, amlodipine. Either increases effects of the other by pharmacodynamic synergism. Use Caution/Monitor. Both drugs lower blood pressure
losartan + torsemide	41	Losartan increases and torsemide decreases serum potassium. Effect of interaction is not clear, use caution. Use Caution/Monitor

captopril + torsemide	38	Captopril, torsemide. Either increases toxicity of the other by Mechanism: pharmacodynamic synergism. Use Caution/ Monitor. Risk of acute hypotension, renal insufficiency. Monitor blood pressure and renal function
losartan + metoprolol	38	Losartan and metoprolol both increase serum potassium. Use Caution/Monitor
aspirin + candesartan	36	Aspirin decreases effects of candesartan by pharmacodynamic antagonism. Modify Therapy/Monitor Closely. NSAIDs decrease synthesis of vasodilating renal prostaglandins, and thus affect fluid homeostasis and may diminish antihypertensive effect
aspirin + perindopril	35	Aspirin, perindopril. pharmacodynamic antagonism. Avoid or Use Alternate Drug. Co-administration may result in a significant decrease in renal function. NSAIDs may diminish the antihypertensive effect of ACE inhibitors. The mechanism of these interactions is likely related to the ability of NSAIDs to reduce the synthesis of vasodilating renal prostaglandins
amiodarone + atorvastatin	34	Amiodarone will increase the level or effect of atorvastatin by P-glycoprotein (MDR1) efflux transporter. Use Caution/Monitor
amiodarone + losartan	34	Amiodarone will increase the level or effect of losartan by affecting hepatic enzyme CYP2C9/10 metabolism. Use Caution/Monitor. May inhibit the conversion of losartan to its active metabolite E-3174. Importance of interaction not established; monitor individual therapeutic response to determine losartan dosage
aspirin + lisinopril	34	Aspirin, lisinopril. pharmacodynamic antagonism. Avoid or Use Alternate Drug. Co-administration may result in a significant decrease in renal function. NSAIDs may diminish the antihypertensive effect of ACE inhibitors. The mechanism of these interactions is likely related to the ability of NSAIDs to reduce the synthesis of vasodilating renal prostaglandins
bisoprolol + spironolactone	32	Bisoprolol and spironolactone both increase serum potassium. Modify Therapy/Monitor Closely
amlodipine + nitroglycerin	30	Amlodipine, nitroglycerin sublingual. Either increases toxicity of the other by additive vasodilation. Modify Therapy/ Monitor Closely. Marked orthostatic hypotension reported with concomitant use
aspirin + indapamide	30	Aspirin increases and indapamide decreases serum potassium. Effect of interaction is not clear, use caution. Use Caution/Monitor
losartan + omeprazole	27	Omeprazole will increase the level or effect of losartan by affecting hepatic enzyme CYP2C9/10 metabolism. Use Caution/Monitor. May inhibit the conversion of losartan to its active metabolite E-3174. Importance of interaction not established; monitor individual therapeutic response to determine losartan dosage
bisoprolol + indapamide	26	Bisoprolol increases and indapamide decreases serum potassium. Effect of interaction is not clear, use caution. Use Caution/Monitor
lisinopril + spironolactone	26	Lisinopril, spironolactone. Mechanism: pharmacodynamic synergism. Use Caution/Monitor. Risk of hyperkalemia
lisinopril + torsemide	26	Lisinopril, torsemide. Mechanism: pharmacodynamic synergism. Use Cau-

		tion/Monitor. Risk of acute hypotension, renal insufficiency
captopril + spironolactone	25	Captopril, spironolactone. Either increases toxicity of the other by Mechanism: pharmacodynamic synergism. Use Caution/ Monitor. Both drugs lower blood pressure. Risk of hyperkalemia. Monitor blood pressure and potassium
aspirin + valsartan	24	Aspirin decreases effects of valsartan by pharmacodynamic antagonism. Modify Therapy/Monitor Closely. NSAIDs decrease synthesis of vasodilating renal prostaglandins, and thus affect fluid homeostasis and may diminish antihypertensive effect
losartan + spironolactone	24	Losartan and spironolactone both increase serum potassium. Modify Therapy/ Monitor Closely
aspirin + carvedilol	22	Aspirin decreases effects of carvedilol by pharmacodynamic antagonism. Use Caution/Monitor. Long term (>1 wk) NSAID use. NSAIDs decrease prostaglandin synthesis
atorvastatin + valsartan	22	Atorvastatin will increase the level or effect of valsartan by Other (see comment). Use Caution/Monitor. The results from an in vitro study with human liver tissue indicate that valsartan is a substrate of the hepatic uptake transporter OATP1B1; co-administration with OATP1B1 inhibitors may increase valsartan systemic exposure
captopril + potassium	22	Captopril increases levels of potassium chloride by decreasing elimination. Use Caution/ Monitor. Risk of hyperkalemia
indapamide + losartan	21	Losartan increases and indapamide decreases serum potassium. Effect of interaction is not clear, use caution. Use Caution/Monitor
candesartan + metoprolol	20	Candesartan and metoprolol both increase serum potassium. Use Caution/Monitor
clopidogrel + rivaroxaban	19	Rivaroxaban, clopidogrel. Other (see comment). Use Caution/Monitor. Comment: Avoid concurrent administration of clopidogrel with rivaroxaban unless the benefit outweighs the risk of increased bleeding
digoxin + spironolactone	18	Spironolactone increases levels of digoxin by Other (see comment). Use Caution/Monitor. Comment: Spironolactone may cause false elevation of digoxin assay
digoxin + torsemide	18	Digoxin increases and torsemide decreases serum potassium. Effect of interaction is not clear, use caution. Use Caution/Monitor
hydrochlorothiazide + metoprolol	18	Hydrochlorothiazide, metoprolol. Either increases toxicity of the other by Other (see comment). Modify Therapy/Monitor Closely. Comment: May cause idiosyncratic reaction, resulting in acute transient myopia and acute angle-closure glaucoma, which can lead to permanent vision loss
captopril + indapamide	16	Indapamide, captopril. Either increases effects of the other by pharmacodynamic synergism. Use Caution/Monitor. Both drugs lower blood pressure. Increased risk of nephrotoxicity. Monitor blood pressure and renal function
aspirin + potassium	15	Aspirin and potassium acid phosphate both increase serum potassium. Modify Therapy/Monitor Closely
aspirin + telmisartan	15	Aspirin decreases effects of telmisartan by pharmacodynamic antagonism.

		Modify Therapy/Monitor Closely. NSAIDs decrease synthesis of vasodilating renal prostaglandins, and thus affect fluid homeostasis and may diminish antihypertensive effect
metoprolol + spironolactone	15	Metoprolol and spironolactone both increase serum potassium. Modify Therapy/Monitor Closely
metoprolol + torsemide	15	Metoprolol increases and torsemide decreases serum potassium. Effect of interaction is not clear, use caution. Use Caution/Monitor
amiodarone + captopril	14	Amiodarone, captopril. Either increases effects of the other by pharmacodynamic synergism. Use Caution/Monitor. Both drugs lower blood pressure. Monitor blood pressure
amlodipine + metformin	14	Amlodipine decreases effects of metformin by pharmacodynamic antagonism. Use Caution/Monitor. Patient should be closely observed for loss of blood glucose control; when drugs are withdrawn from a patient receiving metformin, patient should be observed closely for hypoglycemia
bisoprolol + candesartan	14	Bisoprolol, candesartan. Mechanism: pharmacodynamic synergism. Use Caution/Monitor. Risk of fetal compromise if given during pregnancy
bisoprolol + digoxin	14	Bisoprolol increases effects of digoxin by pharmacodynamic synergism. Use Caution/Monitor. Enhanced bradycardia
bisoprolol + valsartan	14	Bisoprolol, valsartan. Mechanism: pharmacodynamic synergism. Use Caution/Monitor. Risk of fetal compromise if given during pregnancy
metformin + torsemide	14	Torsemide decreases effects of metformin by pharmacodynamic antagonism. Use Caution/Monitor
amiodarone + warfarin	13	Amiodarone will increase the level or effect of warfarin by affecting hepatic enzyme CYP2C9/10 metabolism. Modify Therapy/Monitor Closely. Co-administration increases INR by 100% after 3-4 days. Reduce warfarin dose by one-third to one-half and monitor INR
aspirin + hydrochlorothiazide	13	Aspirin increases and hydrochlorothiazide decreases serum potassium. Effect of interaction is not clear, use caution. Use Caution/Monitor
aspirin + insulin	13	Aspirin increases effects of insulin aspart by pharmacodynamic synergism. Modify Therapy/Monitor Closely. Co-administration of insulin with high doses of salicylates (3 g/day or more) may increase risk for hypoglycemia. Insulin dose adjustment and increased frequency of glucose monitoring may be required
aspirin + olmesartan	12	Aspirin decreases effects of olmesartan by pharmacodynamic antagonism. Modify Therapy/ Monitor Closely. NSAIDs decrease synthesis of vasodilating renal prostaglandins, and thus affect fluid homeostasis and may diminish antihypertensive effect
aspirin + ticagrelor	12	Aspirin, ticagrelor. Other (see comment). Use Caution/Monitor. Comment: Maintenance doses of aspirin above 100 mg decreases effectiveness of ticagrelor. Therefore, after the initial loading dose of aspirin (usually 325 mg), use ticagrelor with a maintenance dose of aspirin of 75-100 mg
atorvastatin + ranolazine	12	Ranolazine will increase the level or effect of atorvastatin by P-glycoprotein (MDR1) efflux transporter. Use Caution/Monitor

losartan + nebivolol	12	Losartan and nebivolol both increase serum potassium. Use Caution/Monitor
bisoprolol + potassium	11	Bisoprolol and potassium chloride both increase serum potassium. Modify Therapy/ Monitor Closely
perindopril + torsemide	11	Perindopril, torsemide. Mechanism: pharmacodynamic synergism. Use Caution/Monitor. Risk of acute hypotension, renal insufficiency
aspirin + azilsartan	10	Aspirin, azilsartan. Either increases toxicity of the other by Other (see comment). Use Caution/Monitor. Comment: May result in renal function deterioration, particularly in elderly or volume depleted individuals. aspirin decreases effects of azilsartan by pharmacodynamic antagonism. Modify Therapy/Monitor Closely. NSAIDs decrease synthesis of vasodilating renal prostaglandins, and thus affect fluid homeostasis and may diminish antihypertensive effect
clopidogrel + ticagrelor	10	Ticagrelor, clopidogrel. Either increases effects of the other by Other (see comment). Use Caution/ Monitor. Comment: Increased risk of bleeding during concomitant use of medications that increase potential for bleeding
sotalol + torsemide	10	Sotalol increases and torsemide decreases serum potassium. Effect of interaction is not clear, use caution. Use Caution/Monitor
amiodarone + bisoprolol	9	Amiodarone, bisoprolol. Mechanism: pharmacodynamic synergism. Use Caution/Monitor. Risk of cardiotoxicity with bradycardia
amiodarone + rivaroxaban	9	Amiodarone increases levels of rivaroxaban by affecting hepatic/intestinal enzyme CYP3A4 metabolism. Use Caution/Monitor. Patients with renal impairment receiving rivaroxaban with drugs that are combined P-gp and weak or moderate CYP3A4 inhibitors may have significant increases in exposure compared with patients with normal renal function and no inhibitor use, since both pathways of rivaroxaban elimination are affected. Since these increases may increase bleeding risk, use rivaroxaban in this situation only if the potential benefit justifies the potential risk
amlodipine + carvedilol	9	Carvedilol and amlodipine both increase anti-hypertensive channel blocking. Modify Therapy/Monitor Closely
fosinopril + torsemide	9	Fosinopril, torsemide. Mechanism: pharmacodynamic synergism. Use Caution/Monitor. Risk of acute hypotension, renal insufficiency
furosemide + spironolactone	9	Spironolactone increases and furosemide decreases serum potassium. Effect of interaction is not clear, use caution. Modify Therapy/Monitor Closely
amlodipine + sotalol	8	Sotalol and amlodipine both increase anti-hypertensive channel blocking. Modify Therapy/Monitor Closely
aspirin + nebivolol	8	Aspirin decreases effects of nebivolol by pharmacodynamic antagonism. Use Caution/ Monitor. Long term (>1 wk) NSAID use. NSAIDs decrease prostaglandin synthesis
aspirin + prasugrel	8	Aspirin, prasugrel. Either increases toxicity of the other by pharmacodynamic synergism. Use Caution/ Monitor. The need for simultaneous use of low-dose aspirin and anticoagulant or antiplatelet agents are common for patients with cardiovascular disease; monitor closely
aspirin + rivaroxaban	8	Aspirin, rivaroxaban. Either increases toxicity of the other by anticoagulation.

		Use Caution/Monitor. Both drugs have the potential to cause bleeding. The need for simultaneous use of low-dose aspirin (<100 mg/day) with anticoagulants are common for patients with cardiovascular disease, but may result in increased bleeding; monitor closely. Promptly evaluate any signs or symptoms of blood loss if treated concomitantly with low-dose aspirin. Avoid co-administration with chronic use of higher dose aspirin
atorvastatin + nifedipine	8	Nifedipine will increase the level or effect of atorvastatin by affecting hepatic/intestinal enzyme CYP3A4 metabolism. Use Caution/Monitor. nifedipine will decrease the level or effect of atorvastatin by P-glycoprotein (MDR1) efflux transporter. Use Caution/Monitor
bisoprolol + formoterol	8	Bisoprolol decreases effects of formoterol by pharmacodynamic antagonism. Use Caution/Monitor
bisoprolol + telmisartan	8	Bisoprolol, telmisartan. Mechanism: pharmacodynamic synergism. Use Caution/Monitor. Risk of fetal compromise if given during pregnancy
candesartan + sotalol	8	Candesartan and sotalol both increase serum potassium. Use Caution/Monitor
candesartan + spironolactone	8	Candesartan and spironolactone both increase serum potassium. Modify Therapy/Monitor Closely
clopidogrel + felodipine	8	Felodipine decreases effects of clopidogrel by decreasing metabolism. Use Caution/Monitor. Cytochrome P450 2C19 inhibitors decrease the conversion of clopidogrel to its active form
indapamide + torsemide	8	Torsemide and indapamide both decrease serum potassium. Use Caution/Monitor
lisinopril + metformin	8	Lisinopril increases toxicity of metformin by unspecified interaction mechanism. Use Caution/Monitor. Increases risk for hypoglycemia and lactic acidosis
meloxicam + perindopril	8	Meloxicam, perindopril. pharmacodynamic antagonism. Avoid or Use Alternate Drug. Co-administration may result in a significant decrease in renal function. NSAIDs may diminish the antihypertensive effect of ACE inhibitors. The mechanism of these interactions is likely related to the ability of NSAIDs to reduce the synthesis of vasodilating renal prostaglandins
metoprolol + olmesartan	8	Metoprolol, olmesartan. Mechanism: pharmacodynamic synergism. Use Caution/ Monitor. Risk of fetal compromise if given during pregnancy
sotalol + spironolactone	8	Sotalol and spironolactone both increase serum potassium. Modify Therapy/Monitor Closely
aspirin + enalapril*	7	Aspirin, enalapril. Pharmacodynamic antagonism. Avoid or Use Alternate Drug. Co-administration may result in a significant decrease in renal function. NSAIDs may diminish the antihypertensive effect of ACE inhibitors. The mechanism of these interactions is likely related to the ability of NSAIDs to reduce the synthesis of vasodilating renal prostaglandins
aspirin + fosinopril	7	Aspirin, fosinopril. Pharmacodynamic antagonism. Avoid or Use Alternate Drug. Co-administration may result in a significant decrease in renal function. NSAIDs may diminish the antihypertensive effect of ACE inhibitors. The mechanism of these interactions is likely related to the ability of NSAIDs to

		reduce the synthesis of vasodilating renal prostaglandins
atorvastatin + dabigatran	7	Atorvastatin will increase the level or effect of dabigatran by P-glycoprotein (MDR1) efflux transporter. Use Caution/Monitor. Atrial fibrillation: Avoid co-administering dabigatran with P-gp inhibitors if CrCl <30 mL/min. DVT/PE treatment: Avoid co-administering dabigatran with P-gp inhibitors if CrCl <50 mL/min
bisoprolol + felodipine	7	Bisoprolol and felodipine both increase anti-hypertensive channel blocking. Modify Therapy/Monitor Closely
bisoprolol + nifedipine	7	Bisoprolol, nifedipine. Either decreases effects of the other by pharmacodynamic synergism. Use Caution/Monitor. Both drugs lower blood pressure
candesartan + torsemide	7	Candesartan increases and torsemide decreases serum potassium. Effect of interaction is not clear, use caution. Use Caution/Monitor
captopril + isosorbide di/mononitrate	7	Isosorbide mononitrate, captopril. Either increases effects of the other by pharmacodynamic synergism. Use Caution/ Monitor. Both drugs lower blood pressure. Monitor blood pressure. isosorbide dinitrate, captopril. Either increases effects of the other by pharmacodynamic synergism. Use Caution/ Monitor. Both drugs lower blood pressure. Monitor blood pressure
captopril + metformin	7	Captopril increases toxicity of metformin by unspecified interaction mechanism. Use Caution/ Monitor. Increases risk for hypoglycemia and lactic acidosis
clopidogrel + pantoprazole	7	Pantoprazole decreases effects of clopidogrel by affecting hepatic enzyme CYP2C19 metabolism. Use Caution/Monitor. Clopidogrel efficacy may be reduced by drugs that inhibit CYP2C19. Inhibition of platelet aggregation by clopidogrel is entirely due to the active clopidogrel metabolite. Clopidogrel is metabolized in part by CYP2C19. Pantoprazole prescribing information state that co-administration with clopidogrel had no clinically important effect on exposure to clopidogrel active metabolite; no dose adjustment of clopidogrel is required
losartan + potassium	7	Losartan and potassium chloride both increase serum potassium. Use Caution/Monitor
rosuvastatin + warfarin	7	Rosuvastatin increases effects of warfarin by anticoagulation. Use Caution/Monitor
amiodarone + carvedilol	6	Amiodarone will increase the level or effect of carvedilol by affecting hepatic enzyme CYP2C9/ 10 metabolism. Use Caution/Monitor. amiodarone will increase the level or effect of carvedilol by affecting hepatic enzyme CYP2D6 metabolism. Use Caution/ Monitor. Monitor for signs of bradycardia or heart block when amiodarone and a beta adrenergic blocker are co-administered. Amiodarone should be used with caution in patients receiving a beta adrenergic blocker, particularly if there is suspicion of underlying dysfunction of the sinus node, such as bradycardia or sick sinus syndrome, or if there is partial AV block
aspirin + furosemide	6	Aspirin increases and furosemide decreases serum potassium. Effect of interaction is not clear, use caution. Use Caution/Monitor
bisoprolol + hydrochlorothiazide	6	Bisoprolol increases and hydrochlorothiazide decreases serum potassium.

		Effect of interaction is not clear, use caution. Use Caution/Monitor
carvedilol + losartan	6	Carvedilol, losartan. Mechanism: pharmacodynamic synergism. Use Caution/Monitor. Risk of fetal compromise if given during pregnancy
digoxin + losartan	6	Losartan and digoxin both increase serum potassium. Use Caution/Monitor
indapamide + meloxicam	6	Indapamide will increase the level or effect of meloxicam by acidic (anionic) drug competition for renal tubular clearance. Minor/ Significance Unknown
indapamide + potassium	6	Potassium chloride increases and indapamide decreases serum potassium. Effect of interaction is not clear, use caution. Modify Therapy/ Monitor Closely
indapamide + spironolactone	6	Spironolactone increases and indapamide decreases serum potassium. Effect of interaction is not clear, use caution. Modify Therapy/Monitor Closely
insulin + losartan	6	Losartan increases effects of insulin regular human by unspecified interaction mechanism. Use Caution/ Monitor. Concomitant use of insulin and ARBs may require insulin dosage adjustment and increased glucose monitoring
omeprazole + warfarin	6	Omeprazole will increase the level or effect of warfarin by Other (see comment). Use Caution/Monitor. Warfarin's less potent R-enantiomer is metabolized in part by CYP3A4 (and also CYP1A2 and CYP2C19). Monitor INR more frequently if co-administered with inhibitors of these isoenzymes and adjust warfarin dose if needed
perindopril + spironolactone	6	Perindopril, spironolactone. Mechanism: pharmacodynamic synergism. Use Caution/Monitor. Risk of hyperkalemia
potassium + valsartan	6	Valsartan and potassium chloride both increase serum potassium. Use Caution/Monitor
sotalol + telmisartan	6	Sotalol, telmisartan. Mechanism: pharmacodynamic synergism. Use Caution/Monitor. Risk of fetal compromise if given during pregnancy
amiodarone + pentoxifylline	5	Amiodarone will increase the level or effect of pentoxifylline by affecting hepatic enzyme CYP1A2 metabolism. Use Caution/ Monitor
amlodipine + magnesium	5	Magnesium supplement, amlodipine. Either increases toxicity of the other by pharmacodynamic synergism. Use Caution/Monitor. Calcium channel blockers may increase toxic effects of magnesium; magnesium may increase hypotensive effects of calcium channel blockers
aspirin + warfarin	5	Aspirin increases effects of warfarin by anticoagulation. Modify Therapy/Monitor Closely. Avoid co-administration of chronic high-dose aspirin. Aspirin's antiplatelet properties may increase anticoagulation effect of warfarin. The need for simultaneous use of low-dose aspirin and warfarin is common for patients with cardiovascular disease
atorvastatin + telmisartan	5	Telmisartan increases toxicity of atorvastatin by Other (see comment). Use Caution/Monitor. Comment: OATP1B1 inhibitors may increase risk of myopathy
bisoprolol + furosemide	5	Bisoprolol increases and furosemide decreases serum potassium. Effect of interaction is not clear, use caution. Use Caution/Monitor
carvedilol + felodipine	5	Carvedilol and felodipine both increase anti-hypertensive channel blocking. Modify Therapy/ Monitor Closely

fosinopril + spironolactone	5	Fosinopril, spironolactone. Mechanism: pharmacodynamic synergism. Use Caution/Monitor. Risk of hyperkalemia
hydrochlorothiazide + olmesartan	5	Olmesartan increases and hydrochlorothiazide decreases serum potassium. Effect of interaction is not clear, use caution. Use Caution/ Monitor
losartan + warfarin	5	Losartan will increase the level or effect of warfarin by affecting hepatic enzyme CYP2C9/10 metabolism. Use Caution/Monitor
perindopril + potassium	5	Perindopril increases levels of potassium chloride by decreasing elimination. Use Caution/ Monitor. Risk of hyperkalemia
aspirin + digoxin	4	Aspirin and digoxin both increase serum potassium. Use Caution/Monitor
aspirin + ibuprofen	4	Aspirin and ibuprofen both increase anticoagulation. Use Caution/Monitor. aspirin and ibuprofen both increase serum potassium. Use Caution/Monitor.
aspirin + meloxicam	4	Aspirin and meloxicam both increase anticoagulation. Use Caution/Monitor
aspirin + timolol	4	Aspirin decreases effects of timolol by pharmacodynamic antagonism. Use Caution/ Monitor. Long term (>1 wk) NSAID use. NSAIDs decrease prostaglandin synthesis
atorvastatin + budesonide	4	Atorvastatin will increase the level or effect of budesonide by P-glycoprotein (MDR1) efflux transporter. Use Caution/Monitor
atorvastatin + digoxin	4	Atorvastatin will increase the level or effect of digoxin by P-glycoprotein (MDR1) efflux transporter. Use Caution/Monitor
atorvastatin + verapamil	4	Verapamil will increase the level or effect of atorvastatin by affecting hepatic/ intestinal enzyme CYP3A4 metabolism. Use Caution/ Monitor. verapamil will increase the level or effect of atorvastatin by P-glycoprotein (MDR1) efflux transporter. Use Caution/ Monitor
captopril + chlorthalidone	4	Captopril, chlorthalidone. Either increases toxicity of the other by pharmacodynamic synergism. Use Caution/Monitor. Both drugs lower blood pressure. Increased risk of nephrotoxicity. Monitor blood pressure and renal function
digoxin + metformin	4	Digoxin, metformin. Either increases levels of the other by basic (cationic) drug competition for renal tubular clearance. Use Caution/Monitor. Measure serum digoxin concentrations before initiating metformin. Monitor patients who take both metformin and digoxin for possible digoxin toxicity and lactic acidosis. Reduce the digoxin and/or metformin dose as necessary
digoxin + metoprolol	4	Digoxin increases toxicity of metoprolol by unspecified interaction mechanism. Avoid or Use Alternate Drug. Can increase risk of bradycardia
ferrous sulfate + omeprazole	4	Omeprazole will decrease the level or effect of ferrous sulfate by increasing gastric pH. Applies only to oral form of both agents. Use Caution/Monitor
formoterol + torsemide	4	Formoterol and torsemide both decrease serum potassium. Use Caution/Monitor
furosemide + losartan	4	Losartan increases and furosemide decreases serum potassium. Effect of interaction is not clear, use caution. Use Caution/Monitor
ibuprofen + meloxicam	4	Ibuprofen and meloxicam both increase serum potassium. Use Caution/Monitor. ibuprofen and meloxicam both increase anticoagulation. Use

		Caution/Monitor
ketoprofen + meloxicam	4	Ketoprofen and meloxicam both increase anticoagulation. Use Caution/Monitor. ketoprofen and meloxicam both increase serum potassium. Use Caution/Monitor
losartan + sotalol	4	Losartan and sotalol both increase serum potassium. Use Caution/Monitor
metoprolol + valsartan	4	Metoprolol, valsartan. Mechanism: pharmacodynamic synergism. Use Caution/Monitor. Risk of fetal compromise if given during pregnancy
nebivolol + spironolactone	4	Nebivolol and spironolactone both increase serum potassium. Modify Therapy/Monitor Closely
sotalol + valsartan	4	Sotalol, valsartan. Mechanism: pharmacodynamic synergism. Use Caution/ Monitor. Risk of fetal compromise if given during pregnancy
spironolactone + telmisartan	4	Telmisartan and spironolactone both increase serum potassium. Modify Therapy/Monitor Closely
telmisartan + torsemide	4	Telmisartan increases and torsemide decreases serum potassium. Effect of interaction is not clear, use caution. Use Caution/Monitor
apixaban + aspirin	3	Aspirin and apixaban both increase anticoagulation. Modify Therapy/Monitor Closely. Both drugs have the potential to cause bleeding. The need for simultaneous use of low-dose aspirin (<100 mg/day) with anticoagulants are common for patients with cardiovascular disease, but may result in increased bleeding; monitor closely. Promptly evaluate any signs or symptoms of blood loss if treated concomitantly with low-dose aspiriin. Avoid co-administration with chronic use of higher dose aspirin. In 1 trial (APPRAISE-2), therapy was terminated because of significantly increased bleeding when apixaban was administered with dual antiplatelet therapy (eg, aspirin plus clopidogrel) compared with single antiplatelet treatment
aspirin + chlorthalidone	3	Aspirin increases and chlorthalidone decreases serum potassium. Effect of interaction is not clear, use caution. Use Caution/Monitor
aspirin + formoterol	3	Aspirin increases and formoterol decreases serum potassium. Effect of interaction is not clear, use caution. Use Caution/Monitor
bisoprolol + chlorthalidone	3	Bisoprolol increases and chlorthalidone decreases serum potassium. Effect of interaction is not clear, use caution. Use Caution/Monitor
candesartan + meloxicam	3	Candesartan, meloxicam. Either increases toxicity of the other by Other (see comment). Use Caution/ Monitor. Comment: May result in renal function deterioration, particularly in elderly or volume depleted individuals
captopril + furosemide	3	Captopril, furosemide. Mechanism: pharmacodynamic synergism. Use Caution/Monitor. Risk of acute hypotension, renal insufficiency
carvedilol + torsemide	3	Carvedilol increases and torsemide decreases serum potassium. Effect of interaction is not clear, use caution. Use Caution/Monitor
dexamethasone + levofloxacin	3	Dexamethasone and levofloxacin both increase Other (see comment). Use Caution/Monitor. Co-administration of quinolone antibiotics and corticosteroids may increase risk of tendon rupture
diclofenac + losartan	3	Diclofenac decreases effects of losartan by pharmacodynamic antagonism.

		Modify Therapy/ Monitor Closely. NSAIDs decrease synthesis of vasodilating renal prostaglandins, and thus affect fluid homeostasis and may diminish antihypertensive effect
formoterol + salmeterol	3	Formoterol and salmeterol both decrease serum potassium. Use Caution/Monitor
formoterol + spironolactone	3	Spironolactone increases and formoterol decreases serum potassium. Effect of interaction is not clear, use caution. Modify Therapy/Monitor Closely
hydrochlorothiazide + sotalol	3	Sotalol increases and hydrochlorothiazide decreases serum potassium. Effect of interaction is not clear, use caution. Use Caution/Monitor
ibuprofen + losartan	3	Ibuprofen decreases effects of losartan by pharmacodynamic antagonism. Modify Therapy/ Monitor Closely. NSAIDs decrease synthesis of vasodilating renal prostaglandins, and thus affect fluid homeostasis and may diminish antihypertensive effect
indomethacin + losartan	3	Indomethacin decreases effects of losartan by pharmacodynamic antagonism. Modify Therapy/Monitor Closely. NSAIDs decrease synthesis of vasodilating renal prostaglandins, and thus affect fluid homeostasis and may diminish antihypertensive effect
insulin + metformin	3	Metformin, insulin aspart. Either increases effects of the other by pharmacodynamic synergism. Use Caution/Monitor. Antidiabetic agents are often used in combination; dosage adjustments may be required when initiating or discontinuing antidiabetic agents. metformin, insulin detemir. Either increases effects of the other by pharmacodynamic synergism. Use Caution/Monitor. Antidiabetic agents are often used in combination; dosage adjustments may be required when initiating or discontinuing antidiabetic agents
ipratropium + tiotropium	3	Ipratropium and tiotropium both decrease cholinergic effects/transmission. Use Caution/Monitor. Due to the poor systemic absorption of ipratropium, interaction unlikely at regularly recommended dosages
levothyroxine + metformin	3	Levothyroxine decreases effects of metformin by pharmacodynamic antagonism. Use Caution/Monitor. Patient should be closely observed for loss of blood glucose control; when drugs are withdrawn from a patient receiving metformin, patient should be observed closely for hypoglycemia
meloxicam + spironolactone	3	Spironolactone and meloxicam both increase serum potassium. Modify Therapy/Monitor Closely
metoprolol + nifedipine	3	Metoprolol and nifedipine both increase anti-hypertensive channel blocking. Modify Therapy/Monitor Closely
nebivolol + torsemide	3	Nebivolol increases and torsemide decreases serum potassium. Effect of interaction is not clear, use caution. Use Caution/Monitor
nifedipine + nitroglycerin	3	Nifedipine, nitroglycerin sublingual. Either increases toxicity of the other by additive vasodilation. Modify Therapy/ Monitor Closely. Marked orthostatic hypotension reported with concomitant use
aceclofenac + aspirin	2	Aceclofenac and aspirin both increase anticoagulation. Use Caution/Monitor. aceclofenac and aspirin both increase serum potassium. Use Caution/Monitor
aceclofenac + bisoprolol	2	Aceclofenac decreases effects of bisoprolol by pharmacodynamic antagonism.

		Use Caution/Monitor. Long term (>1 wk) NSAID use. NSAIDs decrease prostaglandin synthesis
amiodarone + budesonide	2	Amiodarone will increase the level or effect of budesonide by P-glycoprotein (MDR1) efflux transporter. Use Caution/Monitor
amiodarone + dabigatran	2	Amiodarone will increase the level or effect of dabigatran by P-glycoprotein (MDR1) efflux transporter. Use Caution/ Monitor. Atrial fibrillation: Avoid co-administering dabigatran with P-gp inhibitors if CrCl <30 mL/ min. DVT/ PE treatment: Avoid co-administering dabigatran with P-gp inhibitors if CrCl <50 mL/ min
amiodarone + hydrochlorothiazide	2	Amiodarone will increase the level or effect of hydrochlorothiazide by basic (cationic) drug competition for renal tubular clearance. Use Caution/Monitor
amiodarone + metformin	2	Amiodarone will increase the level or effect of metformin by basic (cationic) drug competition for renal tubular clearance. Use Caution/Monitor
amlodipine + nifedipine	2	Amlodipine and nifedipine both increase anti-hypertensive channel blocking. Use Caution/Monitor
aspirin + doxazosin	2	Aspirin decreases effects of doxazosin by pharmacodynamic antagonism. Use Caution/Monitor. NSAIDs decrease prostaglandin synthesis
aspirin + enoxaparin	2	Aspirin, enoxaparin. Either increases toxicity of the other by pharmacodynamic synergism. Use Caution/Monitor. The need for simultaneous use of low-dose aspirin and anticoagulant or antiplatelet agents are common for patients with cardiovascular disease; monitor closely
aspirin + heparin	2	Aspirin, heparin. Either increases toxicity of the other by anticoagulation. Use Caution/Monitor. The need for simultaneous use of low-dose aspirin and anticoagulant or antiplatelet agents are common for patients with cardiovascular disease; monitor closely
aspirin + ketoprofen	2	Aspirin and ketoprofen both increase anticoagulation. Use Caution/Monitor. aspirin and ketoprofen both increase serum potassium. Use Caution/Monitor. aspirin will increase the level or effect of ketoprofen by acidic (anionic) drug competition for renal tubular clearance. Minor/Significance Unknown
aspirin + ramipril	2	Aspirin, ramipril. pharmacodynamic antagonism. Avoid or Use Alternate Drug. Co-administration may result in a significant decrease in renal function. NSAIDs may diminish the antihypertensive effect of ACE inhibitors. The mechanism of these interactions is likely related to the ability of NSAIDs to reduce the synthesis of vasodilating renal prostaglandins
azilsartan + meloxicam	2	Meloxicam, azilsartan. Either increases toxicity of the other by Other (see comment). Use Caution/Monitor. Comment: May result in renal function deterioration, particularly in elderly or volume depleted individuals. meloxicam decreases effects of azilsartan by pharmacodynamic antagonism. Modify Therapy/Monitor Closely. NSAIDs decrease synthesis of vasodilating renal prostaglandins, and thus affect fluid homeostasis and may diminish antihypertensive effect
bisoprolol + calcium carbonate	2	Calcium carbonate decreases levels of bisoprolol by inhibition of GI absorption. Applies only to oral form of both agents. Use Caution/Monitor. Separate by 2 hours

bisoprolol + ketoprofen	2	Bisoprolol and ketoprofen both increase serum potassium. Use Caution/Monitor
bisoprolol + lornoxicam	2	Bisoprolol and lornoxicam both increase serum potassium. Use Caution/Monitor
bisoprolol + meloxicam	2	Bisoprolol and meloxicam both increase serum potassium. Use Caution/Monitor
bisoprolol + nebivolol	2	Bisoprolol and nebivolol both increase anti-hypertensive channel blocking. Avoid or Use Alternate Drug
bisoprolol + timolol	2	Bisoprolol and timolol both increase anti-hypertensive channel blocking. Avoid or Use Alternate Drug
budesonide + omeprazole	2	Omeprazole decreases effects of budesonide by increasing gastric pH. Applies only to oral form of both agents. Modify Therapy/Monitor Closely. Enteric-coated budesonide dissolves at pH >5.5. Also, dissolution of extended-release budesonide tablets is pH dependent. Co-administration with drugs that increase gastric pH may cause these budesonide products to prematurely dissolve, and possibly affect release properties and absorption of the drug in the duodenum
calcium carbonate + carvedilol	2	Calcium carbonate decreases levels of carvedilol by inhibition of GI absorption. Applies only to oral form of both agents. Use Caution/ Monitor. Separate by 2 hours
calcium carbonate + metoprolol	2	Calcium carbonate decreases effects of metoprolol by unspecified interaction mechanism. Use Caution/Monitor
candesartan + eplerenone	2	Candesartan, eplerenone. Mechanism: pharmacodynamic synergism. Use Caution/Monitor. Risk of hyperkalemia
captopril + digoxin	2	Candesartan, eplerenone. Mechanism: pharmacodynamic synergism. Use Caution/ Monitor. Risk of hyperkalemia
captopril + eplerenone	2	Captopril, eplerenone. Either increases toxicity of the other by Mechanism: pharmacodynamic synergism. Use Caution/Monitor. Risk of hyperkalemia. Monitor potassium
captopril + hydrochlorothiazide	2	Captopril, hydrochlorothiazide. Either increases effects of the other by pharmacodynamic synergism. Use Caution/ Monitor. Both drugs lower blood pressure. Increased risk of nephrotoxicity. Monitor blood pressure and renal function
carvedilol + nifedipine	2	Carvedilol and nifedipine both increase anti-hypertensive channel blocking. Modify Therapy/Monitor Closely
celecoxib + meloxicam	2	Celecoxib and meloxicam both increase anticoagulation. Use Caution/Monitor. celecoxib and meloxicam both increase serum potassium. Use Caution/Monitor
ciprofloxacin + dexamethasone	2	Dexamethasone and ciprofloxacin both increase Other (see comment). Use Caution/Monitor. Co-administration of quinolone antibiotics and corticosteroids may increase risk of tendon rupture
clopidogrel + dabigatran	2	Dabigatran, clopidogrel. Either increases effects of the other by pharmacody-

		namic synergism. Use Caution/ Monitor. Both drugs have the potential to cause bleeding. Concomitant use may increase risk of bleeding
diclofenac + flurbiprofen	2	Diclofenac and flurbiprofen both increase anticoagulation. Use Caution/Monitor. diclofenac and flurbiprofen both increase serum potassium. Use Caution/Monitor
diclofenac + lornoxicam	2	Diclofenac and lornoxicam both increase anticoagulation. Use Caution/Monitor. diclofenac and lornoxicam both increase serum potassium. Use Caution/Monitor
diclofenac + meloxicam	2	Diclofenac and meloxicam both increase serum potassium. Use Caution/Monitor
diclofenac + spironolactone	2	Spironolactone and diclofenac both increase serum potassium. Modify Therapy/Monitor Closely
diclofenac + torsemide	2	Diclofenac increases and torsemide decreases serum potassium. Effect of interaction is not clear, use caution. Use Caution/Monitor
digoxin + pantoprazole	2	Pantoprazole will increase the level or effect of digoxin by increasing gastric pH. Applies only to oral form of both agents. Avoid or Use Alternate Drug
enalapril + metformin	2	Enalapril increases toxicity of metformin by unspecified interaction mechanism. Use Caution/Monitor. Increases risk for hypoglycemia and lactic acidosis
eplerenone + losartan	2	Losartan, eplerenone. Mechanism: pharmacodynamic synergism. Use Caution/Monitor. Risk of hyperkalemia
felodipine + nitroglycerin	2	Felodipine, nitroglycerin sublingual. Either increases toxicity of the other by additive vasodilation. Modify Therapy/Monitor Closely. Marked orthostatic hypotension reported with concomitant use
ferrous sulfate + pantoprazole	2	Pantoprazole will decrease the level or effect of ferrous sulfate by increasing gastric pH. Applies only to oral form of both agents. Use Caution/Monitor
indapamide + nebivolol	2	Nebivolol increases and indapamide decreases serum potassium. Effect of interaction is not clear, use caution. Use Caution/Monitor
indapamide + sotalol	2	Indapamide and sotalol both increase QTc interval. Contraindicated
indapamide + timolol	2	Timolol increases and indapamide decreases serum potassium. Effect of interaction is not clear, use caution. Use Caution/Monitor
meloxicam + naproxen	2	Meloxicam and naproxen both increase serum potassium. Use Caution/Monitor. meloxicam and naproxen both increase anticoagulation. Use Caution/Monitor
metformin + nifedipine	2	Nifedipine decreases effects of metformin by pharmacodynamic antagonism. Use Caution/Monitor. Patient should be closely observed for loss of blood glucose control; when drugs are withdrawn from a patient receiving metformin, patient should be observed closely for hypoglycemia. nifedipine increases levels of metformin by enhancing GI absorption. Applies only to oral form of both agents. Minor/Significance Unknown
metoprolol + ranolazine	2	Ranolazine will increase the level or effect of metoprolol by affecting hepatic enzyme CYP2D6 metabolism. Use Caution/Monitor

metoprolol + telmisartan	2	Metoprolol, telmisartan. Mechanism: pharmacodynamic synergism. Use Caution/Monitor. Risk of fetal compromise if given during pregnancy
nebivolol + potassium	2	Nebivolol and potassium chloride both increase serum potassium. Modify Therapy/Monitor Closely
phenylephrine + xylometazoline	2	Phenylephrine and xylometazoline both decrease sedation. Use Caution/Monitor. phenylephrine and xylometazoline both increase sympathetic (adrenergic) effects, including increased blood pressure and heart rate. Use Caution/Monitor
potassium + sotalol	2	Sotalol and potassium chloride both increase serum potassium. Modify Therapy/Monitor Closely
spironolactone + valsartan	2	Valsartan and spironolactone both increase serum potassium. Modify Therapy/Monitor Closely
torsemide + valsartan	2	Valsartan increases and torsemide decreases serum potassium. Effect of interaction is not clear, use caution. Use Caution/Monitor
diclofenac + indapamide	2	Diclofenac increases and indapamide decreases serum potassium. Effect of interaction is not clear, use caution. Use Caution/Monitor
aceclofenac + indapamide	1	Aceclofenac increases and indapamide decreases serum potassium. Effect of interaction is not clear, use caution. Use Caution/Monitor
acetaminophen + warfarin	1	Acetaminophen increases effects of warfarin by anticoagulation. Use Caution/Monitor
allopurinol + calcium carbonate	1	Calcium carbonate decreases levels of allopurinol by inhibition of GI absorption. Applies only to oral form of both agents. Use Caution/Monitor. Separate by 2 hours
amiodarone + atenolol	1	Amiodarone, atenolol. Mechanism: pharmacodynamic synergism. Use Caution/Monitor. Risk of cardiotoxicity with bradycardia
amiodarone + ciprofloxacin	1	Ciprofloxacin and amiodarone both increase QTc interval. Use Caution/Monitor. Ciprofloxacin elicits minimal effects on QT interval. Caution if used in combination with other drugs known to affect QT interval or in patients with other risk factors
amlodipine + carbamazepine	1	Carbamazepine will decrease the level or effect of amlodipine by affecting hepatic/intestinal enzyme CYP3A4 metabolism. Modify Therapy/Monitor Closely
atenolol + spironolactone	1	Atenolol and spironolactone both increase serum potassium. Modify Therapy/Monitor Closely
atorvastatin + carbamazepine	1	Carbamazepine will decrease the level or effect of atorvastatin by affecting hepatic/intestinal enzyme CYP3A4 metabolism. Avoid or Use Alternate Drug
bisoprolol + ivabradine	1	Ivabradine, bisoprolol. Either increases effects of the other by pharmacodynamic synergism. Modify Therapy/Monitor Closely. Most patients receiving ivabradine will also be treated with a beta-blocker. The risk of bradycardia increases with co-administration of drugs that slow heart rate (eg, digoxin, amiodarone, beta-blockers). Monitor heart rate in patients taking ivabradine with other negative chronotropes

bisoprolol + theophylline	1	Bisoprolol, theophylline. Other (see comment). Use Caution/Monitor. Comment: Beta blockers (esp. non selective) antagonize theophylline effects, while at the same time increasing theophylline levels and toxicity (mechanism: decreased theophylline metabolism). Smoking increases risk of interaction
budesonide + clarithromycin	1	Budesonide will decrease the level or effect of clarithromycin by affecting hepatic/intestinal enzyme CYP3A4 metabolism. Minor/Significance Unknown
budesonide + theophylline	1	Budesonide will decrease the level or effect of theophylline by affecting hepatic/intestinal enzyme CYP3A4 metabolism. Use Caution/Monitor
caffeine + phenylephrine	1	Caffeine and phenylephrine both decrease sedation. Use Caution/Monitor
calcium carbonate + celecoxib	1	Calcium carbonate decreases levels of celecoxib by inhibition of GI absorption. Applies only to oral form of both agents. Use Caution/Monitor. Separate by 2 hours
calcium carbonate + fosinopril	1	Calcium carbonate decreases effects of fosinopril by unspecified interaction mechanism. Use Caution/Monitor
calcium carbonate + levothyroxine	1	Calcium carbonate decreases levels of levothyroxine by inhibition of GI absorption. Applies only to oral form of both agents. Use Caution/Monitor. Separate by 2 hours
calcium carbonate + nifedipine	1	Calcium carbonate decreases effects of nifedipine by pharmacodynamic antagonism. Use Caution/Monitor
calcium carbonate + ramipril	1	Calcium carbonate decreases effects of ramipril by unspecified interaction mechanism. Use Caution/Monitor
calcium carbonate + rosuvastatin	1	Calcium carbonate decreases levels of rosuvastatin by inhibition of GI absorption. Applies only to oral form of both agents. Use Caution/Monitor. Separate by 2 hours
calcium carbonate + vitamin D	1	Vitamin D, calcium carbonate. Other (see comment). Use Caution/Monitor. Comment: The concurrent use of vitamin D with calcium salts is generally beneficial; in some patients this combination may result in hypercalcemia
candesartan + chlorthalidone	1	Candesartan increases and chlorthalidone decreases serum potassium. Effect of interaction is not clear, use caution. Use Caution/Monitor
candesartan + hydrochlorothiazide	1	Candesartan increases and hydrochlorothiazide decreases serum potassium. Effect of interaction is not clear, use caution. Use Caution/Monitor
candesartan + indapamide	1	Candesartan increases and indapamide decreases serum potassium. Effect of interaction is not clear, use caution. Use Caution/Monitor
candesartan + potassium	1	Candesartan and potassium chloride both increase serum potassium. Use Caution/Monitor
captopril + calcium carbonate	1	Calcium carbonate decreases effects of captopril by unspecified interaction mechanism. Use Caution/Monitor. Calcium carbonate may decrease absorption of captopril
captopril + ciprofloxacin	1	Captopril increases toxicity of ciprofloxacin by Mechanism: unspecified interaction mechanism. Use Caution/Monitor. ACE Inhibitors increase arrhythmogenic potential of ciprofloxacin. Monitor ECG and QT interval

captopril + ibuprofen	1	Captopril, ibuprofen. Either increases toxicity of the other by Other (see comment). Use Caution/Monitor. Comment: May result in renal function deterioration, particularly in elderly or volume depleted individuals
captopril + insulin	1	Captopril increases effects of insulin regular human by pharmacodynamic synergism. Use Caution/Monitor. Both drugs decrease blood glucose. Monitor blood glucose
captopril + ketoprofen	1	Captopril, ketoprofen. Either increases toxicity of the other by Other (see comment). Use Caution/Monitor. Comment: May result in renal function deterioration, particularly in elderly or volume depleted individuals
captopril + meloxicam	1	Captopril, meloxicam. Either increases toxicity of the other by Other (see comment). Use Caution/Monitor. Comment: May result in renal function deterioration, particularly in elderly or volume depleted individuals
captopril + pentoxifylline	1	Pentoxifylline, captopril. Either increases effects of the other by pharmacodynamic synergism. Use Caution/Monitor. Both drugs lower blood pressure. Monitor blood pressure
carbamazepine + warfarin	1	Carbamazepine will decrease the level or effect of warfarin by affecting hepatic enzyme CYP2C9/10 metabolism. Modify Therapy/Monitor Closely
carvedilol + hydrochlorothiazide	1	Carvedilol increases and hydrochlorothiazide decreases serum potassium. Effect of interaction is not clear, use caution. Use Caution/Monitor
carvedilol + indapamide	1	Carvedilol increases and indapamide decreases serum potassium. Effect of interaction is not clear, use caution. Use Caution/Monitor
carvedilol + potassium	1	Carvedilol and potassium chloride both increase serum potassium. Modify Therapy/Monitor Closely
celecoxib + heparin	1	Heparin and celecoxib both increase anticoagulation. Modify Therapy/Monitor Closely
celecoxib + indapamide	1	Celecoxib increases and indapamide decreases serum potassium. Effect of interaction is not clear, use caution. Use Caution/Monitor
celecoxib + lisinopril	1	Celecoxib, lisinopril. pharmacodynamic antagonism. Avoid or Use Alternate Drug. Co-administration may result in a significant decrease in renal function. NSAIDs may diminish the antihypertensive effect of ACE inhibitors. The mechanism of these interactions is likely related to the ability of NSAIDs to reduce the synthesis of vasodilating renal prostaglandins
celecoxib + rivaroxaban	1	Rivaroxaban, celecoxib. Other (see comment). Use Caution/Monitor. Comment: NSAIDs are known to increase bleeding. Bleeding risk may be increased when NSAIDs are used concomitantly with rivaroxaban. Monitor for signs/symptoms of blood loss
chloramphenicol + tinidazole	1	Chloramphenicol will increase the level or effect of tinidazole by affecting hepatic/intestinal enzyme CYP3A4 metabolism. Use Caution/Monitor
chlorpheniramine + caffeine	1	Chlorpheniramine increases and caffeine decreases sedation. Effect of interaction is not clear, use caution. Use Caution/Monitor
chlorpheniramine + phenylephrine	1	Chlorpheniramine increases and phenylephrine decreases sedation. Effect of interaction is not clear, use caution. Use Caution/Monitor

chlorthalidone + hydrochlorothiazide	1	Chlorthalidone and hydrochlorothiazide both decrease serum potassium. Use Caution/Monitor
chlorthalidone + meloxicam	1	Chlorthalidone will increase the level or effect of meloxicam by acidic (anionic) drug competition for renal tubular clearance. Minor/Significance Unknown
chlorthalidone + metoprolol	1	Metoprolol increases and chlorthalidone decreases serum potassium. Effect of interaction is not clear, use caution. Use Caution/Monitor
ciprofloxacin + ibuprofen	1	Ibuprofen, ciprofloxacin. Other (see comment). Modify Therapy/Monitor Closely. Comment: Mechanism: unknown. Increased risk of CNS stimulation and seizures with high doses of fluoroquinolones
ciprofloxacin + pentoxifylline	1	Ciprofloxacin will increase the level or effect of pentoxifylline by affecting hepatic enzyme CYP1A2 metabolism. Use Caution/Monitor
ciprofloxacin + tinidazole	1	Ciprofloxacin will increase the level or effect of tinidazole by affecting hepatic/intestinal enzyme CYP3A4 metabolism. Use Caution/Monitor
clopidogrel + diclofenac	1	Clopidogrel, diclofenac. Either increases effects of the other by pharmacodynamic synergism. Modify Therapy/Monitor Closely. Clopidogrel and NSAIDs both inhibit platelet aggregation
clopidogrel + enoxaparin	1	Enoxaparin, clopidogrel. Either increases effects of the other by pharmacodynamic synergism. Modify Therapy/Monitor Closely. Enhanced risk of hemorrhage; additive effects are intended when both drugs are prescribed as indicated for ACS
clopidogrel + ibuprofen	1	Clopidogrel, ibuprofen. Either increases effects of the other by pharmacodynamic synergism. Modify Therapy/Monitor Closely. Clopidogrel and NSAIDs both inhibit platelet aggregation
clopidogrel + meloxicam	1	Clopidogrel, meloxicam. Either increases effects of the other by pharmacodynamic synergism. Modify Therapy/Monitor Closely. Clopidogrel and NSAIDs both inhibit platelet aggregation
clopidogrel + piracetam	1	Piracetam increases effects of clopidogrel by pharmacodynamic synergism. Use Caution/Monitor
clopidogrel + ticagrelor	1	Ticagrelor, clopidogrel. Either increases effects of the other by Other (see comment). Use Caution/Monitor. Comment: Increased risk of bleeding during concomitant use of medications that increase potential for bleeding
clopidogrel + warfarin	1	Clopidogrel, warfarin. Either increases effects of the other by pharmacodynamic synergism. Modify Therapy/Monitor Closely. Drugs with antiplatelet properties may increase anticoagulation effect of warfarin
dabigatran + ibuprofen	1	Dabigatran and ibuprofen both increase anticoagulation. Use Caution/Monitor. Caution is advised, both drugs have the potential to cause bleeding. Concomitant use may increase risk of bleeding
dabigatran + rivaroxaban	1	Rivaroxaban, dabigatran. Either increases effects of the other by anticoagulation. Use Caution/Monitor. Avoid concurrent use of rivaroxaban with other anticoagulants due to increased bleeding risk other than during therapeutic transition periods where patients should be observed closely. Monitor for signs/symptoms of blood loss

diclofenac + levofloxacin	1	Levofloxacin, diclofenac. Other (see comment). Modify Therapy/Monitor Closely. Comment: Risk of CNS stimulation/seizure. Mechanism: Displacement of GABA from receptors in brain
diclofenac + perindopril	1	Diclofenac, perindopril. pharmacodynamic antagonism. Avoid or Use Alternate Drug. Co-administration may result in a significant decrease in renal function. NSAIDs may diminish the antihypertensive effect of ACE inhibitors. The mechanism of these interactions is likely related to the ability of NSAIDs to reduce the synthesis of vasodilating renal prostaglandins
diclofenac + rivaroxaban	1	Rivaroxaban, diclofenac. Other (see comment). Use Caution/Monitor. Comment: NSAIDs are known to increase bleeding. Bleeding risk may be increased when NSAIDs are used concomitantly with rivaroxaban. Monitor for signs/symptoms of blood loss
digoxin + esomeprazole	1	Esomeprazole will increase the level or effect of digoxin by increasing gastric pH. Applies only to oral form of both agents. Avoid or Use Alternate Drug
digoxin + lisinopril	1	Lisinopril increases levels of digoxin by unspecified interaction mechanism. Use Caution/Monitor
digoxin + omeprazole	1	Omeprazole will increase the level or effect of digoxin by increasing gastric pH. Applies only to oral form of both agents. Avoid or Use Alternate Drug
digoxin + valsartan	1	Valsartan will increase the level or effect of digoxin by decreasing renal clearance. Use Caution/Monitor. Monitor digoxin levels closely when co-administered with drugs that may decrease glomerular filtration or tubular secretion
diltiazem + magnesium	1	Magnesium supplement, diltiazem. Either increases toxicity of the other by pharmacodynamic synergism. Use Caution/Monitor. Calcium channel blockers may increase toxic effects of magnesium; magnesium may increase hypotensive effects of calcium channel blockers
doxazosin + bisoprolol	1	Doxazosin and bisoprolol both increase anti-hypertensive channel blocking. Modify Therapy/Monitor Closely
doxazosin + metoprolol	1	Doxazosin and metoprolol both increase anti-hypertensive channel blocking. Modify Therapy/Monitor Closely
doxazosin + nifedipine	1	Doxazosin and nifedipine both increase anti-hypertensive channel blocking. Use Caution/Monitor
enalapril + enoxaparin	1	Enoxaparin increases toxicity of enalapril by Other (see comment). Use Caution/Monitor. Comment: Low molecular weight heparins may suppress adrenal aldosterone secretion, which can potentially cause hyperkalemia
enalapril + insulin	1	Enalapril increases effects of insulin lispro by pharmacodynamic synergism. Use Caution/Monitor
enalapril + torsemide	1	Enalapril, torsemide. Mechanism: pharmacodynamic synergism. Use Caution/Monitor. Risk of acute hypotension, renal insufficiency
eplerenone + ramipril	1	Ramipril, eplerenone. Mechanism: pharmacodynamic synergism. Use Caution/Monitor. Risk of hyperkalemia
felodipine + metoprolol	1	Metoprolol and felodipine both increase anti-hypertensive channel blocking.

		Modify Therapy/Monitor Closely
formoterol + furosemide	1	Formoterol and furosemide both decrease serum potassium. Use Caution/Monitor
furosemide + indapamide	1	Furosemide and indapamide both decrease serum potassium. Use Caution/Monitor
furosemide + meloxicam	1	Meloxicam increases and furosemide decreases serum potassium. Effect of interaction is not clear, use caution. Use Caution/Monitor
furosemide + torsemide	1	Furosemide and torsemide both decrease serum potassium. Use Caution/Monitor
heparin + ketorolac	1	Heparin and ketorolac both increase anticoagulation. Modify Therapy/Monitor Closely
heparin + meloxicam	1	Heparin and meloxicam both increase anticoagulation. Modify Therapy/Monitor Closely
hydrochlorothiazide + telmisartan	1	Telmisartan increases and hydrochlorothiazide decreases serum potassium. Effect of interaction is not clear, use caution. Use Caution/Monitor
hydrochlorothiazide + valsartan	1	Valsartan increases and hydrochlorothiazide decreases serum potassium. Effect of interaction is not clear, use caution. Use Caution/Monitor
ibuprofen + indapamide	1	Ibuprofen increases and indapamide decreases serum potassium. Effect of interaction is not clear, use caution. Use Caution/Monitor
ibuprofen + lisinopril	1	ibuprofen, lisinopril. pharmacodynamic antagonism. Avoid or Use Alternate Drug. Co-administration may result in a significant decrease in renal function. NSAIDs may diminish the antihypertensive effect of ACE inhibitors. The mechanism of these interactions is likely related to the ability of NSAIDs to reduce the synthesis of vasodilating renal prostaglandins
ibuprofen + prednisolone	1	Ibuprofen, prednisolone. Either increases toxicity of the other by pharmacodynamic synergism. Use Caution/Monitor. Increased risk of GI ulceration
indapamide + valsartan	1	Valsartan increases and indapamide decreases serum potassium. Effect of interaction is not clear, use caution. Use Caution/Monitor
interferon alfa 2b + warfarin	1	Interferon alfa 2b increases effects of warfarin by unspecified interaction mechanism. Use Caution/Monitor
ivabradine + metoprolol	1	Ivabradine, metoprolol. Either increases effects of the other by pharmacodynamic synergism. Modify Therapy/Monitor Closely. Most patients receiving ivabradine will also be treated with a beta-blocker. The risk of bradycardia increases with co-administration of drugs that slow heart rate (eg, digoxin, amiodarone, beta-blockers). Monitor heart rate in patients taking ivabradine with other negative chronotropes
ketoprofen + ramipril	1	Ketoprofen, ramipril. pharmacodynamic antagonism. Avoid or Use Alternate Drug. Co-administration may result in a significant decrease in renal function. NSAIDs may diminish the antihypertensive effect of ACE inhibitors. The mechanism of these interactions is likely related to the ability of NSAIDs to reduce the synthesis of vasodilating renal prostaglandins
lisinopril + potassium	1	Lisinopril increases levels of potassium chloride by decreasing elimination.

		Use Caution/Monitor. Risk of hyperkalemia
loratadine + metronidazole	1	Metronidazole will increase the level or effect of loratadine by affecting hepatic/intestinal enzyme CYP3A4 metabolism. Use Caution/Monitor
loratadine + nifedipine	1	Nifedipine will increase the level or effect of loratadine by affecting hepatic/intestinal enzyme CYP3A4 metabolism. Use Caution/Monitor
lornoxicam + losartan	1	Losartan and lornoxicam both increase serum potassium. Use Caution/Monitor
losartan + tizanidine	1	Tizanidine increases effects of losartan by pharmacodynamic synergism. Use Caution/Monitor. Risk of hypotension
magnesium + nifedipine	1	Magnesium supplement, nifedipine. Either increases toxicity of the other by Other (see comment). Use Caution/Monitor. Comment: Calcium channel blockers may increase toxic effects of magnesium; magnesium may increase hypotensive effects of calcium channel blockers
magnesium + potassium	1	Magnesium supplement will decrease the level or effect of potassium phosphate by Other (see comment). Modify Therapy/Monitor Closely. Drug may form a chelate with divalent cations; may decrease absorption by the intestinal tract; applies to oral forms; separate administration of drugs to avoid interaction
meloxicam + torsemide	1	Meloxicam increases and torsemide decreases serum potassium. Effect of interaction is not clear, use caution. Use Caution/Monitor
metformin + ramipril	1	Ramipril increases toxicity of metformin by unspecified interaction mechanism. Use Caution/Monitor. Increases risk for hypoglycemia and lactic acidosis
metoprolol + potassium	1	Metoprolol and potassium chloride both increase serum potassium. Modify Therapy/Monitor Closely
metoprolol + propafenone	1	Propafenone will increase the level or effect of metoprolol by affecting hepatic enzyme CYP2D6 metabolism. Use Caution/Monitor. If concurrent therapy is required, monitor cardiac function carefully, particularly blood pressure. A dosage adjustment for the beta blocker may be required
nitroglycerin + verapamil	1	Verapamil, nitroglycerin sublingual. Either increases toxicity of the other by additive vasodilation. Modify Therapy/Monitor Closely. Marked orthostatic hypotension reported with concomitant use
olodaterol inhaled + torsemide	1	Torsemide and olodaterol inhaled both decrease serum potassium. Use Caution/Monitor
pitavastatin + telmisartan	1	Telmisartan increases toxicity of pitavastatin by Other (see comment). Use Caution/Monitor. Comment: OATP1B1 inhibitors may increase risk of myopathy
potassium + torsemide	1	Potassium chloride increases and torsemide decreases serum potassium. Effect of interaction is not clear, use caution. Modify Therapy/Monitor Closely
potassium + indapamide	1	Potassium chloride increases and indapamide decreases serum potassium. Effect of interaction is not clear, use caution. Modify Therapy/Monitor Closely
tiotropium + umeclidinium bro-	1	Umeclidinium bromide and tiotropium both decrease cholinergic ef-

mide	fects/transmission. Use Caution/Monitor. If possible, avoid co-administration of additional anticholinergic agents

***Note:** Impact of drug-drug interactions associated with the combinations 'aspirin + captopril' and 'aspirin + enalapril' may be considered insignificant due to the use of low-dose aspirin in the majority of cases. Administration of aspirin at doses less than 300 mg per day has little effect on the effectiveness of captopril and enalapril. Administration of aspirin in higher doses reduces the effectiveness of captopril and enalapril.

Table S7. The detailed explanation of the entire list of pairwise minor drug-drug interactions among prescribed drugs in the cohort of cardiovascular patients based on data of electronic health records in 2019-2022 (*n* = 704).

Drug combination	n	Minor drug-drug interactions (P-List)
aspirin + indapamide	30	Indapamide will increase the level or effect of aspirin by acidic (anionic) drug competition for renal tubular clearance. Minor/ Significance Unknown
clopidogrel + torsemide	27	Clopidogrel increases levels of torsemide by decreasing metabolism. Minor/ Significance Unknown
hydrochlorothiazide + metformin	16	Hydrochlorothiazide decreases effects of metformin by pharmacodynamic antagonism. Minor/ Significance Unknown. Thiazide dosage >50 mg/ day may increase blood glucose
aspirin + hydrochlorothiazide	13	Hydrochlorothiazide will increase the level or effect of aspirin by acidic (anionic) drug competition for renal tubular clearance. Minor/ Significance Unknown
ascorbic acid + aspirin	12	Ascorbic acid will increase the level or effect of aspirin by acidic (anionic) drug competition for renal tubular clearance. Minor/Significance Unknown. ascorbic acid increases levels of aspirin by decreasing renal clearance. Minor/Significance Unknown
indapamide + magnesium hydroxide	10	Indapamide decreases levels of magnesium hydroxide by increasing renal clearance. Minor/Significance Unknown
carvedilol + omeprazole	8	Omeprazole will increase the level or effect of carvedilol by affecting hepatic enzyme CYP2C9/10 metabolism. Minor/Significance Unknown
indapamide + metformin	8	Indapamide decreases effects of metformin by pharmacodynamic antagonism. Minor/Significance Unknown. Thiazide dosage >50 mg/day may increase blood glucose
magnesium hydroxide + torsemide	7	Torsemide decreases levels of magnesium hydroxide by increasing renal clearance. Minor/Significance Unknown
aspirin + furosemide	6	Aspirin decreases effects of furosemide by pharmacodynamic antagonism. Minor/Significance Unknown. NSAIDs decrease prostaglandin synthesis
budesonide + torsemide	6	Budesonide, torsemide. Mechanism: pharmacodynamic synergism. Minor/Significance Unknown. Risk of hypokalemia, especially with strong glucocorticoid activity
indapamide + magnesium	6	Indapamide decreases levels of magnesium chloride by increasing renal clearance. Minor/Significance Unknown
magnesium hydroxide + spironolactone	6	Spironolactone increases levels of magnesium hydroxide by decreasing renal clearance. Minor/Significance Unknown
aspirin + eplerenone	4	Aspirin decreases effects of eplerenone by pharmacodynamic antagonism. Minor/Significance Unknown. NSAIDs decrease prostaglandin synthesis
atorvastatin + coenzyme Q10	4	Atorvastatin decreases levels of coenzyme Q10 by unspecified interaction mechanism. Minor/ Significance Unknown
formoterol + torsemide	4	Formoterol, torsemide. Mechanism: pharmacodynamic synergism. Minor/ Significance Unknown. Hypokalemia

furosemide + metformin	4	Furosemide increases levels of metformin by unspecified interaction mechanism. Minor/Significance Unknown. Patient should be closely observed for loss of blood glucose control; when drugs are withdrawn from a patient receiving metformin, patient should be observed closely for hypoglycemia
aspirin + chlorthalidone	3	Aspirin increases and chlorthalidone decreases serum potassium. Effect of interaction is not clear, use caution. Use Caution/Monitor
bisoprolol + tizanidine	3	Tizanidine increases effects of bisoprolol by pharmacodynamic synergism. Minor/Significance Unknown. Risk of hypotension
calcium carbonate + aspirin	3	Calcium carbonate, aspirin. Mechanism: passive renal tubular reabsorption due to increased pH. Minor/Significance Unknown. Salicylate levels increased at moderate doses; salicylate levels decreased at large doses (d/t increased renal excretion of unchanged salicylic acid)
captopril + tizanidine	3	Tizanidine increases effects of captopril by pharmacodynamic synergism. Minor/Significance Unknown. Risk of hypotension
B12 + omeprazole	3	Omeprazole decreases levels of cyanocobalamin by inhibition of GI absorption. Applies only to oral form of both agents. Minor/Significance Unknown
hydrochlorothiazide + magnesium	3	Hydrochlorothiazide decreases levels of magnesium hydroxide by increasing renal clearance. Minor/Significance Unknown
amiodarone + loratadine	2	Amiodarone will increase the level or effect of loratadine by affecting hepatic enzyme CYP2D6 metabolism. Minor/ Significance Unknown. amiodarone will increase the level or effect of loratadine by P-glycoprotein (MDR1) efflux transporter. Minor/ Significance Unknown
amlodipine + tizanidine	2	Tizanidine increases effects of amlodipine by pharmacodynamic synergism. Minor/Significance Unknown. Risk of hypotension
aspirin + ibuprofen	2	Aspirin will increase the level or effect of ibuprofen by acidic (anionic) drug competition for renal tubular clearance. Minor/ Significance Unknown
aspirin + meloxicam	2	Aspirin will increase the level or effect of meloxicam by acidic (anionic) drug competition for renal tubular clearance. Minor/Significance Unknown
captopril + tamsulosin	2	Aspirin will increase the level or effect of meloxicam by acidic (anionic) drug competition for renal tubular clearance. Minor/Significance Unknown
chlorthalidone + magnesium hydroxide	2	Chlorthalidone decreases levels of magnesium hydroxide by increasing renal clearance. Minor/Significance Unknown
choline magnesium trisalicylate + B12	2	Choline magnesium trisalicylate decreases levels of cyanocobalamin by inhibition of GI absorption. Applies only to oral form of both agents. Minor/ Significance Unknown
B12 + gabapentin	2	Gabapentin decreases levels of cyanocobalamin by inhibition of GI absorption. Applies only to oral form of both agents. Minor/ Significance Unknown
B12 + metformin	2	Metformin decreases levels of cyanocobalamin by unspecified interaction mechanism. Minor/Significance Unknown. It may take several years of metformin therapy to develop vitamin B12 deficiency
diclofenac + indapamide	2	Indapamide will increase the level or effect of diclofenac by acidic (anionic) drug competition for renal tubular clearance. Minor/ Significance Unknown

diclofenac + meloxicam	2	Diclofenac will increase the level or effect of meloxicam by acidic (anionic) drug competition for renal tubular clearance. Minor/ Significance Unknown
folic acid + metformin	2	Metformin decreases levels of folic acid by unspecified interaction mechanism. Minor/Significance Unknown
furosemide + magnesium hydroxide	2	Furosemide decreases levels of magnesium hydroxide by increasing renal clearance. Minor/Significance Unknown
ibuprofen + meloxicam	2	Ibuprofen will increase the level or effect of meloxicam by acidic (anionic) drug competition for renal tubular clearance. Minor/ Significance Unknown
metformin + nifedipine	2	Nifedipine increases levels of metformin by enhancing GI absorption. Applies only to oral form of both agents. Minor/Significance Unknown
metformin + potassium	2	Spironolactone increases levels of magnesium hydroxide by decreasing renal clearance. Minor/Significance Unknown
aceclofenac + aspirin	1	Aceclofenac will increase the level or effect of aspirin by acidic (anionic) drug competition for renal tubular clearance. Minor/Significance Unknown
aceclofenac + indapamide	1	Indapamide will increase the level or effect of aceclofenac by acidic (anionic) drug competition for renal tubular clearance. Minor/Significance Unknown
aceclofenac + lisinopril	1	Aceclofenac decreases effects of lisinopril by pharmacodynamic antagonism. Minor/Significance Unknown. NSAIDs decrease prostaglandin synthesis
amiodarone + celecoxib	1	Amiodarone will increase the level or effect of celecoxib by affecting hepatic enzyme CYP2C9/10 metabolism. Minor/Significance Unknown
amiodarone + diclofenac	1	Amiodarone will increase the level or effect of diclofenac by affecting hepatic enzyme CYP2C9/ 10 metabolism. Minor/ Significance Unknown
amiodarone + ibuprofen	1	Amiodarone will increase the level or effect of ibuprofen by affecting hepatic enzyme CYP2C9/ 10 metabolism. Minor/ Significance Unknown
amiodarone + meloxicam	1	Amiodarone will increase the level or effect of meloxicam by affecting hepatic enzyme CYP2C9/ 10 metabolism. Minor/ Significance Unknown
amiodarone + pyridoxine	1	Aspirin will increase the level or effect of ketorolac by acidic (anionic) drug competition for renal tubular clearance. Minor/Significance Unknown
aspirin + B12	1	Aspirin decreases levels of cyanocobalamin by inhibition of GI absorption. Applies only to oral form of both agents. Minor/Significance Unknown
aspirin + ketoprofen	1	Aspirin will increase the level or effect of ketoprofen by acidic (anionic) drug competition for renal tubular clearance. Minor/Significance Unknown
atorvastatin + loratadine	1	Atorvastatin will increase the level or effect of loratadine by P-glycoprotein (MDR1) efflux transporter. Minor/ Significance Unknown
budesonide + clarithromycin	1	Budesonide will decrease the level or effect of clarithromycin by affecting hepatic/ intestinal enzyme CYP3A4 metabolism. Minor/ Significance Unknown
budesonide + furosemide	1	Budesonide, furosemide. Mechanism: pharmacodynamic synergism. Minor/Significance Unknown. Risk of hypokalemia, especially with strong glucocorticoid activity
budesonide + metformin	1	Budesonide decreases effects of metformin by pharmacodynamic antagonism.

		Minor/Significance Unknown
captopril + lornoxicam	1	Lornoxicam decreases effects of captopril by pharmacodynamic antagonism. Minor/Significance Unknown. NSAIDs decrease prostaglandin synthesis
celecoxib + indapamide	1	Indapamide will increase the level or effect of celecoxib by acidic (anionic) drug competition for renal tubular clearance. Minor/Significance Unknown
celecoxib + meloxicam	1	Celecoxib will increase the level or effect of meloxicam by acidic (anionic) drug competition for renal tubular clearance. Minor/Significance Unknown
chlorthalidone + hydrochlorothiazide	1	Chlorthalidone will increase the level or effect of hydrochlorothiazide by acidic (anionic) drug competition for renal tubular clearance. Minor/ Significance Unknown
chlorthalidone + meloxicam	1	Chlorthalidone will increase the level or effect of meloxicam by acidic (anionic) drug competition for renal tubular clearance. Minor/Significance Unknown
chlorthalidone + metformin	1	Chlorthalidone decreases effects of metformin by pharmacodynamic antagonism. Minor/Significance Unknown. Thiazide dosage >50 mg/day may increase blood glucose
chlorthalidone + tizanidine	1	Tizanidine increases effects of chlorthalidone by pharmacodynamic synergism. Minor/Significance Unknown. Risk of hypotension
clarithromycin + apixaban	1	Clarithromycin will increase the level or effect of apixaban by affecting hepatic/ intestinal enzyme CYP3A4 metabolism. Minor/ Significance Unknown. Although clarithromycin is a combined P-gp and strong CYP3A4 inhibitor, the manufacturer has stated that pharmacokinetic data suggest that no dose adjustment is necessary with concomitant administration
diclofenac + flurbiprofen	1	Diclofenac will increase the level or effect of flurbiprofen by acidic (anionic) drug competition for renal tubular clearance. Minor/ Significance Unknown
diclofenac + lornoxicam	1	Diclofenac will increase the level or effect of lornoxicam by acidic (anionic) drug competition for renal tubular clearance. Minor/Significance Unknown
folic acid + methotrexate	1	Folic acid decreases effects of methotrexate by pharmacodynamic antagonism. Minor/Significance Unknown. Vitamin preparations containing folic acid or its derivatives may decrease responses to systemically administered methotrexate
formoterol + furosemide	1	Formoterol, furosemide. Mechanism: pharmacodynamic synergism. Minor/Significance Unknown. Hypokalemia
furosemide + folic acid	1	Furosemide decreases levels of folic acid by increasing renal clearance. Minor/Significance Unknown
furosemide + meloxicam	1	Meloxicam decreases effects of furosemide by pharmacodynamic antagonism. Minor/ Significance Unknown. NSAIDs decrease prostaglandin synthesis
ibuprofen + indapamide	1	Indapamide will increase the level or effect of ibuprofen by acidic (anionic) drug competition for renal tubular clearance. Minor/ Significance Unknowns
indapamide + meloxicam	1	Indapamide will increase the level or effect of meloxicam by acidic (anionic) drug competition for renal tubular clearance. Minor/ Significance Unknown
indapamide + tizanidine	1	Tizanidine increases effects of indapamide by pharmacodynamic synergism.

		Minor/Significance Unknown. Risk of hypotension
levothyroxine + omeprazole	1	Omeprazole decreases levels of levothyroxine by increasing gastric pH. Applies only to oral form of both agents. Minor/Significance Unknown. Conflicting evidence regarding this interaction exists
lisinopril + tizanidine	1	Tizanidine increases effects of lisinopril by pharmacodynamic synergism. Minor/Significance Unknown. Risk of hypotension
loratadine + nifedipine	1	Nifedipine will decrease the level or effect of loratadine by P-glycoprotein (MDR1) efflux transporter. Minor/ Significance Unknown
magnesium + torsemide	1	Torsemide decreases levels of magnesium hydroxide by increasing renal clearance. Minor/Significance Unknown
meloxicam + naproxen	1	Meloxicam will increase the level or effect of naproxen by acidic (anionic) drug competition for renal tubular clearance. Minor/ Significance Unknown
omeprazole + tizanidine	1	Omeprazole will decrease the level or effect of tizanidine by affecting hepatic enzyme CYP1A2 metabolism. Minor/Significance Unknown
potassium + vildagliptin	1	Potassium chloride increases effects of vildagliptin by pharmacodynamic synergism. Minor/ Significance Unknown. Interaction especially seen in the treatment of hypokalemia
spironolactone + tizanidine	1	Tizanidine increases effects of spironolactone by pharmacodynamic synergism. Minor/Significance Unknown. Risk of hypotension
tizanidine + torsemide	1	Tizanidine increases effects of torsemide by pharmacodynamic synergism. Minor/Significance Unknown. Risk of hypotension

***Note:** Impact of drug-drug interactions associated with the combinations 'aspirin + captopril' and 'aspirin + enalapril' may be considered insignificant due to the use of low-dose aspirin in the majority of cases. Administration of aspirin at doses less than 300 mg per day has little effect on the effectiveness of captopril and enalapril. Administration of aspirin in higher doses reduces the effectiveness of captopril and enalapril.
